# Effects of Saturated Fat, Polyunsaturated Fat, Monounsaturated Fat, and Carbohydrate on Glucose-Insulin Homeostasis: A Systematic Review and Meta-analysis of Randomised Controlled Feeding Trials

**DOI:** 10.1371/journal.pmed.1002087

**Published:** 2016-07-19

**Authors:** Fumiaki Imamura, Renata Micha, Jason H. Y. Wu, Marcia C. de Oliveira Otto, Fadar O. Otite, Ajibola I. Abioye, Dariush Mozaffarian

**Affiliations:** 1 Medical Research Council Epidemiology Unit, Institute of Metabolic Science, University of Cambridge School of Clinical Medicine, Cambridge Biomedical Campus, Cambridge, United Kingdom; 2 Tufts Friedman School of Nutrition Science & Policy, Boston, Massachusetts, United States of America; 3 George Institute for Global Health, The University of Sydney, Sydney Medical School, Camperdown, Australia; 4 Department of Epidemiology, Human Genetics & Environmental Sciences, The University of Texas Health Science Center at Houston, Houston, Texas, United States of America; 5 Department of Neurology, University of Miami Miller School of Medicine/Jackson Memorial Hospital, Miami, Florida, United States of America; 6 Department of Global Health and Population, Harvard T. H. Chan School of Public Health, Boston, Massachusetts, United States of America; Chinese University of Hong Kong, CHINA

## Abstract

**Background:**

Effects of major dietary macronutrients on glucose-insulin homeostasis remain controversial and may vary by the clinical measures examined. We aimed to assess how saturated fat (SFA), monounsaturated fat (MUFA), polyunsaturated fat (PUFA), and carbohydrate affect key metrics of glucose-insulin homeostasis.

**Methods and Findings:**

We systematically searched multiple databases (PubMed, EMBASE, OVID, BIOSIS, Web-of-Knowledge, CAB, CINAHL, Cochrane Library, SIGLE, Faculty1000) for randomised controlled feeding trials published by 26 Nov 2015 that tested effects of macronutrient intake on blood glucose, insulin, HbA1c, insulin sensitivity, and insulin secretion in adults aged ≥18 years. We excluded trials with non-isocaloric comparisons and trials providing dietary advice or supplements rather than meals. Studies were reviewed and data extracted independently in duplicate. Among 6,124 abstracts, 102 trials, including 239 diet arms and 4,220 adults, met eligibility requirements. Using multiple-treatment meta-regression, we estimated dose-response effects of isocaloric replacements between SFA, MUFA, PUFA, and carbohydrate, adjusted for protein, trans fat, and dietary fibre. Replacing 5% energy from carbohydrate with SFA had no significant effect on fasting glucose (+0.02 mmol/L, 95% CI = -0.01, +0.04; *n* trials = 99), but lowered fasting insulin (-1.1 pmol/L; -1.7, -0.5; *n* = 90). Replacing carbohydrate with MUFA lowered HbA1c (-0.09%; -0.12, -0.05; *n* = 23), 2 h post-challenge insulin (-20.3 pmol/L; -32.2, -8.4; *n* = 11), and homeostasis model assessment for insulin resistance (HOMA-IR) (-2.4%; -4.6, -0.3; *n* = 30). Replacing carbohydrate with PUFA significantly lowered HbA1c (-0.11%; -0.17, -0.05) and fasting insulin (-1.6 pmol/L; -2.8, -0.4). Replacing SFA with PUFA significantly lowered glucose, HbA1c, C-peptide, and HOMA. Based on gold-standard acute insulin response in ten trials, PUFA significantly improved insulin secretion capacity (+0.5 pmol/L/min; 0.2, 0.8) whether replacing carbohydrate, SFA, or even MUFA. No significant effects of any macronutrient replacements were observed for 2 h post-challenge glucose or insulin sensitivity (minimal-model index). Limitations included a small number of trials for some outcomes and potential issues of blinding, compliance, generalisability, heterogeneity due to unmeasured factors, and publication bias.

**Conclusions:**

This meta-analysis of randomised controlled feeding trials provides evidence that dietary macronutrients have diverse effects on glucose-insulin homeostasis. In comparison to carbohydrate, SFA, or MUFA, most consistent favourable effects were seen with PUFA, which was linked to improved glycaemia, insulin resistance, and insulin secretion capacity.

## Abstract 2

In a meta-analysis of feeding trials, Fumiaki Imamura and colleagues examine how dietary intake of fats and carbohydrate impact glucose-insulin homeostasis.

## Author Summary

**Why Was This Study Done?:**

- Effects of dietary fat and carbohydrate on metabolic health have been controversial, leading to confusion about specific dietary guidelines and priorities.
- To date there has not been a systematic evaluation of all available evidence to quantify the effects of dietary fat (saturated, monounsaturated, and polyunsaturated fat), and carbohydrate on various markers mediating the development of diabetes, including blood sugar, insulin sensitivity, and ability to produce insulin.

**What Did the Researchers Do and Find?:**

- We systematically identified and summarized findings of 102 randomised controlled trials, including a total of 4,660 participants, that provided meals varying in the types and levels of fat and carbohydrate to study participants and evaluated how such variations affected various measures of blood glucose control, insulin sensitivity, and ability to produce insulin.
- The findings suggest exchanging dietary carbohydrate with saturated fat does not appreciably influence markers of blood glucose control.
- On the other hand, substituting carbohydrate and saturated fat with a diet rich in unsaturated fat, particularly polyunsaturated fat, was beneficial for the regulation of blood sugar.

**What Do These Findings Mean?:**

- These findings may help inform scientists, clinicians, and the public on dietary priorities related to dietary fats and carbohydrates and metabolic health.
- This investigation suggests that consuming more unsaturated fats in place of either carbohydrates or saturated fats will help improve blood glucose control. Sole emphasis on lowering consumption of carbohydrates or saturated fats would not be optimal.
- Translated to foods, these findings support benefits of increasing consumption of vegetable oils and spreads, nuts, fish, and vegetables rich in unsaturated fats (e.g., avocado), in place of either animal fats or refined grains, starches, and sugars.

## Introduction

The prevalence of insulin resistance and type 2 diabetes is rising sharply in nearly all nations globally [1,2], highlighting the need for broad preventive therapies. Diet is a cornerstone of prevention and treatment in all major guidelines [3,4]. Dietary guidelines on macronutrient intake to improve glucose-insulin profiles and reduce or prevent type 2 diabetes generally recommend increasing foods rich in monounsaturated fat (MUFA) and reducing saturated fat (SFA) [3–6]. Yet these guidelines have also emphasized the major gaps in established evidence for effects of dietary fats and carbohydrate on glucose-insulin homeostasis, including uncertainty as to whether benefits of MUFA in some trials were confounded by caloric restriction and limited evidence on effects of either polyunsaturated fat (PUFA) or SFA [3–7]. Understanding the role of dietary macronutrients in glucose-insulin control is crucial to establishing informed guidelines for clinical providers and policy-makers around the world.

Prior knowledge has been limited by several factors, including focus on limited metrics to assess glucose-insulin homeostasis (e.g., fasting glucose alone), rather than studying multiple relevant outcomes, such as HbA1c, fasting insulin, insulin resistance, insulin secretion capacity, and post-challenge measures [8]; insufficient statistical power in many smaller trials to confirm important effects; and difficulties in evaluating results of individual trials due to multiple and varying changes in several macronutrients simultaneously [8–11]. Due to these challenges, the effects of dietary fats and carbohydrate on glucose-insulin homeostasis remains uncertain [8].

To address these critical gaps in knowledge, we performed a systematic review and dose-response meta-regression of randomised controlled feeding trials that tested the effects of isocaloric diets with differing composition of dietary macronutrients on multiple key metrics of fasting and post-challenge glucose-insulin homeostasis that represent degrees of glycaemia, insulin resistance, and insulin secretion capacity.

## Methods

### Eligibility Criteria and Literature Search

We developed the protocol (S1 Text) and conducted this study following Preferred Reporting Items for Systematic reviews and Meta-Analysis (PRISMA) guidelines [12] (S2 Text). Details of literature search and data preparation are provided in S3 Text. We systematically searched for randomised controlled feeding trials in adults (aged ≥18 y) examining diets varying in composition of specific fats and/or carbohydrate. Eligibility criteria included: provision of meals; comparison of isocaloric interventions; and assessment of relevant glucose-insulin metrics. We focused on outcomes commonly assessed in clinical research or practice [8,13], including fasting glucose, fasting insulin, haemoglobin A1c (HbA1c), homeostasis model assessment for insulin resistance (HOMA-IR, a fasting or post-challenge measure of insulin resistance calculated from glucose and insulin), C-peptide, 2 h post-oral-challenge glucose and insulin, and intravenous-infusion measures of Minmod-based insulin-sensitivity index (ISI) and acute insulin response (AIR) (gold-standard measures of insulin sensitivity and β-cell function, respectively) [8,13]. Study exclusions were insufficient information on macronutrient composition or glycaemic outcomes, studies of supplements or dietary advice only, and studies of acute (single meal) post-prandial effects only. We searched PubMed, EMBASE, OVID, BIOSIS, Web-of-Knowledge, CAB, CINAHL, Cochrane Library, SIGLE, and Faculty 1000, without language restriction, for all publications up until 26 November 2015. Search terms included each of the dietary macronutrients and metabolic measurements of interest. Titles and abstracts were screened by one investigator for eligibility; the full-text of potentially eligible reports was reviewed independently and in duplicate. Citation lists of included articles and identified prior reviews were similarly searched for relevant articles.

### Data Extraction

For each included trial, information was extracted independently (by FI, RM, JHYW, MCdOO, FOO, AIA) and in duplicate on first author, publication year, location, design, participant characteristics, dietary intervention, outcomes, compliance, and loss to follow-up. Any required information that was not reported was obtained by direct contact with authors (27 of 66 responded), other publications from the same trial, or trial-registry websites when available. Certain values were estimated using reported data: e.g., a mid-point was used if only a range was presented for age or body-mass index (BMI); in one trial, the reported consumption of rapeseed oil was combined with its macronutrient composition to estimate the intakes of specific dietary fats (S3 Text). Study quality was examined by using Jadad scale [14]: two authors independently scored each of the 11 quality-related items, calculated total scores of the 11 components and averaged two summed scores for each trial. Outcome measures presented in figures (e.g., insulin levels after glucose insulin) were digitalised to numeric information by two authors (FI and MCdOO) using software (Dagra, Blue Leaf Software Ltd., Hamilton, New Zealand), and two values for a single estimate were averaged.

### Meta-analysis

We evaluated the post-intervention values (means, standard errors) of trial arms as the primary outcomes. Changes in outcome values from baseline to endpoint were not used because certain procedures (intravenous tests) were often implemented only at endpoints and because baseline values were more subject to bias due to a carry-over effect in a crossover trial. When values were log-transformed, they were standardised to non-transformed values [15], except for HOMA-IR, which was standardised to log-transformed values. Between-arm correlations in trials using either crossover or Latin-square design were estimated and incorporated in meta-analysis by using reported *p*-values and outcome measures based on the function of within-individual correlations, interventional effects, their standard errors or deviations, and *p*-value [15,16]. Missing information on covariates (trans fat, dietary fibre), within-trial correlations, or precise post-intervention statistics (e.g., results expressed only as “*p* > 0.05”; standard deviations of post-intervention values [17]) was imputed with a multiple imputation approach to incorporate the uncertainty in our estimation by generating ten imputed datasets and pooling the estimates [18].

We estimated dose-response effects of replacement among carbohydrate, SFA, MUFA, and PUFA using multiple-treatments meta-regression (command: SAS PROC GLIMMIX, SAS Inc., North Carolina, United States) [19]. This meta-regression is an extension of a standard inverse-variance weighted model, expressed as Y_*ij*_ = I_*i*_ + SFA_*ij*_ × β_SFA_ + MUFA_*ij*_ × β_MUFA_ + PUFA_*ij*_ × β_PUFA_ + Covariates_*ijk*_ × β_k_ + ε_*ij*,_ modelling different macronutrients as multiple-treatment variables (SFA_ij_, MUFA_ij_, and PUFA_ij_) of trial *i*’s arm *j*, as well as study-specific intercepts (I_*i*_), arm-specific covariates *k* (protein, trans fat, dietary fibre), arm-specific standard errors of post-intervention values (ε_*ij*_, standard deviation_ij_ / √n_ij_), and their within-trial correlations based on trial design (r = 0.01–0.99 in crossover or Latin-square trials; r = 0 in parallel trials) specified in variance-covariance structure of ε_*ij*,_[16,20]. We used fixed-effects models, assessing both main effects and sources of heterogeneity (see below) [21]. In a stratum with a small number of trials, the model with five fixed-effects parameters was not fitted. We recognized the divergence of opinion on optimal weighting methods in the presence of statistical heterogeneity; in post hoc sensitivity analysis, we carried out random-effects meta-analyses (three τ^2^ for β_SFA_, β_MUFA_, and β_PUFA_, assumed to be independent) following stratification or restriction by significant sources of heterogeneity.

We evaluated SFA, MUFA, and PUFA (% energy) as main treatments, in comparison to isocaloric replacement with carbohydrate, by including each of these dietary fats in the model as well as intakes of protein (% energy) and trans fat (% energy) [9–11]. Effects of interchanging different fats were estimated by subtraction of corresponding regression coefficients (i.e., β_MUFA_–β_SFA_, β_PUFA_–β_SFA_, β_PUFA_–β_MUFA_) [20]. Because trans fat is a potential confounder not included in other meta-analyses of dietary fats [9,10], we extracted information on trans fat consumption in all trials reporting such data and imputed it within the remaining trials, with sensitivity analyses examining the effects of different methods for imputation and adjustment (S3 Text). To account for differences in carbohydrate quality between arms and trials, we also adjusted for dietary fibre intake (g/1,000 kcal) in each arm.

### Assessment of Heterogeneity, Sensitivity Analyses, and Small Study Bias

Hypothesizing that differences in effects of dietary macronutrients on fasting glucose, fasting insulin, HbA1c, and HOMA-IR would not be at random, we explored pre-specified potential sources of heterogeneity. These included study mean age (years), sex (% men), location (US/Canada, Europe/Australia, Asia), design (parallel, crossover/Latin-square), intervention duration (weeks), diabetes (yes/no), caloric restriction (yes/no), drop-out rate (%), participant blinding of meals provided (yes/no), mean BMI (kg/m^2^), mean baseline fasting glucose (mmol/L), mean fibre intake (g/1,000 kcal), mean weight change during intervention (kg), and study quality score (points). In post hoc analyses, we explored heterogeneity by extent of provision of all daily meals (full/partial). Each characteristic was tested as a potential source of heterogeneity by testing a standard Q-statistics for stratum-specific effects on the selected outcome for exchanging carbohydrate with SFA, MUFA, or PUFA, exchanging SFA with MUFA or PUFA, and exchanging MUFA with PUFA. For stratification by continuous variables, the median value across studies was used. To avoid false positive findings due to multiple testing of these exploratory interactions on the four outcomes, the α = 0.05 was adjusted for the family-wise false-discovery rate [22]. To minimize additional multiple comparisons, we explored potential interactions for the other outcomes (2 h glucose, 2 h insulin, ISI, AIR) only for those characteristics identified as significant sources of heterogeneity for fasting glucose, insulin, HbA1c, or HOMA, again adjusted for the false-discovery rate. Due to limited power, we did not explore heterogeneity for outcomes having ten or fewer trials (C-peptide).

We performed several sensitivity analyses for the main findings on fasting glucose, HbA1c, and fasting insulin, including varying the estimated between-arm correlation in crossover trials (S3 Text), repeating meta-analysis with and without adjustment for protein, fibre, and trans fat; using different methods for imputing and adjusting for trans fat; and adjusting for total caloric intake and for within-trial weight change to examine the potential mediating effect of macronutrient composition on energy metabolism [23,24] and between-arm imbalance in compliance to isocaloric intervention. In post hoc sensitivity analysis, we restricted to trials with follow-up ≥4 wk (the median of all trials), which may be especially relevant for longer-term measures such as HbA1c [25]; to trials using caloric-restriction, to explore whether this altered overall findings; and to trials with primary aims of varying either SFA, MUFA, or PUFA, to explore potential influence of combining trials with different original aims [9,20].

To assess publication bias or bias specific to small studies in multiple-treatment meta-regression, we utilized influence analyses [15]. Meta-regressions were repeated after excluding each single trial individually, with each new meta-regression finding plotted against the square root of the excluded trial’s effective sample size, accounting for within-trial correlations [26]. The resulting plots were inspected visually for patterns of bias by trial size; using linear regression to determine whether observed deviations were statistically significant, analogous to Egger’s test [15]; and using a non-parametric Wilcoxon rank test to examine whether estimates were symmetrical around the main estimate.

## Results

**Fig 1:**
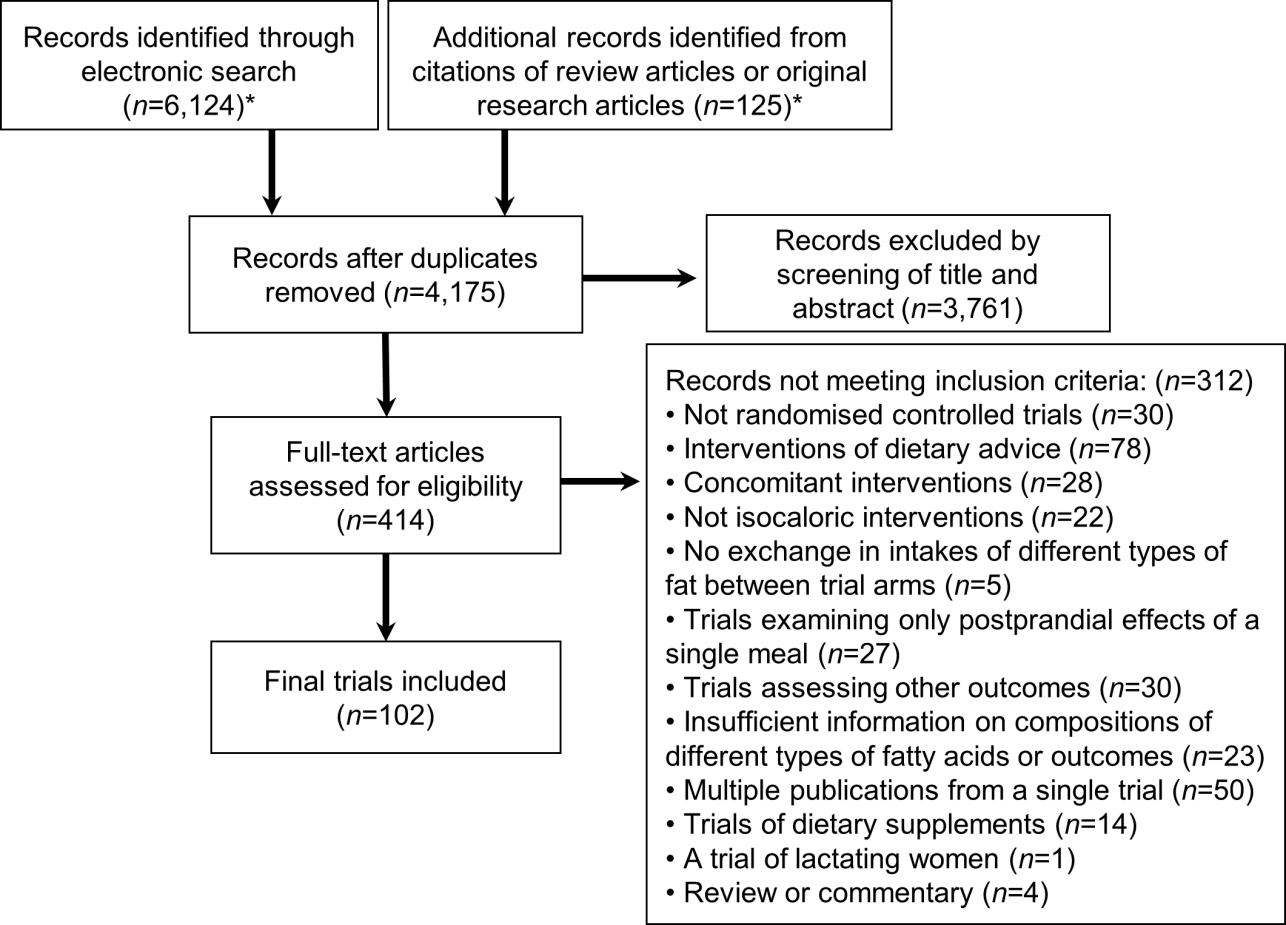
Flow diagram of systematic review of published trials evaluating effects of isocaloric replacement between macronutrient consumption on glucose homeostasis. *See S3 Text for details of the databases, eligibility criteria, search terms, and prior review articles.

Of 6,124 identified abstracts, 102 trials met inclusion criteria, evaluating a total of 4,220 unique subjects (45% male) across 239 dietary arms (Fig 1, Table 1, S1 Table, and S2 Table). Eleven trials implemented oral glucose or meal tolerance tests to assess 2 h post-challenge glucose or insulin; 13 trials, intravenous infusion tests to assess insulin sensitivity; and 10 trials, intravenous tests to assess insulin secretion capacity. No trials reported significant energy imbalance between arms after interventions. The average study quality was moderate to high (out of a possible score range of 0 to 11, median: 8.0, range: 4 to 10.5; see S2 Table).

**Table 1:** Characteristics of 102 randomised controlled feeding trials (total 239 intervention arms, 4,220 participants) evaluating effects of isocaloric replacement of dietary fats and carbohydrate on glucose-insulin homeostasis.*

Characteristics of trials or publications	*n* of trials or median (range)
Publication year	
2000 or earlier	31
2000 to 2009	38
2010 or later	33
Geographic area	
United States, Canada	35
Europe, Australia, New Zealand	57
Asia	7
Central or South America, Africa	3
Number of intervention arms	
2	76
3	21
4+	5
Design	
Parallel	33
Crossover/Latin square	67
Latin square	2
Feeding duration, days	28 (3–168)
Dietary intervention*	
Total energy, MJ/day	2,148 (1,000–3,466)
Carbohydrate, % energy	47.2 (5.0–65.0)
Saturated fat, % energy	9.2 (3.0–30.8)
Monounsaturated fat, % energy	13.6 (2.5–30.0)
Polyunsaturated fat, % energy	6.4 (2.0–21.4)
Protein, % energy	16.0 (10.1–33.0)
Trans fat, %	.6 (.0–3.4)
Fibre, g/4.2 MJ (1,000 kcal)	13.3 (5.5–24.4)
Caloric restriction, yes	18
Provided all meals (versus partial), yes	55
Blinding of participants, yes	62
Restricted to participants with diabetes, yes	31
*n* of participants per trial	
<25	55
25 to 49	26
≥50	21
Mean age of participants, years	
<30	18
30 to 49.9	29
≥50	55
Mean body mass index of participants, kg/m^2^	
<25	24
25 to 29.9	45
≥30	33
Mean fasting glucose, mmol/L	5.4 (4.0–11.9)
Mean glycated haemoglobin, %	7.4 (4.1–11.9)
Mean weight change during follow-up, kg	-0.5 (-11.8–2.7)
Overall study quality score †	8.0 (4.0–10.5)

* Intervention arms and control arms combined.

**†** Possible range 0 to 11 (see S2 Table for details).

### Fasting Glucose, HbA1c, and 2 h Glucose

Ninety-nine trials including 237 dietary arms evaluated fasting glucose. In pooled analysis, each 5% energy exchange of carbohydrate with SFA, MUFA, or PUFA did not significantly alter fasting glucose levels (*p* > 0.16 each) (Table 2). Exchanges between SFA, MUFA, and PUFA also did not alter fasting glucose (*p* > 0.15 each), except for the replacement of SFA with PUFA, which was linked to a decrease in fasting glucose levels (-0.04 mmol/L; 95% CI: -0.07, -0.01; *p* = 0.028).

**Table 2:** Effects of isocaloric replacements between carbohydrate (CHO), saturated fat (SFA), monounsaturated fat (MUFA), and polyunsaturated fat (PUFA) on metrics of glucose-insulin homeostasis in randomised controlled feeding trials.*

Outcome	*n* trials (arms)	*n* adults	Effects (95% CI) of isocaloric replacement of 5% dietary energy					
			CHO	CHO	CHO	SFA	SFA	MUFA
			→SFA	→MUFA	→PUFA	→MUFA	→PUFA	→PUFA
Glucose, mmol/L	99 (237)	4,144	0.02	0.00	-0.02	-0.02	-0.04	-0.02
			(-0.01, 0.04)	(-0.02, 0.02)	(-0.05, 0.01)	(-0.04, 0.00)	(-0.07, -0.01)*	(-0.05, 0.01)
2 h glucose, mmol/L†	11 (29)	615	-0.04	-0.15	0.21	-0.10	0.26	0.36
			(-0.39, 0.31)	(-0.76, 0.47)	(-0.35, 0.78)	(-0.91, 0.70)	(-0.34, 0.85)	(-0.48, 1.20)
Haemoglobin A1c, %	23 (54)	618	0.03	-0.09	-0.11	-0.12	-0.15	-0.03
			(-0.02, 0.09)	(-0.12, -0.05)***	(-0.17, -0.05)***	(-0.19, -0.05)***	(-0.23, -0.06)***	(-0.09, 0.03)
Insulin, pmol/L	90 (216)	3,774	-1.1	0.1	-1.6	1.2	-0.5	-1.6
			(-1.7, -0.5)**	(-0.3, 0.4)	(-2.8, -0.4)*	(0.6, 1.8)***	(-2.0, 1.1)	(-2.8, -0.5)*
2 h insulin, pmol/L†	11 (28)	598	1.9	-20.3	-24.9	-22.2	-26.8	-4.6
			(-19.3, 23.1)	(-32.2, -8.4)**	(-53.9, 4.1)	(-49.1, 4.6)	(-72.5, 18.9)	(-33.3, 24.1)
C-peptide, nmol/L	7 (16)	175	0.03	0.02	-0.05	-0.01	-0.07	-0.06
			(0.00, 0.05)*	(-0.01, 0.04)	(-0.11, 0.02)	(-0.03, 0.01)	(-0.14, -0.01)*	(-0.14, 0.01)
HOMA-IR, % change	30 (76)	1,801	0.7	-2.4	-3.4	-3.1	-4.1	-1.0
			(-1.6, 3.1)	(-4.6, -0.3)*	(-5.9, -0.8)*	(-5.8, -0.4)**	(-6.4, -1.6)*	(-4.4, 2.6)
Insulin sensitivity index, 10^−5^/(pmol/L)/min‡	13 (38)	1,292	-0.10	-0.01	0.14	0.08	0.24	0.16
			(-0.21, 0.02)	(-0.11, 0.08)	(-0.14, 0.43)	(-0.01, 0.17)	(-0.13, 0.61)	(-0.20, 0.52)
Acute insulin response, pmol/L/min‡	10 (29)	1,204	-0.02	-0.03	0.49	-0.01	0.51	0.52
			(-0.11, 0.07)	(-0.07, 0.01)	(0.17, 0.80)**	(-0.08, 0.06)	(0.20, 0.82)**	(0.21, 0.82)**

*Values represent the pooled mean change (95% CI) for isocaloric exchange of the specified macronutrients, with the other macronutrients held constant. All analyses adjusted for between-arm differences in protein (% energy), trans-fat (% energy), and dietary fibre (g/1000 kcal) within each trial. 1 mg/dL glucose = 0.0555 mmol/L; 1 mU/L insulin = 6 pmol/L; HbA1 mmol/mol = (HbA1c % - 2.15)×10.929.

* *p* < 0.05

** *p* < 0.01

*** *p* < 0.001.

† Oral glucose tolerance tests evaluating post-prandial glucose levels after ingestion of a test meal or drink.

‡ Positive values for the insulin sensitivity index (Minimal Model) and acute insulin response, derived from intravenous infusion tests, indicate improvement of insulin sensitivity and insulin secretion capacity, respectively.

Among 23 trials including 54 dietary arms and assessing HbA1c, isocaloric replacement of 5% dietary energy from either carbohydrate or SFA with 5% dietary energy from either MUFA or PUFA lowered HbA1c (*p* < 0.001 each) (Table 2). In eleven trials assessing 2 h post-challenge glucose no significant effects of macronutrient exchanges were identified.

### Insulin, Insulin Sensitivity, and Insulin Secretion

Ninety trials including 216 arms evaluated fasting insulin (Table 2). Compared with 5% dietary energy from carbohydrate, 5% dietary energy from either SFA or PUFA reduced fasting insulin by 1.1 pmol/L (0.6, 1.6; *p* = 0.001) and 1.6 pmol (0.4, 2.8; *p* = 0.015), respectively, while replacement with MUFA had no significant effect (0.1 pmol/L; -0.03, 0.04; *p* = 0.001). However, replacement of carbohydrates with MUFA was linked to increased fasting insulin (+1.2 pmol/L; 0.6, 1.8; *p* = 0.001). In 11 trials evaluating 2 h post-challenge insulin, replacement of carbohydrate or SFA with MUFA or PUFA did not significantly reduce the fasting insulin levels; while replacing MUFA with carbohydrate significantly lowered 2 h insulin (-20.3 pmol/L; -32.2, -8.4; *p* = 0.001). In 7 trials, consuming SFA in place of carbohydrate significantly increased C-peptide (0.03 nmol/L; 0.00, 0.05; *p* = 0.024).

The effects on HOMA-IR of consuming MUFA or PUFA in place of carbohydrate or SFA (30 trials) were generally similar to findings for fasting glucose, HbA1c, and 2 h insulin. For example, consuming 5% energy from PUFA in place of carbohydrate or SFA lowered HOMA-IR by 3.4% (0.8, 5.9%; *p* = 0.010) and 4.1% (1.6, 6.4%; *p* = 0.001), respectively.

Intravenous gold-standard measures of insulin sensitivity (ISI) and insulin secretion capacity (AIR) were assessed in 13 trials and 10 trials, respectively (Table 2). No significant effects of macronutrient replacements were seen for ISI. In comparison, AIR significantly improved with the consumption of PUFA, whether in place of carbohydrate, SFA, or even MUFA (*p* < 0.004 each).

### Exploration of Heterogeneity

**Fig 2:**
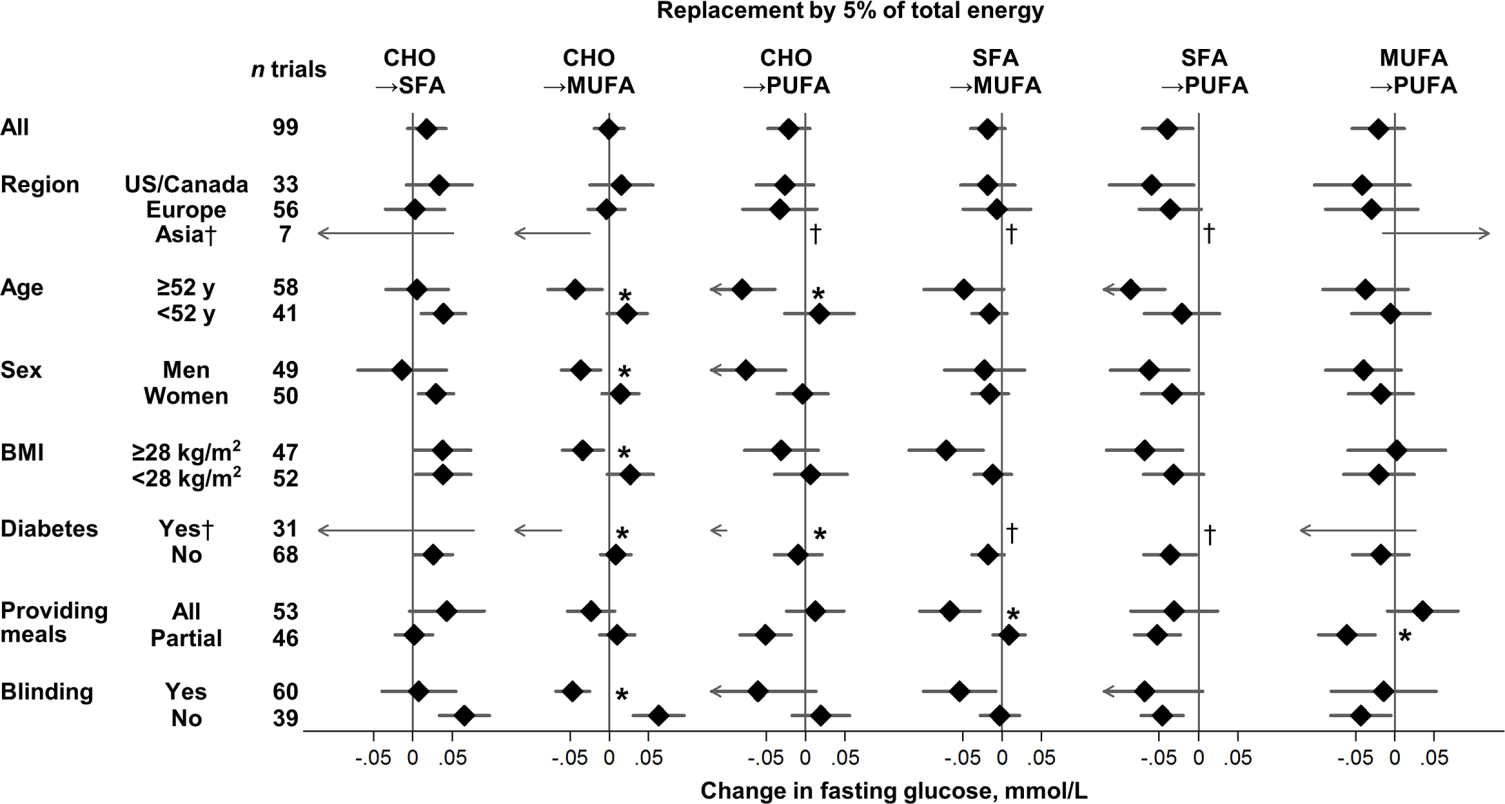
Effects on fasting glucose of isocaloric replacements between carbohydrate (CHO), saturated fat (SFA), monounsaturated fat (MUFA), and polyunsaturated fat (PUFA) in randomised controlled feeding trials. Values represent pooled mean effects (95% CI) of specified macronutrient replacements, with other macronutrients held constant. *Significant heterogeneity across strata after correction for false-discovery rate (exploration of multiple characteristics for heterogeneity). †Estimates not shown due to wide 95% CIs; see S3 Table for numeric information. 1 mg/dL = 0.0555 mmol/L.

For effects on fasting glucose, several sources of heterogeneity were identified (Fig 2, S3 Table). MUFA, compared with carbohydrate, lowered fasting glucose to a greater extent in trials with blinded participants and in trials recruiting adults with diabetes, older age, men, or higher BMI (*p* heterogeneity < 0.004 each). Older age and presence of diabetes also strengthened glucose-lowering effects of PUFA (*p* heterogeneity < 0.002 each).

**Fig 3:**
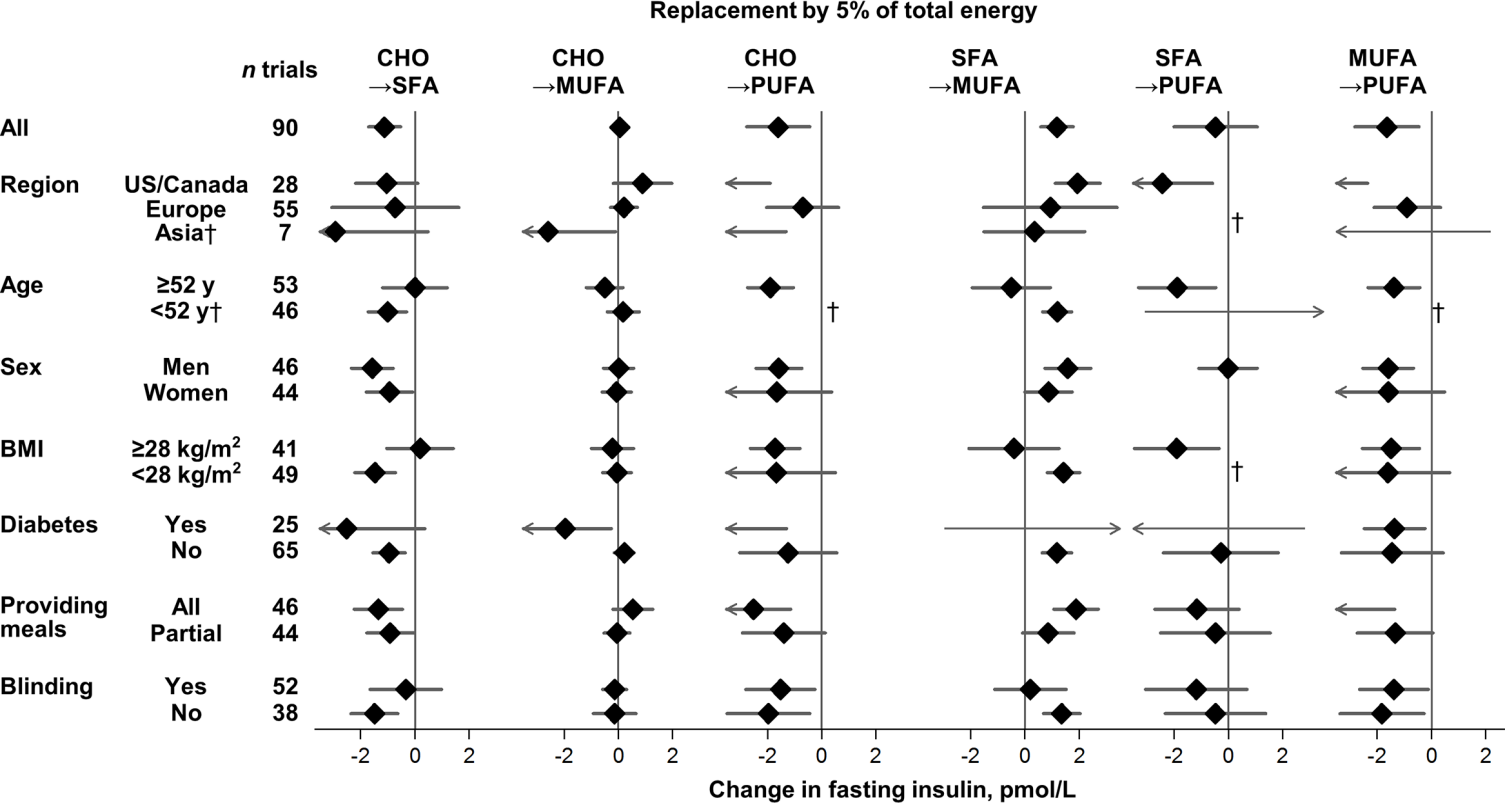
Effects on fasting insulin of isocaloric replacements between carbohydrate (CHO), saturated fat (SFA), monounsaturated fat (MUFA), and polyunsaturated fat (PUFA) in randomised controlled feeding trials. Values represent pooled mean effects (95% CI) of specified macronutrient replacements, with other macronutrients held constant. No significant sources of heterogeneity were detected. †Estimates not shown due to wide, 95% CIs; see S3 Table for numeric information. 1 μIU/mL = 6 pmol/L.

Effects on fasting glucose appeared possibly smaller in trials without participant blinding, although these differences were not statistically significant (false-discovery corrected). Replacing carbohydrate with MUFA reduced fasting glucose in participant-blinded trials; but increased fasting glucose in participant-unblinded trials (*p* heterogeneity < 0.001). In post hoc analyses, whether trials provided all or partial meals did not consistently influence the direction or strength of various findings. No significant sources of heterogeneity were observed for effects of macronutrients on fasting insulin (Fig 3).

The HbA1c-lowering effect of PUFA, compared with SFA, was significantly larger in North American than European trials (*p* heterogeneity < 0.0001) (S3 Table); yet despite the statistical heterogeneity, the direction of effects was the same. No other significant sources of heterogeneity were observed for effects of macronutrients on HbA1c or HOMA-IR.

### Sensitivity Analyses and Small Study Bias

To evaluate robustness of the main findings, we repeated meta-analyses using random effects in five selected strata, which were significant sources of heterogeneity: trials conducted in Western nations; trials of adults with diabetes; trials of adults without diabetes; trials providing whole meals; and trials with blinding of meals provided (S4 Table). Findings using random effects were generally similar, with some results having wider CIs and failing to achieve statistical significance (e.g., for HbA1c); most results being statistically significant in both fixed-effects and random-effects models, in particular for 2 h insulin, HOMA-IR, and AIR; and rarely some findings being significant in random-effects but not fixed-effects models. Other sensitivity analyses also supported robustness of our main findings, including evaluating a range of assumed between-arm correlations in crossover or Latin-square trials (S1 Fig) and altering model covariates, imputation methods for trans fat, and restrictions on trial subtypes (S5 Table). For example, while a smaller subset of trials (31 of 102) specifically aimed to achieve major variation in PUFA, analysis restricted to these trials showed generally similar findings, with wider confidence intervals, as the primary analyses. We also identified little evidence for small study bias based on influence analysis tested by linear regression (analogous to Egger’s test: *p* > 0.24 each) or non-parametric Wilcoxon rank tests (*p* > 0.28 each) (S2 Fig).

## Discussion

The results of this systematic review and meta-analysis of randomised controlled feeding trials provide, to our knowledge, the most robust available evidence for the effects of dietary fats and carbohydrate on diverse glucose-insulin metrics. We identified divergent relationships of specific dietary fats with different measures of glucose-insulin homeostasis. For example, only energy intake substitution with PUFA was linked to lower fasting glucose, lower HbA1c, improve HOMA-IR, and improve insulin secretion capacity. These effects were generally seen whether PUFA replaced carbohydrate or SFA; interestingly, insulin secretion capacity also improved when PUFA replaced MUFA. In comparison, MUFA consumption did not appear to significantly influence fasting glucose, compared to others macronutrients; but was seen to reduce HbA1c and improve HOMA-IR in comparison to either carbohydrate or SFA. Exchange of SFA for carbohydrate had little observed effects on most measures, except for reduced fasting insulin and a borderline significant effect on C-peptide.

These findings help inform dietary guidance on macronutrients to influence metabolic health. Currently, major organizations recommend that SFA be replaced with MUFA or PUFA, largely to improve lipid profiles rather than glucose-insulin metrics, for the primary and secondary prevention of diabetes [3,4]. Our investigation of trials with relatively short average duration (28 d) suggests that consuming more unsaturated fats (MUFA, PUFA) in place of either carbohydrate or SFA may improve HbA1C and HOMA-IR; and that focusing on PUFA in particular may have additional benefits on insulin secretion capacity. The comparatively similar effects of SFA versus carbohydrate on glucose-insulin homeostasis are consistent with their similar overall associations with both incident diabetes and cardiovascular events [27]. Translated to foods, these finding support increased consumption of vegetable oils and spreads, nuts, fish, and vegetables rich in unsaturated fats (e.g., avocado), in place of either animal fats or refined grains, starches, and sugars.

The magnitudes of the observed effects deserve consideration. For example, for each 5% energy of increased MUFA or PUFA, HbA1c improved by approximately 0.1%. Based on the relationship between HbA1c and clinical events, a 0.1% reduction would be estimated to reduce the incidence of type 2 diabetes by 22.0% (95% CI = 15.9, 28.4%) [28] and cardiovascular diseases by 6.8% (1.3, 13.0%) [29]. Such an effect could clearly be clinically meaningful, especially given the current global pandemic of type 2 diabetes [1,2].

While both MUFA and PUFA similarly improve blood lipid profiles [9,10], their associations with clinical cardiovascular events are less similar [27]. Due to these differences, the US Dietary Guidelines Advisory Committee concluded that strong evidence exists for cardiovascular benefits of PUFA, but limited evidence for cardiovascular benefits of MUFA [30]. Given the similar effects of these unsaturated fats on blood lipids, the present investigation may partly elucidate why PUFA might have greater overall cardiovascular benefits, given its additional benefits on fasting glucose and insulin secretion capacity, key pathological markers for development and progression of metabolic disease. The independence of these benefits, whether PUFA replaces carbohydrate or SFA (or for insulin secretion capacity, even MUFA), is consistent with growing evidence for specific cardiometabolic benefits of PUFA, regardless of the replacement nutrient [31,32].

Biologic plausibility of these findings is supported by experimental evidence that PUFA suppresses oxidative stress, hepatic lipogenesis and steatosis, pancreatic lipotoxicity, and insulin resistance [33–37]. PUFA may also help counter toxicity of tissue free fatty acids [35]; and increase membrane fluidity, which might augment insulin sensitivity and lower risk of type 2 diabetes [38,39]. These effects have been seen with omega-6 linoleic acid, the predominant PUFA (generally 90%+ of total PUFA), rather than only omega-3 PUFA. Meta-analyses of omega-3 supplementation as well as dietary intakes and blood biomarker levels of omega-3 PUFA demonstrate no significant effects on fasting glucose or incident diabetes [40,41]. Together with our results, these findings suggest that metabolic benefits of PUFA relate to omega-6 PUFA or total PUFA, and not omega-3 PUFA alone.

Compared with PUFA (consumed from a small number of vegetable oils and nuts), MUFA derives from diverse types of foods including red meats, dairy, nuts, and vegetable oils. Cardiometabolic effects of these different foods vary widely [27]: red meats and especially processed meats appear to increase risk of diabetes; milk, cheese, and yogurt appear relatively neutral or modestly beneficial; while specific plant sources of MUFA, such as nuts and virgin olive oil, have cardiometabolic benefits [27,42,43]. In the present investigation, most trials that sought to increase MUFA consumption did so via increased plant sources (olive oil, canola oil, sunflower oil, nuts); trials that lowered MUFA generally did so by lowering animal fats (which contain both SFA and MUFA). Thus, effects of altering MUFA consumption could vary depending on the food source. Yet, in all these foods, the MUFA molecule is identical (nearly entirely [>95%] oleic acid), so that if effects vary by food source, it should be due to other compounds in these foods (e.g., phenolics in nuts and oils; haeme iron in meats; probiotics in yogurt), rather than different effects of plant- versus animal-origin MUFA per se.

Our findings for SFA are consistent with observed relationships with incident diabetes and clinical cardiovascular events. Compared to the average background diet (predominantly carbohydrates), SFA consumption is not associated with risk of incident diabetes in long-term cohorts [44]; nor did reduction of SFA, when replaced with carbohydrate, alter risk of incident diabetes in the Women’s Health Initiative randomised trial [45]. Because diabetes and insulin resistance are major risk factors for cardiovascular disease, our findings also support and help explain meta-analyses demonstrating no association of overall SFA consumption, when compared with the average background diet or total carbohydrate, with risk of coronary heart disease or stroke [30,46].

In vitro, even-chain SFA, including myristic acid (14:0) and palmitic acid (16:0), activates pro-inflammatory cascades, induces skeletal muscle insulin resistance, and damages pancreatic β-cells, while the MUFA oleic acid (18:1) may partly protect against some of these effects [35,47–49]. However, in vivo, dietary SFA and MUFA may be readily oxidized as energy sources [50,51], while tissue levels of major SFA and MUFA may be at least equally influenced by endogenous hepatic synthesis of fatty acids rather than direct dietary intake [52]. This explains why dietary starch and sugars, which activate hepatic de novo lipogenesis, are positively associated with blood levels of major SFA and MUFA [52–54]. Thus, effects of blood and tissue SFA and MUFA may not inform and should be separately considered from biologic effects of dietary SFA and MUFA.

In exploratory analyses, we identified some sources of potential heterogeneity in effects of dietary macronutrients. The most compelling interactions, based on consistency across different measures and with reasonably large numbers of trials in each subgroup, were for stronger benefits of MUFA and PUFA on fasting glucose among older adults and patients with prevalent diabetes. Both our identified and null findings for heterogeneity should be interpreted with caution: absence of significant heterogeneity could result from insufficient power (e.g., by region, trials in non-Western countries were scarce), while positive interaction could result from chance, even corrected for false-discovery. Our findings advance the field by exploring interactions using all currently available data from feeding trials, which generate hypotheses to be tested in new studies, including studies of gene-diet interactions across diverse populations, controlled trials of glucose-insulin biomarkers, and prospective studies of clinical events.

Our investigation has several strengths. Our systematic search, rigorous screening, and data extraction protocols made it unlikely that any large studies or relevant data were missed or erroneously extracted. In addition, the large number of identified studies makes it unlikely that any single study, whether included or missed, would appreciably alter our findings. We focused on randomised, controlled trials using feeding interventions, maximizing inference for true biological effects. We examined different replacement scenarios among major macronutrients, providing novel insights for the most relevant replacements; confirmed robustness of our findings in sensitivity analyses and adjusted for between-arm differences in protein, trans fat, and dietary fibre, reducing the influence of variation in these factors. We evaluated multiple relevant metrics, including fasting, post-prandial, and long-term glycaemia, insulin levels, and insulin resistance, providing a more comprehensive picture of the full effects of dietary macronutrients.

Potential limitations should be considered. While feeding trials maximize inference for biologic effects, the findings may not be generalisable to effects of dietary advice, which can be influenced by knowledge and compliance, and to effects of long-term habitual diet. Conversely, we found little evidence for heterogeneity by duration of intervention ranging from 3 to 168 d, and our overall findings are consistent with meta-analyses of incident diabetes and clinical cardiovascular events. While all trials were randomised, not all were double blind; yet, food-based dietary trials are often, by necessity, challenging to blind for participants. This importance was implicated in our study because replacing SFA or carbohydrate with MUFA was shown to lower fasting glucose, 2 h glucose, 2 h insulin and HOMA-IR in trials implementing blinding intervention but not in trials not blinding for participants. Sufficient information was not available to classify subtypes of fatty acids, so our findings should be considered most relevant to effects of total dietary SFA (predominantly palmitic acid), total PUFA (predominantly linoleic acid), total MUFA (almost entirely oleic acid), and total carbohydrate (mostly refined starch and sugars). For instance, our results should not be extrapolated to potential effects of carbohydrate in fruit, legumes, or minimally processed whole grains. Trials inconsistently provided information on food sources of macronutrients (e.g., specific oils) or cooking methods; future studies should evaluate whether these characteristics modify physiologic effects. Most trials were in North America and Europe, and findings may not be generalisable to other world regions. Our analysis evaluated relatively few trials measuring C-peptide, post-challenge glucose and insulin, ISI, and AIR, and did not evaluate outcomes specific to peripheral or hepatic insulin sensitivity, not capturing the potential effects of fatty acids on insulin sensitivity of specific tissues. Unmeasured sources of heterogeneity may exist, such as effects of genes and cooking methods. Therefore, our meta-analysis highlights the gaps in knowledge for potential effect-modifiers for various metrics of glucose-insulin homeostasis. Our results and available evidence support the importance of further experimental studies and large, adequately powered feeding trials examining ISI and AIR. Meta-analyses can be influenced by small study bias; yet, influence analysis did not support the presence of such bias, and findings for our main endpoints were based on large numbers of trials, making it unlikely that inclusion of any unpublished trials would substantially alter the results.

In conclusion, this systematic review and meta-analysis provides novel quantitative evidence for effects of major dietary fats and carbohydrate on glucose-insulin homeostasis. The results support guidelines to increase MUFA intake to improve glycaemia and insulin resistance, with possibly stronger effects among patients with type 2 diabetes, and to increase PUFA intake in the general population to improve long-term glycaemic control, insulin resistance, and insulin secretion capacity, in place of SFA or carbohydrate. These findings help inform public health and clinical dietary guidelines to improve metabolic health.

## Supporting Information

S1 Fig Effects of isocaloric macronutrient exchange by 5% of total energy intake on (A) fasting glucose, (B) haemoglobin A1c, and (C) fasting insulin under different assumption of a between-arm correlation in crossover or Latin-square trials.
(PDF)

S2 Fig Assessment for small study bias in meta-regression using influence analysis, evaluating effects of isocaloric exchange of 5% energy between different macronutrients on (A) fasting glucose, (B) haemoglobin A1c, and (C) fasting insulin.
(PDF)

S1 Table Characteristics of 102 randomised controlled feeding trials evaluated in the meta-analysis of effects of diets with different macronutrient compositions on glucose-insulin homeostasis. (PDF)

S2 Table Characteristics and scores of reporting quality of 102 trials eligible for the meta-analysis of randomised controlled feeding trials of macronutrient intakes and glycaemic outcomes. (PDF)

S3 Table Effects of isocalorically exchanging 5% of dietary energy between carbohydrate and major dietary fats on glucose-insulin metrics, with stratification by country, age, sex, diabetes status, provision of meals, and blinding in randomised controlled feeding trials. (PDF)

S4 Table Effects of isocalorically exchanging 5% of dietary energy between carbohydrate and major dietary fats on glucose-insulin metrics: fixed-effects and random-effects meta-analyses by region, diabetes status, provision of meals, and blinding in randomised controlled feeding trials. (PDF)

S5 Table Effects of isocalorically exchanging 5% of dietary energy between carbohydrate and major dietary fats on fasting glucose, haemoglobin A1c, and fasting insulin: sensitivity meta-analysis concerning model covariates and study characteristics. (PDF)

S1 Text Protocol of a systematic review and meta-analysis of effects of macronutrient replacement on glucose-insulin homeostasis. (PDF)

S2 Text PRISMA 2009 Checklist. (PDF)

S3 Text Eligibility criteria, literature search, data preparation, imputation, and reference list. (PDF)

## Abbreviations

AIR: acute insulin response
BMI: body-mass index
CHO: carbohydrate
HOMA-IR: homeostasis model assessment for insulin resistance
ISI: insulin-sensitivity index
MUFA: monounsaturated fat
PUFA: polyunsaturated fat
SFA: saturated fat
