# Supplementary material for: Effects of Saturated Fat, Polyunsaturated Fat, Monounsaturated Fat, and Carbohydrate on Glucose-Insulin Homeostasis: A Systematic Review and Meta-analysis of Randomised Controlled Feeding Trials
Source: PLoS Med. 2016 Jul 19;13(7):e1002087. doi: 10.1371/journal.pmed.1002087 (PMC4951141; doi:10.1371/journal.pmed.1002087)
Supplement: S1 Fig — Effects of isocaloric macronutrient exchange by 5% of total energy intake on (A) fasting glucose, (B) haemoglobin A1c, and (C) fasting insulin under different assumption of a between-arm correlation in crossover or Latin-square trials. (PDF) [file pmed.1002087.s001.pdf]

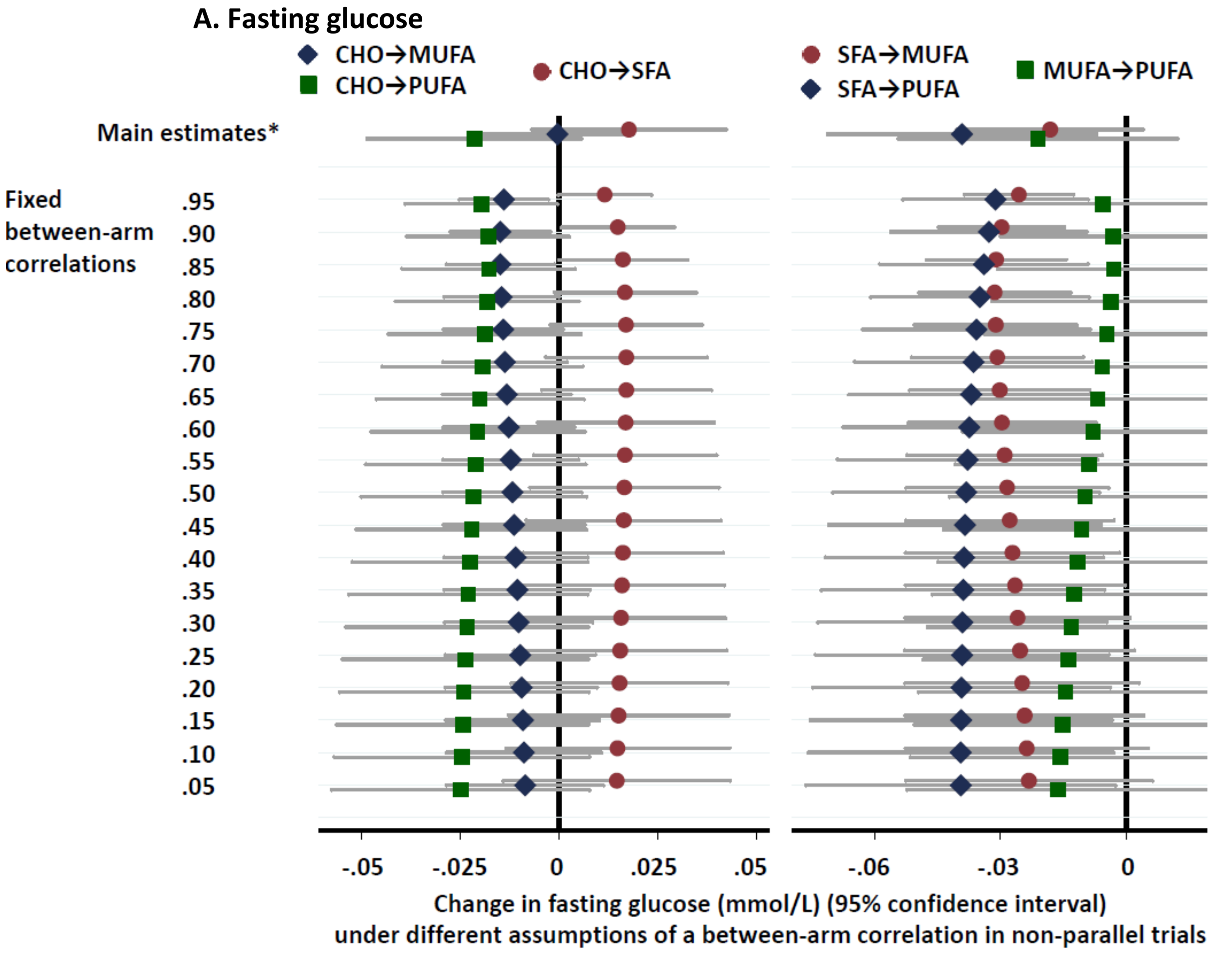

S1 Fig. Effects of isocaloric macronutrient exchange by 5% of total energy intake on A) fasting glucose, B) haemoglobin A1c, and C) fasting insulin under different assumption of a between-arm correlation in crossover or Latin-square trials. 1 mg/dL=0.0555 mmol/L for glucose, 1 µIU/mL=6 pmol/L, HbA1mmol/mol=(HbA1c % - 2.15)×10.929. In trials without a parallel design, within-person correlations of outcomes should be modelled. Many publications gave sufficient information to calculate within-person correlations (r mean=0.74), but not all. Prior to the main analyses, missing information on correlations was imputed by regression approach prior to main meta-analysis. Imputation was not likely to cause substantial impact on overall results according to sensitivity analysis presented here. This figure confirms results were generally stable, if we used a single correlation (between-arm correlations =0.05 to 0.95, as presented) in all non-parallel trials. Abbreviations: CHO, carbohydrates; MUFA, monounsaturated fatty acids; PUFA, polyunsaturated fatty acids; and SFA, saturated fatty acids.

B. Haemoglobin A1c

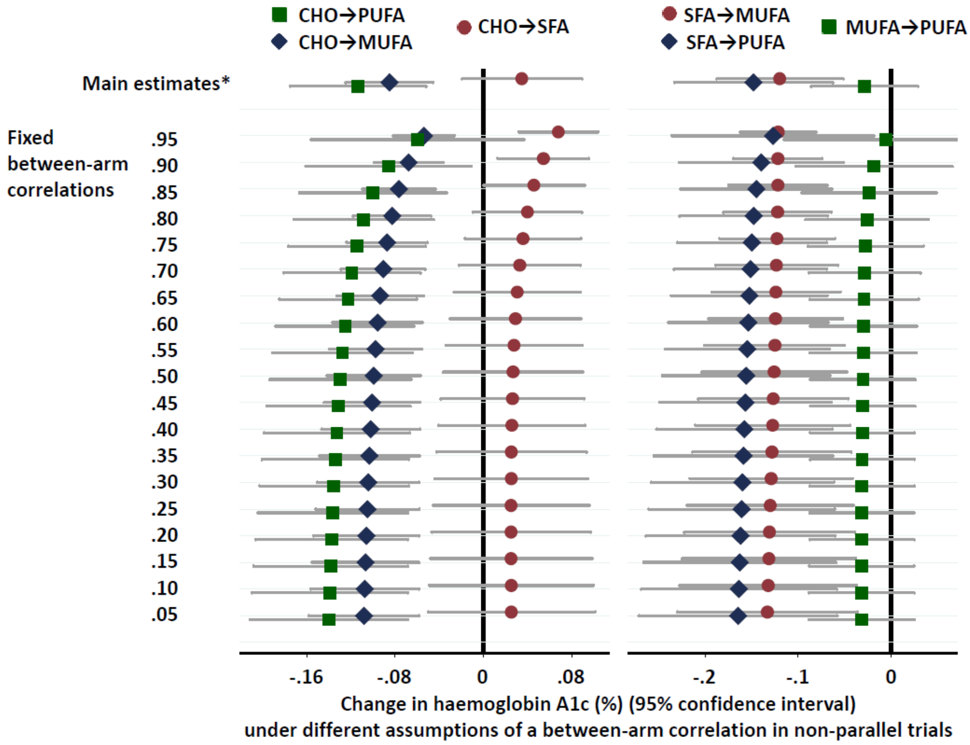

C. Fasting insulin

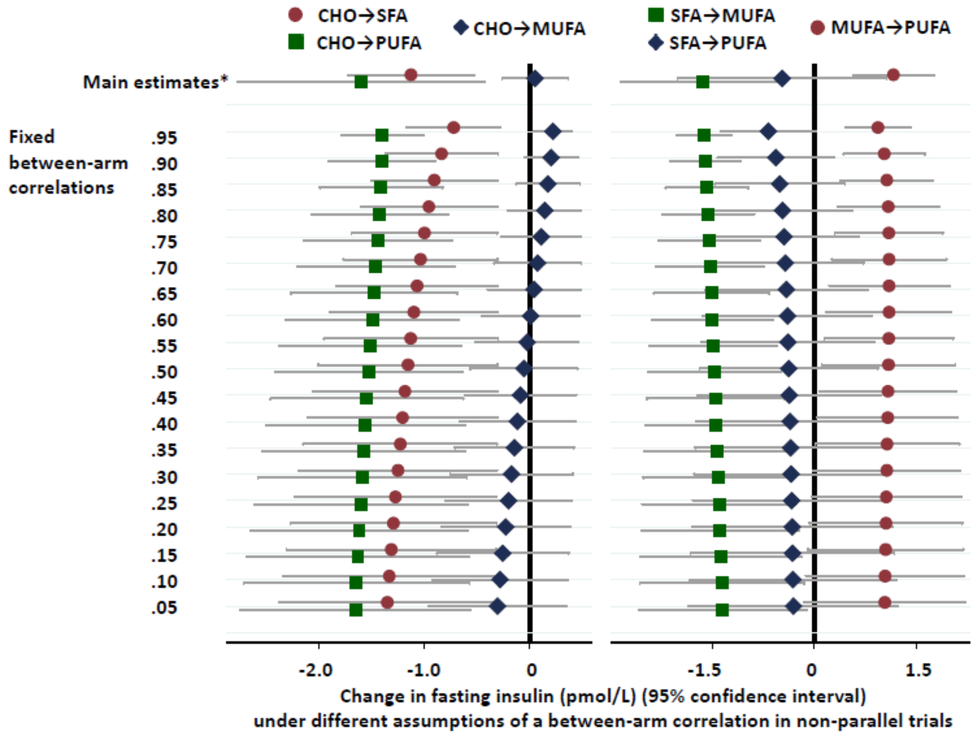

S1 Fig (continued).
