# Supplementary material for: Effects of Saturated Fat, Polyunsaturated Fat, Monounsaturated Fat, and Carbohydrate on Glucose-Insulin Homeostasis: A Systematic Review and Meta-analysis of Randomised Controlled Feeding Trials
Source: PLoS Med. 2016 Jul 19;13(7):e1002087. doi: 10.1371/journal.pmed.1002087 (PMC4951141; doi:10.1371/journal.pmed.1002087)
Supplement: S2 Fig — Assessment for small study bias in meta-regression using influence analysis, evaluating effects of isocaloric exchange of 5% energy between different macronutrients on (A) fasting glucose, (B) haemoglobin A1c, and (C) fasting insulin. (PDF) [file pmed.1002087.s002.pdf]

A. Fasting glucose

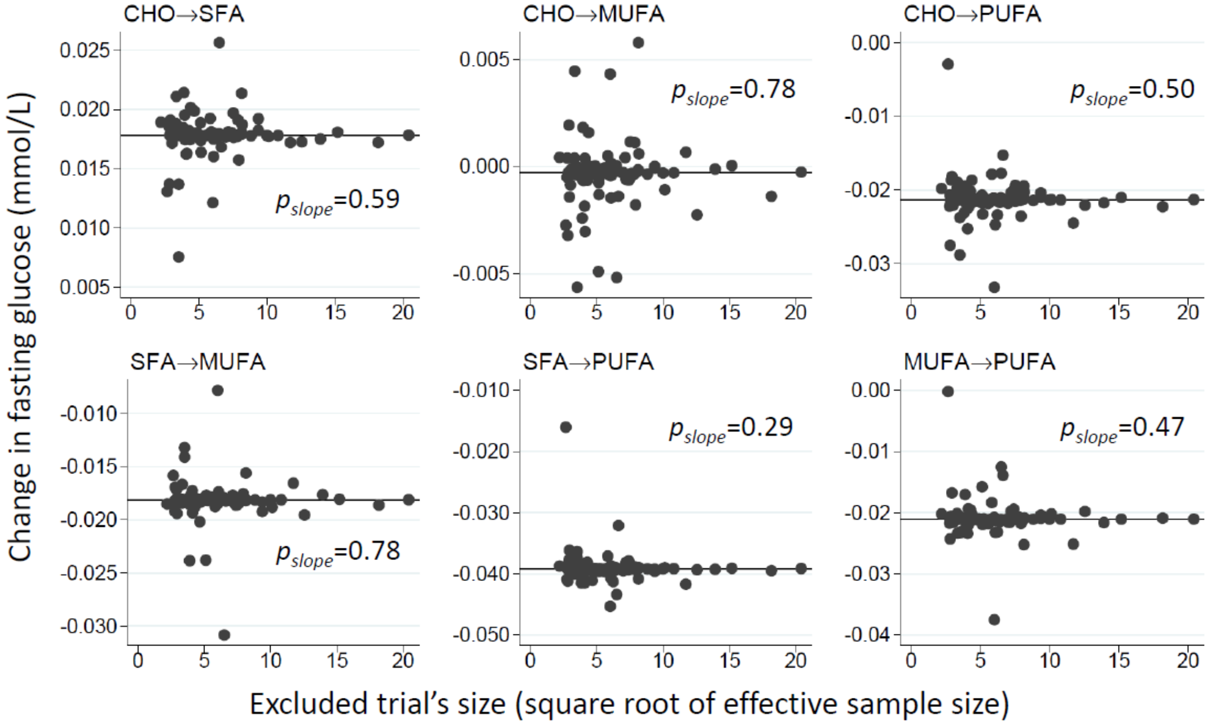

S2 Fig. Assessment for small study bias in meta-regression using influence analysis, evaluating effects of isocaloric exchange of 5% energy between different macronutrients on A) fasting glucose, B) haemoglobin A1c, and C) fasting insulin. 1 mg/dL=0.056 mmol/L for glucose, 1 μIU/mL=6 pmol/L, HbA1mmol/mol=(HbA1c % - 2.15)×10.929. For each comparison, the solid horizontal line represents the overall meta-regression estimate of effects; and each circle represents repeated meta-regression estimates of effects after excluding each single trial individually, plotted against the excluded trial's size (square root of effective sample size, accounting for within-trial correlations). Substantial asymmetry of the estimates excluding smaller trials (left-hand side) around the overall estimate would suggest potential publication bias. Linear regression of the points (analogous to Egger's test) and a test for symmetry did not identify any significant deviation from the null that would suggest publication bias (p>0.2 for any of the panels examined).

B. Haemoglobin A1c

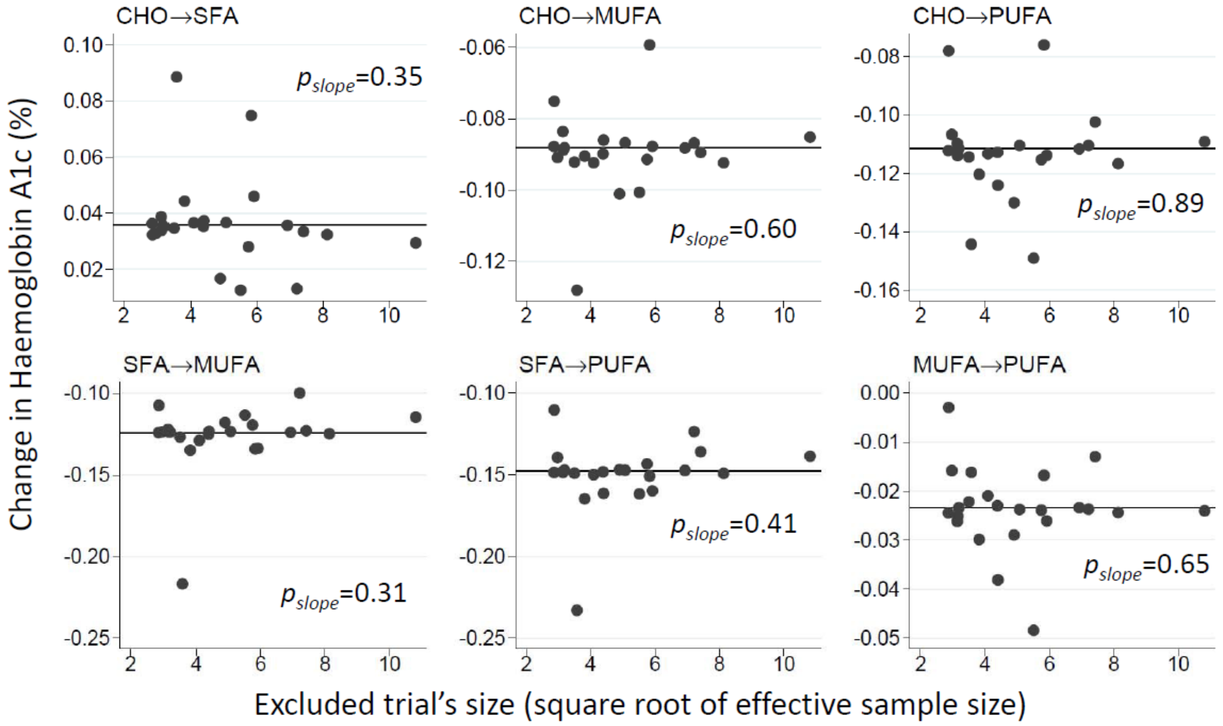

C. Fasting insulin

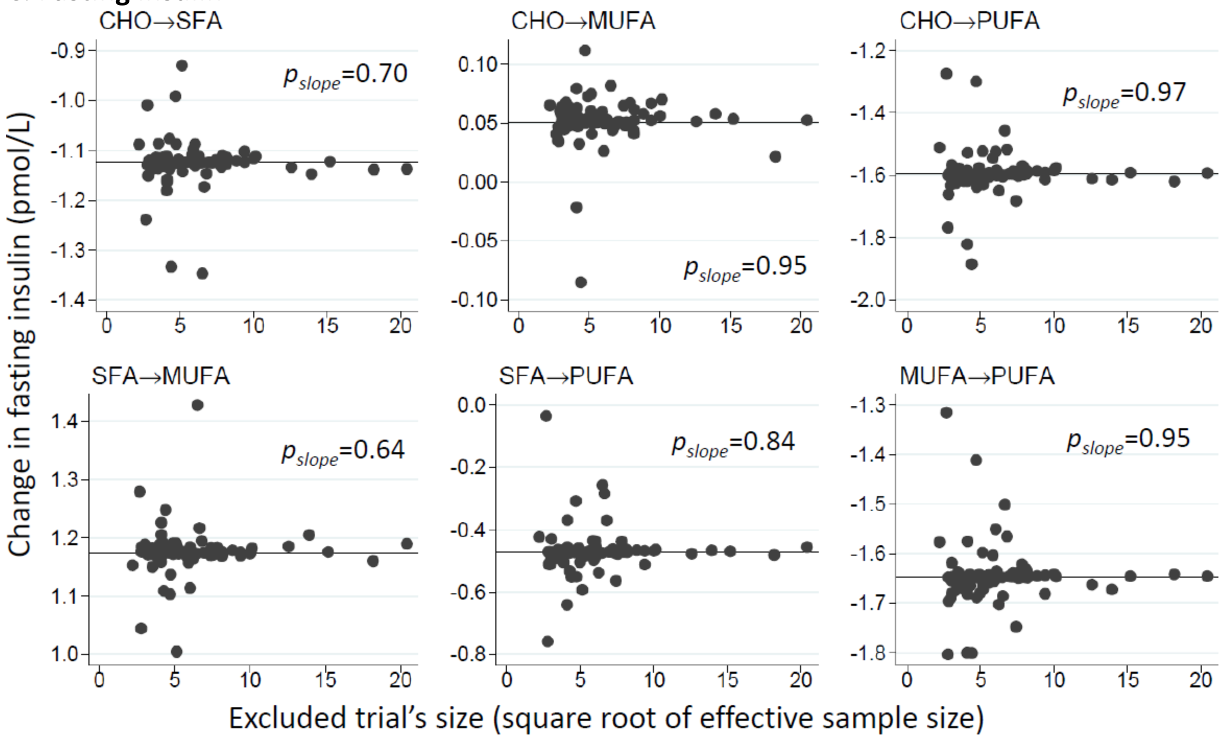

S2 Fig (continued).
