# Supplementary material for: Effects of Saturated Fat, Polyunsaturated Fat, Monounsaturated Fat, and Carbohydrate on Glucose-Insulin Homeostasis: A Systematic Review and Meta-analysis of Randomised Controlled Feeding Trials
Source: PLoS Med. 2016 Jul 19;13(7):e1002087. doi: 10.1371/journal.pmed.1002087 (PMC4951141; doi:10.1371/journal.pmed.1002087)
Supplement: S1 Table — (PDF) [file pmed.1002087.s003.pdf]

S1 Table. Characteristics of 102 randomized controlled feeding trials evaluated in the meta-analysis of effects of diets with different macronutrient compositions on glucose-insulin homeostasis.

Author, publication year, Location (Reference)	Design	N arms	Length days*	Dietary arms	N adults	Mean age (SD or range), years	Men, %	Mean BMI, kg/m^2	Glucose, mmol/L†	Macronutrient intake CHO/SFA/MUFA/PUFA % of energy‡	Measures §	Quality score ‖
**Trials of healthy adults**												
Coulston, 1983	Crossover	2	10	High CHO	11	45 (22-67)	45.5	24.9	5.1	60 / 5.8 / 8.3 / 6.9	Fasting	6.0
United States [56]				Low CHO	11	45 (22-67)	45.5	24.9	5.1	40 / 11 / 16 / 14		
Gustafsson, 1992	Parallel	2	21	MUFA	17	58.4 (52-72)	37.5	24.9	4.9	56 / 7.3 / 15 / 4.8	Fasting	7.0
Sweden [80]				PUFA	19	58.4 (40-65)	37.5	24.6	5.0	56 / 7.8 / 8.4 / 10		
Nydahl, 1994	Crossover	2	25	High MUFA	26	51.2 (23-74)	69.2	25.6	5.0	55 / 8 / 15 / 4	Fasting	7.0
Sweden [36]				High PUFA	26	51.2 (23-74)	69.2	25.6	5.0	56 / 8 / 8 / 11		
Uusitupa, 1994	Crossover	2	21	MUFA	7	23 (1.6)	0	21.5	4.5	44 / 8.5 / 19 / 10	Fasting	7.5
Finland [44]				SFA	7	23 (1.6)	0	21.5	4.5	45 / 20 / 12 / 3.9		
Nydahl, 1995	Crossover	2	25	Canola oil	22	54.2 (35-69)	54.5	25.3	5.2	56 / 7.8 / 14 / 8.9	Fasting	8.0
Sweden [112]				Olive oil	22	54.2 (35-69)	54.5	25.3	5.0	56 / 8.7 / 16 / 5.5		
Schwab, 1995	Crossover	2	28	Lauric acid diet	15	23.9 (4.6)	0	21.4	5.0	46 / 20 / 10 / 4.9	Fasting	8.5
Finland [68]				Palmitic acid diet	15	23.9 (4.6)	0	21.4	5.0	44 / 19 / 13 / 5.2	IV	
Espino-Montoro, 1996	Crossover	2	28	Olive oil	20	22.5 (19-26)	100	24.4	5.3	46 / 9.8 / 25 / 4.5	Fasting	7.5
Spain [117]				Sunflower oil	20	22.5 (19-26)	100	24.4	5.3	46 / 9 / 26 / 4.9		
Niskanen, 1997	Parallel	2	56	MUFA	14	55.4 (5.7)	64.3	29.1	6.4	43 / 11 / 19 / 8	Fasting	8.0
Finland [39]				PUFA	17	55.0 (5.3)	52.9	30.8	6.2	46 / 11 / 10 / 10	IV	
Jeppesen, 1997	Crossover	2	21	High CHO	10	66 (5.0)	0	27.0	4.9	40 / 14 / 17 / 14	Fasting	7.0
United States [42]				Low CHO	10	66 (5.0)	0	27.0	5.1	60 / 7.8 / 9.4 / 7.8		
Schwab, 1997	Crossover	2	21	PUFA	8	23 (21-25)	0	21.1	4.0	44 / 10 / 11 / 18	Fasting	8.5
Finland [43]				SFA	8	23 (21-25)	0	21.1	4.0	44 / 21 / 13 / 5		
Louheranta, 1998	Crossover	2	28	Oleic acid	15	22 (2.3)	0	22.6	4.7	42 / 13 / 19 / 6.4	Fasting	7.0
Finland [66]				Stearic acid	15	22 (2.3)	0	22.6	4.7	45 / 19 / 12 / 6.4	IV	
Lovejoy, 1998	Crossover	2	21	High fat, AA	20	36.6 (1.4)	100	31.9	5.2	35 / 20 / 19 / 11	Fasting	7.5
United States [22]**				Low fat, AA	20	36.6 (1.4)	100	31.9	5.2	55 / 7.4 / 8.4 / 4.2	IV	
	Crossover	2	21	High fat, White	11	34.4 (1.8)	100	30.2	5.2	35 / 20 / 19 / 11	Fasting	7.5
				Low fat, White	11	34.4 (1.8)	100	30.2	5.2	55 / 7.4 / 8.4 / 4.2	IV	
Louheranta, 1999	Crossover	2	28	MUFA	14	23 (3.0)	0	20.8	5.0	50 / 13 / 19 / 5.8	Fasting	8.5
Finland [70]				Trans fat	14	23 (3.0)	0	20.8	5.0	50 / 11 / 19 / 6.5	IV	
Thomsen, 1999	Crossover	2	28	CHO	16	35 (2.0)	37.5	25.8	4.2	53 / 8.8 / 8.1 / 6.7	Fasting	10.0
Denmark [72]				MUFA	16	35 (2.0)	37.5	25.8	4.2	41 / 9.4 / 24 / 5.5	IV	
Louheranta, 2000	Crossover	2	28	Low fat	18	53 (8.5)	72.2	29.6	6.1	47 / 8.4 / 18 / 7.8	Fasting	7.5
Finland [69]				MUFA	18	53 (8.5)	72.2	29.6	6.1	43 / 10 / 22 / 6.9	IV	
Matthan, 2001	Latino	6	35	Butter	18	60 (7.0)	100	28.1	5.3	54 / 17 / 8.1 / 2.4	Fasting	9.0
United States [21]**	Square			PHSO	18	60 (7.0)	100	28.1	5.1	56 / 7.3 / 8.1 / 12		
				Semiliquid margarine	18	60 (7.0)	100	28.1	5.2	52 / 8.6 / 8.1 / 14		
				Shortening	18	60 (7.0)	100	28.1	5.3	53 / 8.6 / 9.9 / 8.1		
				Soft margarine	18	60 (7.0)	100	28.1	5.1	53 / 8.4 / 8 / 11		
				Stick margarine	18	60 (7.0)	100	28.1	5.2	54 / 8.5 / 8.5 / 6.3		
	Latino	6	35	Butter	18	67 (4.0)	0	26.5	5.2	54 / 17 / 8.1 / 2.4	Fasting	9.0
	Square			PHSO	18	67 (4.0)	0	26.5	5.1	56 / 7.3 / 8.1 / 12		

S1 Table. Characteristics of 102 randomized controlled feeding trials evaluated in the meta-analysis of effects of diets with different macronutrient compositions on glucose-insulin homeostasis.

Author, publication year, Location (Reference)	Design	N arms	Length days*	Dietary arms	N adults	Mean age (SD or range), years	Men, %	Mean BMI, kg/m^2	Glucose, mmol/L†	Macronutrient intake CHO/SFA/MUFA/PUFA % of energy‡	Measures §	Quality score ‖
				Semiliquid margarine	18	67 (4.0)	0	26.5	5.1	52 / 8.6 / 8.1 / 14		
				Shortening	18	67 (4.0)	0	26.5	5.2	53 / 8.6 / 9.9 / 8.1		
				Soft margarine	18	67 (4.0)	0	26.5	5.0	53 / 8.4 / 8 / 11		
				Stick margarine	18	67 (4.0)	0	26.5	5.1	54 / 8.5 / 8.5 / 6.3		
Kriketos, 2001	Parallel	3	70	Omega-3 PUFA	16	46.9	37.5	33.4	5.6	50 / 9.3 / 23 / 9.3	Fasting	6.5
United States [53]				Omega-6 PUFA	17	47.0	35.3	33.5	5.5	50 / 9.3 / 23 / 9.3	IV	
				SFA	19	47.0	31.6	33.5	6.1	50 / 12 / 29 / 3		
Vidon, 2001	Crossover	2	21	High fat	7	40 (22-48)	42.9	20-27	4.8	41 / 14 / 15 / 14	Fasting	6.5
France [31]				High CHO	7	40 (22-48)	42.9	20-27	4.3	55 / 10 / 10 / 11	OTT	
Perez-Jimenez, 2001	Crossover	2	28	High CHO	59	23.1 (1.8)	50.8	22.9	4.9	55 / 9.2 / 14 / 5.2	Fasting	7.0
Spain [118]				Mediterranean	59	23.1 (1.8)	50.8	22.9	4.9	44 / 9.2 / 24 / 4.8		
Poppitt, 2002	Crossover	2	21	Control butter	20	23 (4.2)	100	21.7	4.7	47 / 20 / 6 / 14	Fasting	8.0
New Zealand [29]				Modified butter	20	23 (4.2)	100	21.8	4.7	48 / 15 / 8 / 16		
Kratz, 2002	Parallel	3	28	Olive oil	10	25.7 (5.4)	100	23.0	4.4	47 / 11 / 23 / 3.4	Fasting	8.0
Germany [23]**				Rapeseed oil	10	25.7 (5.4)	100	23.0	4.4	47 / 9.1 / 19 / 8.9		
				Sunflower oil	10	25.7 (5.4)	100	23.0	4.5	48 / 10 / 8.7 / 18		
	Parallel	3	28	Olive oil	9	25.7 (5.4)	0	23.0	4.1	47 / 11 / 23 / 3.4	Fasting	8.0
				Rapeseed oil	7	25.7 (5.4)	0	23.0	4.3	47 / 9.1 / 19 / 8.9		
				Sunflower oil	9	25.7 (5.4)	0	23.0	4.2	48 / 10 / 8.7 / 18		
Lovejoy, 2002	Crossover	3	28	MUFA	25	28 (10.0)	48.0	23.5	4.9	57 / 5.8 / 15 / 6.3	Fasting	9.0
United States [23]**				SFA	25	28 (10.0)	48.0	23.5	4.8	58 / 11 / 9.3 / 6.4	IV	
				Trans fat	25	28 (10.0)	48.0	23.5	4.7	58 / 7.3 / 8.4 / 4		
Clifton, 2004	Parallel	2	84	High MUFA	31	47.1 (10.7)	0	35.0	5.0	44 / 6.4 / 20 / 6.6	Fasting	8.0
Australia [119]				Very low fat	31	46.9 (9.9)	0	34.7	5.1	65 / 4.1 / 3.7 / 2.1		
Johnston, 2004	Parallel	2	42	High CHO, low fat	7	36.4 (4.2)	14.3	28.7	5.2	66 / 5.8 / 4.2 / 2.1	Fasting	7.5
United States [58]				High prot, low fat	9	40.1 (3.6)	11.1	29.1	5.3	40 / 7.9 / 11 / 5.4		
Hwalla, 2004	Parallel	2	28	High MUFA	7	28.6 (19-48)	100	34.8	5.3	35 / 10 / 30 / 5	Fasting	7.5
Lebanon [86]				High CHO	8	28.6 (19-48)	100	35.2	5.2	60 / 9 / 4 / 7		
Segal-Isaacson, 2004	Crossover	2	42	Low CHO	4	52.3 (3.8)	0	33.3	5.3	5 / 22 / 22 / 21	Fasting	9.0
United States [87]				Low fat	4	52.3 (3.8)	0	33.3	5.3	50 / 6 / 8 / 6		
Luscombe-Marsh, 2005	Parallel	2	112	High fat, standard prot	30	48.9 (23.2)	43.3	34.6	5.8	35 / 9.2 / 23 / 9.3	Fasting	8.5
Australia [77]				Low fat, high prot	27	51.7 (18.7)	44.4	33.4	5.6	35 / 9.3 / 14 / 3.8		
Noakes, 2005	Parallel	2	84	High prot	52	50 (10.0)	0	32.0	6.2	44 / 5.4 / 9.4 / 4.7	Fasting	8.0
Australia [78]				Low prot	48	49 (9.0)	0	33.0	6.1	61 / 4.6 / 8.3 / 4.7		
Keogh, 2005	Crossover	4	21	CHO	40	55.9 (10.7)	47.5	27.3	5.3†	65 / 7 / 6 / 3	Fasting	8.0
Australia [120]				MUFA	40	55.9 (10.7)	47.5	27.3	5.3	44 / 8 / 19 / 7		
				PUFA	40	55.9 (10.7)	47.5	27.3	5.4	45 / 9 / 10 / 15		
				SFA	40	55.9 (10.7)	47.5	27.3	5.9	45 / 19 / 12 / 4		
Johnston, 2006	Parallel	2	42	Ketogenic low CHO	9	38.4 (11.7)	22.2	35.0	5.5	9 / 21 / 20 / 8	Fasting	8.5
United States [120]				Non-ketogenic low CHO	10	37.2 (12.3)	20.0	34.6	5.2	42 / 8 / 10 / 4		
Tricon, 2006	Crossover	2	36	CLA-rich dairy	32	45.5 (8.7)	100	25.0	5.1	45 / 11 / 8.1 / 4.1	Fasting	9.0

S1 Table. Characteristics of 102 randomized controlled feeding trials evaluated in the meta-analysis of effects of diets with different macronutrient compositions on glucose-insulin homeostasis.

Author, publication year, Location (Reference)	Design	N arms	Length days*	Dietary arms	N adults	Mean age (SD or range), years	Men, %	Mean BMI, kg/m^2	Glucose, mmol/L†	Macronutrient intake CHO/SFA/MUFA/PUFA % of energy‡	Measures §	Quality score ‖
United Kingdom [37]				Normal dairy	32	45.5 (8.7)	100	25.0	5.1	45 / 13 / 9.1 / 5.2		
Schutte, 2006	Parallel	3	56	Cashew	21	45.7 (22.0)	38.1	34.7	4.8	44 / 9.3 / 16 / 7.8	Fasting	8.5
South Africa [121]				Control	21	44.4 (18.5)	47.6	35.5	4.9	47 / 12 / 11 / 9.5		
				Walnut	20	45.5 (22.2)	50.0	35.9	4.8	42 / 8.2 / 11 / 21		
Noakes, 2006	Parallel	3	84	High PUFA	21	46.1 (9.5)	25.0	33.4	5.4	48 / 5.6 / 12 / 7.5	Fasting	8.5
Australia [34]				Low CHO	24	48.4 (8.0)	16.7	32.5	5.3	10 / 18 / 27 / 6.4	OTT	
				Low fat	22	50.7 (10.3)	22.7	32.6	5.3	67 / 4.7 / 3.4 / 1.7		
Tholstrup, 2006	Parallel	2	35	Control	20	26.1 (3.6)	100	22.5	4.7	46 / 24 / 9.6 / 3.6	Fasting	8.0
Denmark [97]				Vaccenic acid diet	22	25.2 (3.9)	100	23.0	4.8	42 / 23 / 15 / 3.8		
Vega-Lopez, 2006	Crossover	2	35	Canola oil	15	63.9 (5.7)	33.3	26.0	4.9	53 / 6.4 / 15 / 8.7	Fasting	8.0
United States [107]				Palm oil	15	63.9 (5.7)	33.3	26.0	5.0	52 / 15 / 11 / 3.5		
Berglund, 2007	Crossover	3	49	Average American diet	85	35.5 (9.4)	61.2	27.6	5.2	49 / 16 / 14 / 5.8	Fasting	8.5
United States [105]				CHO diet	85	35.5 (9.4)	61.2	27.6	5.2	55 / 8 / 16 / 5.5		
				MUFA diet	85	35.5 (9.4)	61.2	27.6	5.2	49 / 8.7 / 21 / 6.2		
St-Onge, 2007	Crossover	3	25	High PUFA	33	43.6 (2.5)	21.2	29.4	5.2	49 / 8.5 / 15 / 9.7	Fasting	8.0
United States [38]				High SFA	33	43.6 (2.5)	21.2	29.4	5.2	46 / 11 / 16 / 5.8		
				High CHO	33	43.6 (2.5)	21.2	29.4	5.1	55 / 8.5 / 14 / 5.2		
Paniagua, 2007	Latino	3	28	CHO	10	62.3 (9.4)	36.4	32.6	5.5	65 / 6 / 8 / 6	Fasting	8.5
Spain [108]	Square			MUFA	11	62.3 (9.4)	36.4	32.6	5.5	47 / 9 / 23 / 6		
				SFA	11	62.3 (9.4)	36.4	32.6	5.5	47 / 23 / 9 / 6		
Sundram, 2007	Crossover	3	28	Esterified fat	30	30.0 (8.0)	34.4	22.0	5.4	53 / 18 / 5.9 / 7	Fasting	9.0
Malaysia [33]				PHSO	30	30.0 (8.0)	34.4	22.0	5.4	53 / 9.1 / 12 / 5.8		
				Palm oil	30	30.0 (8.0)	34.4	22.0	5.4	53 / 14 / 14 / 3.6		
Egert, 2008	Parallel	3	21	ALA	15	25.9 (6.8)	27.1	22.2	4.3	44 / 9.1 / 19 / 9.8	Fasting	8.0
Germany [106]				DHA	16	25.9 (6.8)	27.1	22.2	4.3	44 / 8.7 / 20 / 9.8		
				EPA	17	25.9 (6.8)	27.1	22.2	4.1	44 / 8.7 / 20 / 9.9		
Mensink, 2008	Crossover	2	21	Oleic acid, high trans-fat	44	41.0 (21.1)	25.0	23.9	5.5	44 / 12 / 19 / 6.2	Fasting	10.0
Netherlands [85]				Palmitic acid, low trans-fat	44	41.0 (21.1)	25.0	23.9	5.5	45 / 15 / 16 / 6.2		
Tay, 2008	Parallel	2	168	High CHO low fat	43	51.0 (7.5)	39.5	33.5	5.6	47 / 6 / 12 / 7	Fasting	8.0
Australia [122]				Low CHO high fat	45	50.3 (8.4)	31.1	33.9	5.7	5.1 / 21 / 25 / 8		
Vega-Lopez, 2009	Crossover	2	14	Corn oil	30	64.2 (7.5)	0	25.6	5.1	57 / 5.7 / 7.2 / 12	Fasting	7.5
United States [113]				PHSO	30	64.2 (7.5)	0	25.6	5.1	57 / 6.3 / 7.8 / 5.9		
Bradley, 2009	Parallel	2	56	Low CHO	12	37.1 (8.9)	37.5	34.5	5.5	20 / 21 / 21 / 13	Fasting	8.0
United Kingdom [62]				Low fat	12	40.5 (10.4)	37.5	32.8	5.6	60 / 7 / 6 / 3	OTT	
Tardy, 2009	Parallel	3	28	Dairy trans-fat	20	40 (18-50)	0	32.7	5.3	45 / 16 / 16 / 6	Fasting	9.5
France [30]				Industrial trans-fat	19	40 (18-50)	0	32.9	5.4	42 / 14 / 19 / 7.2		
				Low trans-fat	19	40 (18-50)	0	32.7	5.2	43 / 14 / 20 / 7.2		
Jebb, 2010	Parallel	2	168	High MUFA, low GI	100	51.4 (10.0)	42.0	28.5	5.4	45 / 9.6 / 16 / 6.9	Fasting	8.0
United Kingdom [27]**				Low fat, low GI	108	51.4 (10.0)	42.0	28.5	5.4	52 / 8.3 / 9.7 / 5.1	IV	
	Parallel	3	168	High MUFA, high GI	104	51.4 (10.0)	42.0	28.5	5.4	45 / 9.5 / 16 / 6.6	Fasting	8.0

S1 Table. Characteristics of 102 randomized controlled feeding trials evaluated in the meta-analysis of effects of diets with different macronutrient compositions on glucose-insulin homeostasis.

Author, publication year, Location (Reference)	Design	N arms	Length days*	Dietary arms	N adults	Mean age (SD or range), years	Men, %	Mean BMI, kg/m^2	Glucose, mmol/L†	Macronutrient intake CHO/SFA/MUFA/PUFA % of energy‡	Measures §	Quality score ‖
				High SFA, high GI	79	51.4 (10.0)	42.0	28.5	5.4	42 / 16 / 12 / 5.8	IV	
				Low fat, high GI	108	51.4 (10.0)	42.0	28.5	5.4	51 / 9.2 / 9.8 / 5.2		
Forsythe, 2010	Crossover	2	42	PUFA	8	45 (7.9)	100	30.0	6.0	12 / 17 / 25 / 15	Fasting	6.5
United States [114]				SFA	8	45 (7.9)	100	30.0	6.0	13 / 31 / 21 / 4.5		
Bos, 2010	Parallel	2	56	High MUFA	18	58.9 (5.1)	44.0	27.6	5.2	46 / 11 / 20 / 7	Fasting	9.5
Netherlands [123]				High SFA	19	52.0 (7.2)	42.0	26.1	5.4	47 / 19 / 11 / 5.4		
Yeung, 2010	Crossover	2	42	CHO	161	53.6 (10.9)	55.0	30.2	5.2	58 / 6 / 13 / 8	Fasting	8.0
United States [47]				Unsaturated fat	161	53.6 (10.9)	55.0	30.2	5.2	48 / 6 / 21 / 10		
Bendsen, 2011	Parallel	2	112	Control fat	25	58.8 (5.5)	0	28.1	5.3	44 / 12 / 14 / 4.3	Fasting	9.0
Denmark [91]				Trans fat	24	58.5 (4.6)	0	28.8	5.2	44 / 11 / 10 / 4.5		
Goree, 2011	Parallel	2	56	Reduced CHO, high fat	34	35.6 (8.5)	43.0	33.3	5.5	43 / 12 / 13 / 11	Fasting	7.0
United States [46]				Standard diet	29	34.6 (8.1)	48.0	31.3	5.4	56 / 8.3 / 9.8 / 7.8		
Hjerpsted, 2011	Crossover	2	42	Butter	49	55.5 (12.4)	57.0	25.3	5.6	48 / 16 / 11 / 4.6	Fasting	6.0
Denmark [98]				Cheese	49	55.5 (12.4)	57.0	25.3	5.6	45 / 15 / 10 / 4.4		
Iggman, 2011	Crossover	2	21	Rapeseed oil	20	50.9 (10.0)	70.0	26.3	5.5	49 / 7.8 / 16 / 8.7	Fasting	8.0
Sweden [40]				SFA or dairy FA	20	50.9 (10.0)	70.0	26.3	5.5	49 / 19 / 11 / 3.6		
Johnstone, 2011	Crossover	2	28	Low CHO	16	55 (14.0)	100	35.8	5.8	4.7 / 21 / 29 / 12	Fasting	7.0
Scotland [92]				Mod CHO	16	55 (14.0)	100	35.8	5.8	37 / 12 / 15 / 5.4		
Tholstrup, 2011	Crossover	3	21	Lard	32	29.6 (10.3)	100	22.9	5.3	49 / 15 / 15 / 5.6	Fasting	7.0
Denmark [124]				Olive oil	32	29.6 (10.3)	100	22.9	5.3	51 / 8.7 / 20 / 5.8		
				Palm olein	32	29.6 (10.3)	100	22.9	5.3	49 / 14 / 15 / 6.4		
Tierney, 2011	parallel	4	98	High MUFA	111	54.6 (8.7)	41.4	32.4	5.9	42 / 10 / 19 / 6.7	Fasting	8.5
8 European nations[125]				High SFA	100	54.9 (8.6)	45.0	31.8	6.0	41 / 18 / 13 / 6.3	IV	
				Low fat high CHO	106	54.7 (9.4)	44.3	32.5	5.9	49 / 8.6 / 12 / 6		
				Low fat high CHO n-3 fat	100	55.4 (9.6)	45.0	32.4	6.0	50 / 8.7 / 11 / 5.7		
Bjermo, 2012	Parallel	2	70	Linoleic acid	32	57 (51-63)	44.0	30.3	5.3	44 / 10 / 12 / 15	Fasting	10.0
Sweden [48]				SFA	29	56 (50-64)	44.0	31.3	5.4	43 / 21 / 14 / 4.6		
Guay, 2012	Crossover	2	3	High fat	12	27.1 (3.9)	100	25.2	5.0	50 / 15 / 13 / 4.3	Fasting	7.0
Canada [109]				Low fat	12	27.1 (3.9)	100	25.2	5.0	62 / 6 / 12 / 4.9		
Haghighatdoost, 2012	Crossover	2	42	MUFA-rich	17	34.8 (7.9)	0	27.6	4.4	52 / 8.5 / 13 / 7.5	Fasting	9.5
Iran [49]				SFA-rich diet	17	34.8 (7.9)	0	27.6	4.4	50 / 14 / 7 / 11		
Wycherley, 2012	Parallel		84	CHO diet	34	50.2 (9.3)	100	33.0	5.8	51 / 7.5 / 9.6 / 4.6	Fasting	9.0
Australia[126]				Protein diet	32	51.3 (9.4)	100	33.0	5.8	37 / 8.2 / 11 / 4.6		
Kien, 2013	Crossover	2	21	Oleic acid	9	28.9 (7.6)	100	23.4	4.6	44 / 3.7 / 29 / 6.6	Fasting	7.0
United States [24]**				Palmitic acid	9	28.9 (7.6)	100	23.4	4.6	43 / 18 / 16 / 5.1	IV	
	Crossover	2	21	Oleic acid	9	30 (6.6)	0	22.8	4.5	44 / 3.7 / 29 / 6.6	Fasting	7.0
				Palmitic acid	9	30 (6.6)	0	22.8	4.5	43 / 18 / 16 / 5.1	IV	
Young, 2013	Crossover	3	56	High fat	17	57 (6.0)	0	28.0	6.0	45 / 28 / 29 / 28	Fasting	8.5
United States [94]				Low fat	16	57 (6.0)	0	28.0	6.0	65 / 13 / 14 / 13		
				Low fat, n-3 PUFA	16	57 (6.0)	0	28.0	5.8	62 / 15 / 16 / 16		
Chiu, 2014	Parallel	5	28	Control diet	31	38 (12.0)	25.8	33.9	5.2	55 / 7.3 / 13 / 7.2	Fasting	8.5

S1 Table. Characteristics of 102 randomized controlled feeding trials evaluated in the meta-analysis of effects of diets with different macronutrient compositions on glucose-insulin homeostasis.

Author, publication year, Location (Reference)	Design	N arms	Length days*	Dietary arms	N adults	Mean age (SD or range), years	Men, %	Mean BMI, kg/m^2	Glucose, mmol/L†	Macronutrient intake CHO/SFA/MUFA/PUFA % of energy‡	Measures §	Quality score ‖
United States [50]				High protein, high SFA	32	38 (12.0)	25.0	33.9	5.2	35 / 15 / 9.8 / 7.1	IV	
				High protein, low SFA	36	38 (12.0)	44.4	33.9	5.2	35 / 7.4 / 18 / 7.6		
				Mod protein, high SFA	29	38 (12.0)	17.2	33.9	5.2	35 / 15 / 20 / 7.1		
				Mod protein, low SFA	30	38 (12.0)	33.3	33.9	5.2	35 / 7.5 / 29 / 7.4		
Filippou, 2014	Crossover	3	42	High oleic sunflower oil	41	29.1 (7.6)	24.4	23.0	4.9	55 / 4.4 / 17 / 4.1	Fasting	9.5
Malaysia [100]				Esterified palm olein	41	29.1 (7.6)	24.4	23.0	4.9	55 / 11 / 10 / 3.9	OTT	
				Palm olein	41	29.1 (7.6)	24.4	23.0	4.9	55 / 11 / 11 / 4.1		
Iggman, 2014	Parallel	2	49	PUFA	19	26.5 (4.6)	68.0	20.8	4.6	43 / 12 / 12 / 13	Fasting	10.5
Sweden [51]				SFA	20	27.4 (3.8)	70.0	19.7	4.5	48 / 16 / 13 / 4.5		
Jordy, 2014	Parallel	2	3	High CHO	7	25.4 (3.2)	100	24.5	5.1	80 / 2.8 / 2.3 / 5.9	Fasting	4.0
Denmark [127]				High fat	11	23.8 (3.0)	100	22.8	5.0	10 / 12 / 47 / 18		
Marina, 2014	Crossover	2	28	High fat	7	36 (2.9)	76.9	33.6	5.3	47 / 12 / 17 / 4.7	Fasting	7.5
United States [128]				Low fat	7	36 (2.9)	76.9	33.6	5.3	62 / 7.7 / 7.7 / 3		
Rietman, 2014	Crossover	2	14	High protein	9	22.8 (0.7)	19.0	21.5	5.2	37 / 12 / 12 / 12	Fasting	7.5
Netherlands [129]				Normal protein	8	22.8 (0.7)	19.0	21.5	5.2	45 / 12 / 14 / 12		
Sacks, 2014	Crossover	2	35	High CHO, low GI	153	53 (11.0)	48.0	32.0	5.4	57 / 6 / 13 / 8	Fasting	10.0
United States [26]**				Low CHO, low GI	153	53 (11.0)	48.0	32.0	5.4	40 / 7 / 19 / 10	OTT	
	Crossover	2	35	High CHO, high GI	150	53 (11.0)	48.0	32.0	5.4	58 / 6 / 12 / 7	Fasting	10.0
				Low CHO, high GI	151	53 (11.0)	48.0	32.0	5.4	41 / 7 / 18 / 10	OTT	
Rozati, 2015	Parallel	2	90	Control diet	21	72 (4.6)	29.0	29.0	5.7	43 / 13 / 13 / 13	Fasting	8.5
United States [101]				Olive oil	20	72 (4.5)	40.0	29.0	5.6	43 / 11 / 20 / 6.6		
Vafeiadou, 2015	Parallel	3	112	MUFA	64	43 (8.0)	42.2	26.3	5.0	49 / 8.1 / 19 / 5.7	Fasting	9.0
United Kingdom [96]				PUFA	65	45 (8.1)	43.9	27.0	5.0	49 / 8 / 12 / 11		
				SFA	65	45 (8.1)	44.6	26.7	5.1	48 / 18 / 11 / 3.9		
Baril-Gravel, 2015	Crossover	5	28	Canola + n-3 PUFA	130	46.5 (14.2)	46.2	29.8	5.4	50 / 6.0 / 10 / 16	Fasting	10.0
Canada [52]				Corn + safflower oil	130	46.5 (14.2)	46.2	29.8	5.4	50 / 6.8 / 9.6 / 16		
				Canola	130	46.5 (14.2)	46.2	29.8	5.4	50 / 6.6 / 18 / 9.1		
				Flax + safflower oil	130	46.5 (14.2)	46.2	29.8	5.4	50 / 6.5 / 19 / 6.9		
				Canola + oleic acid	130	46.5 (14.2)	46.2	29.8	5.4	50 / 6.9 / 18 / 8.0		
**Trials of adults with diabetes**												
Garg, 1988	Crossover	2	28	High MUFA	10	56 (6.3)	100	29.0	7.2	35 / 10 / 33 / 7	Fasting	7.5
United States [130]				High CHO	10	56 (6.3)	100	29.0	7.2	60 / 9 / 9 / 6		
Coulston, 1989	Crossover	2	42	High CHO	8	66 (50-73)	62.5	25.5	10.5	40 / 12 / 17 / 12	Fasting	6.0
United States [55]				Low fat	8	66 (50-73)	62.5	25.5	10.5	60 / 5.7 / 8.1 / 6.2		
Fuh, 1990	Crossover	2	15	High CHO	11	58 (49-64)	100	25.8	7.9	60 / 5.2 / 7.5 / 7.3	Fasting	6.0
Taiwan [57]				High fat	11	58 (49-64)	100	25.8	7.6	40 / 10 / 15 / 15		
Garg, 1992	Crossover	2	21	High CHO	8	63 (6.0)	100	30.0	6.4	60 / 8 / 12 / 5	Fasting	6.5
United States [61]				Low CHO	8	63 (6.0)	100	30.0	6.1	35 / 11 / 32 / 7	OTT	
Vessby, 1992	Crossover	2	21	PUFA	14	63 (39-86)	33.3	26.1	11.9	56 / 8.5 / 10 / 12	Fasting	5.5
Sweden [84]				SFA	14	63 (39-86)	33.3	26.1	11.9	56 / 16 / 10 / 5.1		

S1 Table. Characteristics of 102 randomized controlled feeding trials evaluated in the meta-analysis of effects of diets with different macronutrient compositions on glucose-insulin homeostasis.

Author, publication year, Location (Reference)	Design	N arms	Length days*	Dietary arms	N adults	Mean age (SD or range), years	Men, %	Mean BMI, kg/m^2	Glucose, mmol/L†	Macronutrient intake CHO/SFA/MUFA/PUFA % of energy‡	Measures §	Quality score ||
Garg, 1992	Crossover	2	28	High MUFA	10	61.5 (4.0)	100	27.7	7.2	38 / 5 / 31 / 10	Fasting	8.5
United States [131]				High CHO	10	61.5 (4.0)	100	27.7	6.6	65 / 3 / 11 / 6		
Parillo, 1992	Crossover	2	15	High MUFA, low CHO	10	52.7 (8.4)	70.0	26.7	6.6	40 / 7 / 29 / 4	Fasting	7.5
Italy [89]				Low MUFA, high CHO	10	52.7 (8.4)	70.0	26.7	6.6	60 / 5 / 13 / 2		
Rasmussen, 1993	Crossover	2	21	High MUFA	15	57 (7.7)	66.7	27.0	8.4	36 / 10 / 30 / 7	Fasting	7.5
Denmark [67]				High CHO	15	57 (7.7)	66.7	27.0	8.4	49 / 11 / 11 / 7	OTT	
Lerman-Garber, 1994	Crossover	2	28	High MUFA	14	56 (8.0)	0	28.0	6.5	40 / 11 / 24 / 5	Fasting	5.0
Mexico [102]				High CHO	14	56 (8.0)	0	28.0	7.2	60 / 6.6 / 6.6 / 6.6		
Garg, 1994	Crossover	2	42	High MUFA	42	58 (10.0)	78.6	28.1	8.3	40 / 10 / 25 / 10	Fasting	6.5
United States [88]				High CHO	42	58 (10.0)	78.6	28.1	8.3	55 / 10 / 10 / 10		
Walker, 1995	Crossover	2	90	High CHO, low fat	24	58.3 (2.1)	37.5	29.2	8.5	50 / 9 / 10 / 4	Fasting	5.5
Australia [82]				Modified fat	24	58.3 (2.1)	37.5	29.2	9.6	40 / 11 / 20 / 5		
Lerman-Garber, 1995	Crossover	2	42	High MUFA	8	60 (7.0)	0	25.2	11.7	40 / 11 / 24 / 5	Fasting	7.0
Mexico [83]				High CHO	5	60 (7.0)	0	25.2	12.4	60 / 6.6 / 6.6 / 6.6		
Thomsen, 1995	Crossover	2	21	MUFA	16	59 (2.0)	62.5	28.0	8.2	37 / 10 / 30 / 7	Fasting	7.0
Denmark [63]				PUFA	16	59 (2.0)	62.5	28.0	8.4	36 / 9 / 10 / 27		
Katsilambros, 1996	Crossover	2	120	Olive oil	7	54.5 (7.4)	84.6	28.2	8.7	45 / 11 / 20 / 4	Fasting	6.5
Greece [104]				Sunflower oil	6	54.5 (7.4)	84.6	28.2	9.1	45 / 11 / 8 / 16		
Christiansen, 1997	Crossover	3	42	Cis-MUFA	16	55 (12.0)	56.3	32.8	9.0	50 / 5 / 20 / 5	Fasting	6.5
Denmark [115]				SFA	16	55 (12.0)	56.3	32.5	9.0	50 / 20 / 2.5 / 5		
				Trans-MUFA	16	55 (12.0)	56.3	32.7	9.0	50 / 5 / 0 / 5		
Storm, 1997	Crossover	3	21	CHO	15	53 (9.0)	53.3	29.7	7.7	51 / 9 / 11 / 5	Fasting	7.0
Denmark [132]				Palmitic acid	15	53 (9.0)	53.3	29.7	7.8	40 / 21 / 16 / 6		
				Stearic acid	15	53 (9.0)	53.3	29.7	7.9	40 / 19 / 17 / 6		
Georgopoulos, 1998	Crossover	2	28	High MUFA	17	32.2 (5.4)	52.9	23.5	8.4	45 / 9 / 25 / 6	Fasting	5.5
United States [35]				High CHO	17	32.2 (5.4)	52.9	23.5	9.3	61 / 9 / 9 / 6		
Brynes, 2000	Crossover	2	21	High MUFA	9	56 (5.3)	55.6	29.8	8.0	47 / 8 / 20 / 4.2	Fasting	7.5
United Kingdom [60]				High PUFA	9	56 (5.3)	55.6	29.8	7.9	47 / 9.2 / 12 / 13	OTT	
Shige, 2000	Parallel	3	84	High MUFA	13	58.7 (2.5)	15.4	33.6	8.1	50 / 6.8 / 15 / 9	Fasting	7.5
Australia [65]				High SFA	10	58.9 (3.0)	30.0	33.3	7.4	52 / 17 / 9.9 / 2.1	OTT	
				High CHO	12	57.5 (3.4)	25.0	32.6	8.6	73 / 3.5 / 3 / 2.1		
Higashi, 2001	Crossover	2	21	Linoleic acid	8	62 (6.9)	25.0	24.7	9.4	58 / 5 / 5 / 15	Fasting	6.0
Japan [103]				Oleic acid	8	62 (6.9)	25.0	24.7	9.4	58 / 5 / 15 / 5		
Lovejoy, 2002	Crossover	2	28	High fat, almond	30	53.8 (10.4)	43.3	33.0	8.1	46 / 6.8 / 23 / 11	Fasting	8.5
United States [25]**				Low fat, almond	30	53.8 (10.4)	43.3	33.0	8.7	58 / 4.8 / 16 / 7.7	OTT	
	Crossover	2	28	High fat	30	53.8 (10.4)	43.3	33.0	8.6	49 / 7.4 / 21 / 8.7	Fasting	8.5
				Low fat	30	53.8 (10.4)	43.3	33.0	8.0	60 / 5.3 / 15 / 6.5	OTT	
Rodriguez-Villar, 2003	Crossover	2	14	CHO diet	22	61 (7.0)	54.5	28.3	9.0	52 / 6.4 / 14 / 4.3	Fasting	8.0
Spain [90]				MUFA diet	22	61 (7.0)	54.5	28.3	9.0	41 / 7.3 / 25 / 5		
Gannon, 2004	Crossover	2	35	Control	8	63.3 (51-82)	100	31.0	10.0	55 / 11 / 9.3 / 7.7	Fasting	7.5
United States [41]				LoBAG	8	63.3 (51-82)	100	31.0	9.3	20 / 9.6 / 20 / 11		

S1 Table. Characteristics of 102 randomized controlled feeding trials evaluated in the meta-analysis of effects of diets with different macronutrient compositions on glucose-insulin homeostasis.

Author, publication year, Location (Reference)	Design	N arms	Length days*	Dietary arms	N adults	Mean age (SD or range), years	Men, %	Mean BMI, kg/m^2	Glucose, mmol/L†	Macronutrient intake CHO/SFA/MUFA/PUFA % of energy‡	Measures §	Quality score ||
Miyashita, 2004	Parallel	2	28	High CHO	11	52.4 (13.0)	72.7	27.0	11.1	62 / 3 / 4 / 3	Fasting	4.0
Japan [133]				Low CHO	11	52.4 (13.0)	72.7	27.0	11.5	39 / 11 / 14 / 11		
Papakonstantinou, 2010	Crossover	2	28	High prot low fat	17	46 (3.0)	29.4	33.0	7.1	50 / 6.3 / 9.1 / 3.4	Fasting	10.0
Greece [134]				Low prot high fat	17	46 (3.0)	29.4	34.0	7.5	50 / 6.7 / 21 / 5	OTT	
Taylor, 2010	Parallel	3	84	Control diet	9	52.4 (1.5)	50.0	32.4	7.7	45 / 14 / 15 / 5.3	Fasting	8.0
Canada [135]				Flaxseed oil	12	52.4 (1.5)	50.0	32.4	6.9	46 / 11 / 12 / 7.1		
				Milled flaxseed	13	52.4 (1.5)	50.0	32.4	6.6	48 / 12 / 13 / 8.7		
Gannon, 2011	Crossover	2	35	Control	8	61 (5.9)	100	31.0	8.2	55 / 10 / 10 / 10	Fasting	6.5
United States [45]				LoBAG	8	61 (5.9)	100	31.0	8.4	29 / 11 / 17 / 9		
Jenkins, 2011	Parallel	3	90	Half dose	38	62 (8.0)	68.0	30.0	7.4	41 / 10 / 16 / 9.3	Fasting	9.5
Canada [136]				Muffins	39	61 (10.0)	67.0	29.0	7.1	44 / 11 / 11 / 9.2		
				Nuts	40	63 (9.0)	65.0	29.0	7.3	39 / 9.4 / 19 / 9.2		
Li, 2011	Crossover	2	28	Almond	20	58 (2.0)	45.0	26.0	8.7	47 / 6.1 / 17 / 11	Fasting	8.0
Taiwan [93]				Control	20	58 (2.0)	45.0	26.0	8.7	57 / 6.1 / 8.1 / 9.4		
Helge, 2012	Crossover	2	21	CHO rich	11	58.7 (7.0)	100	28.1	7.5	59 / 28 / 21 / 9.5	Fasting	6.5
Denmark [99]				Fat rich	11	58.7 (7.0)	100	28.1	7.5	22 / 25 / 19 / 15		
Tay, 2015	Parallel	2	84	High CHO	37	58 (7.0)	51.0	35.1	8.4	51 / 7.2 / 11 / 4.1	Fasting	10.0
Australia [95]				Low CHO	41	58 (7.0)	64.0	34.2	7.8	13 / 9.8 / 31 / 12		

The reference list is in S3 Text. Abbreviations: BMI, body mass index; CHO, carbohydrates; DHA, docosahexaenoic acid; EPA, eicosapentaenoic acid; GI, glycaemic index; LoBAG, Low biologically available glucose diet; MUFA, monounsaturated fatty acids; PHSO, partially hydrogenated soybean oil; OTT, oral tolerance test; PUFA, polyunsaturated fatty acids; SFA, saturated fatty acids.

* Duration of an intervention.

† Baseline measures of fasting glucose. Keogh et al (2005) did not report baseline glucose values [120] and was imputed. The uncertain of imputation was accounted for by undertaking multiple imputation using ten imputed datasets (S3 Text).

‡ All trials performed interventions of meals with caloric contents similar across arms (isocaloric intervention). Protein consumption (% energy) can be approximated by calculating 100 minus % energy from CHO, SFA, MUFA, plus PUFA.

§ Outcome measures varied across trials. Fasting indicates availability of any of the fasting blood measures (fasting glucose, fasting insulin, haemoglobin A1c, C-peptide, and homeostasis model assessment for insulin resistance); OTT, 2-hour glucose and 2-hour insulin from oral-glucose or oral-meal tolerance test; and IV, insulin sensitivity index or acute insulin response derived from IV assays.

|| The quality score was based on 11-point Jadad Scale [137]. Eleven components reflect different aspects of reporting quality: study objectives, inclusion/exclusion criteria, control definition, sample size/power calculation, randomisation, blinding, drop-out, outcome definition, exposure definition, assessment of adverse events, and statistical methods. Two reviewers independently assigned scores of these eleven components to each trial, average scores for the components were calculated and summed up (Table S2 for details). Inter-rater correlation for the total score was 0.61, consistent with that observed by Jadad et al. The most common reasons for lower scores were absence of information on adverse outcomes (% reported=13%); justification of sample size (22.6%); and blinding (34.5%).

** The publication reported estimates stratified by demographics (sex or race) or estimates which included non-comparable arms (e.g. low fat + high GI and high fat + low GI by Sacks et al. 2014 [26]). The number of trial was counted as one, but only comparable arms were pooled in meta-analysis (e.g. comparison between low fat + high GI and high fat + high GI and comparison between low fat + low GI and high fat + low GI). Lovejoy et al. [23] reported results for intravenous measures stratified by race (African American and White) and non-stratified results for fasting glucose and fasting insulin.
