# Supplementary material for: Effects of Saturated Fat, Polyunsaturated Fat, Monounsaturated Fat, and Carbohydrate on Glucose-Insulin Homeostasis: A Systematic Review and Meta-analysis of Randomised Controlled Feeding Trials
Source: PLoS Med. 2016 Jul 19;13(7):e1002087. doi: 10.1371/journal.pmed.1002087 (PMC4951141; doi:10.1371/journal.pmed.1002087)
Supplement: S2 Table — (PDF) [file pmed.1002087.s004.pdf]

S2 Table. Characteristics and scores of reporting quality of 102 trials eligible for the meta-analysis of randomized controlled feeding trials of macronutrient intakes and glycaemic outcomes.

								Assessment of quality ‖										
Author of trial, year	Ward	Weight loss (caloric restriction)	Blinding intervention /outcome measures*	Sources of food composition †	Compliance assessment ‡	Food provided, % of energy or text §	Drop out %	Randomisation	Blinding	Drop out	Study objectives	Outcome definition	Eligibility criteria	Sample size	Exposure definition	Control arm(s)	Adverse effects	Statistical methods
**Trials recruiting adults without diabetes**																		
Coulston, 1983 [56]	yes	no	no/no	Database	N/A	100	N/A	1.0	0.0	0.0	1.0	1.0	0.0	0.0	1.0	1.0	0.0	1.0
Gustafsson, 1992 [80]	yes	no	no/no	Fat	CE FA	100	10.0	1.0	0.0	0.5	1.0	0.5	1.0	0.0	1.0	1.0	0.0	1.0
Nydahl, 1994 [36]	no	no	no/no	Fat	Leftover, CE FA	100	N/A	0.0	0.5	0.5	1.0	1.0	1.0	0.0	1.0	1.0	0.0	1.0
Uusitupa, 1994 [44]	no	no	yes/yes	Database	7d DR, TG FA	Fat products, veg oils	30.0	1.0	0.5	0.5	1.0	1.0	0.5	0.0	1.0	1.0	0.0	1.0
Schwab, 1995 [68]	no	no	no/no	Database	7d DR, TG FA	Key fat sources	N/A	1.0	1.0	1.0	1.0	1.0	0.5	0.0	1.0	1.0	0.0	1.0
Nydahl, 1995 [112]	no	no	no/no	Fat	N/A	100	0	1.0	0.0	1.0	1.0	1.0	1.0	0.0	1.0	1.0	0.0	1.0
EspinoMontoro, 1996 [117]	no	no	no/no	Meal	Counselling, Leftover	100	N/A	1.0	1.0	0.0	1.0	1.0	0.5	0.0	1.0	1.0	0.0	1.0
Niskanen, 1997 [39]	no	no	no/no	Database	4d DR, TG FA	Fat, salad dressings	N/A	1.0	0.5	0.5	1.0	1.0	1.0	0.0	1.0	1.0	0.0	1.0
Jeppesen, 1997 [42]	yes	no	no/no	Database	Counselling, Leftover	100	0	1.0	0.5	0.5	1.0	1.0	0.0	0.0	1.0	1.0	0.0	1.0
Schwab, 1997 [43]	no	no	no/no	Database	TG FA, CE FA, DR	Butter, margarine, vegetable oils	6.3	1.0	1.0	1.0	1.0	1.0	0.5	0.0	1.0	1.0	0.0	1.0
Louheranta, 1998 [66]	no	no	no/no	Database	7d DR, TG FA, CE FA	Fat products, vegetable oils, and dairy products	N/A	1.0	1.0	0.0	1.0	1.0	0.0	0.0	1.0	1.0	0.0	1.0
Lovejoy, 1998 [22]	no	no	no/no	Database	Leftover	100	N/A	1.0	1.0	0.0	1.0	1.0	0.5	0.0	1.0	1.0	0.0	1.0
Thomsen, 1999 [72]	no	no	no/no	Fat	TG FA	70	0	1.0	1.0	1.0	1.0	1.0	0.5	1.0	1.0	1.0	0.5	1.0
Louheranta, 1999 [70]	no	no	no/no	Fat	7d DR, TG FA, CE FA	Fat products, vegetable oils, and dairy products	6.7	1.0	1.0	1.0	1.0	1.0	0.5	0.0	1.0	1.0	0.0	1.0
Louheranta, 2000 [69]	no	no	no/no	Fat	7d DR, TG FA, CE FA	Fat products, vegetable oils	N/A	1.0	0.5	0.0	1.0	1.0	1.0	0.0	1.0	1.0	0.0	1.0
Matthan, 2001 [21]	no	no	yes/yes	Meal	NR	100	22.2	1.0	1.0	1.0	1.0	1.0	1.0	0.0	1.0	1.0	0.0	1.0
Kriketos, 2001 [53]	no	yes	no/no	Database	daily DR, Counselling	100	N/A	1.0	0.0	0.0	1.0	1.0	1.0	0.0	0.0	1.0	0.5	1.0
Vidon, 2001 [31]	no	no	no/no	Database	7d DR	100	N/A	1.0	0.5	0.0	1.0	1.0	0.0	0.0	1.0	1.0	0.0	1.0
Perez-Jimenez, 2001 [118]	no	no	no/no	Meal	FD, CE FA	100	N/A	1.0	0.0	0.0	1.0	1.0	0.5	0.0	1.0	1.0	0.5	1.0
Lovejoy, 2002 [23]	no	no	no/no	Meal	Serum FA	100	19.4	1.0	1.0	0.5	1.0	1.0	1.0	0.5	1.0	1.0	0.0	1.0
Poppitt, 2002 [29]	yes	no	no/yes	Meal	Urine N	100	N/A	1.0	1.0	0.5	1.0	1.0	0.5	0.0	1.0	1.0	0.0	1.0
Kratz, 2002 [23]	no	no	no/no	Database	FD	90	15.9	1.0	0.0	1.0	1.0	1.0	1.0	0.0	1.0	1.0	0.0	1.0
Segal-Isaacson, 2004 [87]	no	yes	no/no	Meal	Leftover, Counselling	100	0	1.0	0.0	0.5	1.0	1.0	1.0	1.0	1.0	1.0	1.0	0.5

S2 Table. Characteristics and scores of reporting quality of 102 trials eligible for the meta-analysis of randomized controlled feeding trials of macronutrient intakes and glycaemic outcomes.

								Assessment of quality ‖										
Author of trial, year	Ward	Weight loss (caloric restriction)	Blinding intervention /outcome measures*	Sources of food composition †	Compliance assessment ‡	Food provided, % of energy or text §	Drop out %	Randomisation	Blinding	Drop out	Study objectives	Outcome definition	Eligibility criteria	Sample size	Exposure definition	Control arm(s)	Adverse effects	Statistical methods
Hwalla, 2004 [86]	no	yes	no/no	Database	DR, Counselling	100	6.3	1.0	0.0	0.5	1.0	1.0	1.0	0.0	1.0	1.0	0.0	1.0
Johnston, 2004 [58]	no	yes	no/no	Database	Counselling	100	20.0	1.0	0.0	1.0	1.0	1.0	0.5	0.0	1.0	1.0	0.0	1.0
Clifton, 2004 [119]	no	yes	no/no	Database	3d DR, Counselling	Key fat sources, breakfast cereals, bread	11.4	1.0	0.0	1.0	1.0	1.0	1.0	0.0	1.0	1.0	0.0	1.0
Noakes, 2005 [78]	no	yes	no/no	Database	3d DR, Urine N, minerals, vitamins	60	15.8	1.0	0.0	1.0	1.0	1.0	1.0	0.0	1.0	1.0	0.0	1.0
Luscombe-Marsh, 2005 [77]	no	yes	no/no	Database	Urine N, Counselling	60	20.6	1.0	0.0	1.0	1.0	1.0	1.0	0.5	1.0	1.0	0.0	1.0
Keogh, 2005 [120]	no	no	no/no	Database	3d DR	85.5	20.0	1.0	0.0	1.0	1.0	1.0	1.0	0.0	1.0	1.0	0.0	1.0
Schutte, 2006 [121]	no	no	no/no	Meal	Leftover, FD	100	8.8	1.0	0.0	0.5	1.0	1.0	1.0	1.0	1.0	1.0	0.0	1.0
Noakes, 2006 [34]	no	yes	no/no	Database	3d DR	36	19.3	1.0	0.5	1.0	0.5	1.0	0.5	0.5	1.0	1.0	0.5	1.0
Vega-Lopez, 2006 [107]	no	no	yes/yes	Meal	NR	100	N/A	1.0	1.0	0.0	1.0	1.0	1.0	0.0	1.0	1.0	0.0	1.0
Tholstrup, 2006 [97]	no	no	no/no	Fat	4d DR, TG FA, CE FA, PL FA	60	N/A	1.0	1.0	0.0	1.0	1.0	0.5	0.5	1.0	1.0	0.0	1.0
Johnston, 2006 [120]	no	yes	no/no	Database	PL FA	100	5.0	1.0	0.5	1.0	1.0	1.0	0.0	0.5	1.0	1.0	0.5	1.0
Tricon, 2006 [37]	no	no	yes/yes	Database	3d DR, LDL FA	12.1	3.1	1.0	1.0	1.0	1.0	1.0	1.0	0.0	1.0	1.0	0.0	1.0
Berglund, 2007 [105]	no	no	no/no	Meal	Counselling	85.7	22.7	1.0	1.0	0.5	1.0	1.0	1.0	0.0	1.0	1.0	0.0	1.0
St-Onge, 2007 [38]	no	no	no/no	Database	Counselling	100	26.7	1.0	0.0	1.0	1.0	1.0	1.0	0.0	1.0	1.0	0.0	1.0
Sundram, 2007 [33]	no	no	no/no	Fat, protein	TG FA	100	6.3	1.0	1.0	1.0	1.0	1.0	1.0	0.0	1.0	1.0	0.0	1.0
Paniagua, 2007 [108]	no	no	no/no	Database	3d DR, Plasma FA	100	3.0	1.0	0.0	1.0	1.0	1.0	1.0	0.5	1.0	1.0	0.0	1.0
Egert, 2008 [106]	no	no	no/no	Fat	FD, LDL FA	100	21.3	1.0	0.5	0.5	1.0	1.0	1.0	0.0	0.5	1.0	0.5	1.0
Mensink, 2008 [85]	no	no	no/yes	Fat	PL FA	15	0	1.0	1.0	1.0	1.0	1.0	1.0	1.0	1.0	1.0	0.0	1.0
Tay, 2008 [122]	no	yes	no/no	Database	3d DR	30	19.3	1.0	0.0	1.0	1.0	1.0	1.0	0.0	1.0	1.0	0.0	1.0
Vega-Lopez, 2009 [113]	no	no	no/no	Meal	N/A	100	18.9	0.5	1.0	0.0	1.0	1.0	1.0	0.0	1.0	1.0	0.0	1.0
Bradley, 2009 [62]	no	yes	no/no	Database	Counselling, Urine N	100	11.1	1.0	0.5	0.0	1.0	0.5	1.0	1.0	1.0	1.0	0.0	1.0
Tardy, 2009 [30]	no	no	yes/yes	Fat	5d DR, PL FA	Key foods	7.9	1.0	1.0	1.0	1.0	1.0	1.0	0.5	1.0	1.0	0.0	1.0
Forsythe, 2010 [114]	no	no	no/no	Database	Leftover, Counselling, TG FA, PL FA, CE	100	N/A	1.0	0.0	0.0	1.0	1.0	1.0	0.0	1.0	0.5	0.0	1.0

S2 Table. Characteristics and scores of reporting quality of 102 trials eligible for the meta-analysis of randomized controlled feeding trials of macronutrient intakes and glycaemic outcomes.

								Assessment of quality ‖										
Author of trial, year	Ward	Weight loss (caloric restriction)	Blinding intervention /outcome measures*	Sources of food composition †	Compliance assessment ‡	Food provided, % of energy or text §	Drop out %	Randomisation	Blinding	Drop out	Study objectives	Outcome definition	Eligibility criteria	Sample size	Exposure definition	Control arm(s)	Adverse effects	Statistical methods
					FA													
Jebb, 2010 [27]	no	no	no/no	Meal, fat, database	FD, PL FA	Key sources of fat and carbohydrates	27.1	1.0	0.0	1.0	1.0	1.0	1.0	0.0	1.0	1.0	0.0	1.0
Bos, 2010 [123]	no	no	no/no	Meal	FD, adipocyte FA	90	5.1	1.0	0.0	1.0	1.0	1.0	1.0	1.0	1.0	1.0	0.5	1.0
Yeung, 2010 [47]	no	no	no/no	Database	Counselling	100	15.7	1.0	0.0	0.5	1.0	0.5	1.0	0.5	1.0	1.0	0.5	1.0
Johnstone, 2011 [92]	no	yes	no/no	Database	FD	100	11.1	1.0	0.0	0.5	1.0	1.0	1.0	0.0	1.0	1.0	0.0	0.5
Iggman, 2011 [40]	no	no	no/no	Fat	CE FA, PL FA	100	NR	1.0	0.5	0.5	1.0	1.0	1.0	0.0	1.0	1.0	0.0	1.0
Hjerpsted, 2011 [98]	no	no	no/no	Database	3d DR	13	7.5	1.0	0.0	0.0	1.0	1.0	1.0	0.0	0.5	1.0	0.0	0.5
Tholstrup, 2011 [124]	no	no	no/no	Fat	3d DR	17	4.4	1.0	1.0	0.0	1.0	1.0	1.0	0.0	0.5	1.0	0.0	0.5
Goree, 2011 [46]	no	no	no/no	Database	N/A	100	9.2	0.0	0.5	0.5	1.0	1.0	1.0	0.0	1.0	1.0	0.0	1.0
Tierney, 2011[125]	no	no	no/yes	Database	3d FD, Plasma FA	72.2	14.2	1.0	0.0	0.5	1.0	1.0	1.0	1.0	1.0	1.0	0.0	1.0
Bendsen, 2011 [91]	no	no	yes/yes	Database	3d DR, PL FA, RBC FA	31.2	5.7	1.0	1.0	0.0	1.0	1.0	1.0	1.0	1.0	1.0	0.0	1.0
Haghighatdoost, 2012 [49]	no	yes	no/yes	Database	1d DR	High-fat dairy, butter, red meat	34.6	1.0	0.5	1.0	1.0	1.0	1.0	0.5	1.0	1.0	0.5	1.0
Guay, 2012 [109]	no	no	yes/yes	Database	N/A	100	N/A	1.0	0.5	0.0	1.0	1.0	1.0	0.0	0.5	1.0	0.0	1.0
Bjermo, 2012 [48]	no	no	no/yes	Database	CE FA	50	4.7	1.0	0.0	1.0	1.0	1.0	1.0	1.0	1.0	1.0	1.0	1.0
Wycherley, 2012 [126]	no	yes	no/no	Database	3d DR	60	8.4	1.0	0	1.0	1.0	1.0	1.0	1.0	1.0	1.0	0.0	1.0
Kien, 2013 [24]	no	no	yes/yes	Database	Muscle FA, Blood FA, Monitoring	100	25.0	0.5	0.0	0.5	1.0	1.0	1.0	0.5	1.0	1.0	0.0	0.0
Young, 2013 [94]	no	no	no/no	Database	PL FA	100	29.2	1.0	0.0	1.0	1.0	1.0	1.0	0.0	1.0	1.0	0.5	1.0
Rietman, 2014	no	no	no/no	Meal	Urine N, FD, Leftover	100	6.9	1.0	0.5	0.5	1.0	1.0	0.0	0.5	1.0	1.0	0.0	1.0
Iggman, 2014 [51]	no	no	yes/yes	Fat	CE FA, 4d DR	7.65	0	1.0	1.0	1.0	1.0	1.0	1.0	1.0	1.0	1.0	0.5	1.0
Jordy, 2014 [127]	no	no	no/no	N/A	N/A	50	N/A	1.0	0.0	0.0	1.0	1.0	0.0	0.0	0.0	1.0	0.0	0.0
Marina, 2014 [128]	no	no	no/no	Database	Checklist	100	46.2	1.0	0.0	0.0	1.0	1.0	1.0	0.0	1.0	1.0	0.5	1.0
Sacks, 2014 [26]	no	no	no/yes	Database	Urine N, Leftover, Monitoring	100	5.9	1.0	0.0	1.0	1.0	1.0	1.0	1.0	1.0	1.0	1.0	1.0
Filippou, 2014 [100]	no	no	yes/yes	Fat	Lipids	71.4	29.3	1.0	0.5	1.0	1.0	1.0	1.0	1.0	1.0	0.5	0.5	1.0
Chiu, 2014 [50]	no	no	no/no	N/A	Counselling	66.0	9.2	1.0	0.0	1.0	1.0	1.0	1.0	0.5	0.5	1.0	0.5	1.0
Baril-Gravel, 2015 [52]	no	no	no/yes	Fat	Serum lipids	100	19.2	1.0	1.0	1.0	1.0	1.0	1.0	1.0	1.0	1.0	0.0	1.0
Vafeiadou, 2015 [96]	no	no	no/no	Meal	PL FA, 4d FR	26.6	6.9	1.0	0.0	1.0	1.0	1.0	1.0	1.0	1.0	1.0	0.0	1.0

S2 Table. Characteristics and scores of reporting quality of 102 trials eligible for the meta-analysis of randomized controlled feeding trials of macronutrient intakes and glycaemic outcomes.

								Assessment of quality ‖										
Author of trial, year	Ward	Weight loss (caloric restriction)	Blinding intervention /outcome measures*	Sources of food composition †	Compliance assessment ‡	Food provided, % of energy or text §	Drop out %	Randomisation	Blinding	Drop out	Study objectives	Outcome definition	Eligibility criteria	Sample size	Exposure definition	Control arm(s)	Adverse effects	Statistical methods
Rozati, 2015 [101]	no	no	no/yes	Fat	Counselling, Plasma FA	17.1	6.8	1.0	0.5	1.0	1.0	1.0	1.0	0.0	1.0	1.0	0.0	1.0
**Trials recruiting adults with diabetes**																		
Garg, 1988 [130]	yes	no	no/no	Fat	TG FA, CE FA	100	0	1.0	0.0	1.0	1.0	1.0	0.5	0.0	1.0	1.0	0.0	1.0
Coulston, 1989 [55]	no	no	no/no	Database	N/A	100	0	1.0	0.0	0.5	1.0	1.0	0.0	0.0	0.5	1.0	0.0	1.0
Fuh, 1990 [57]	no	no	no/no	Database	N/A	100	N/A	1.0	0.0	0.0	1.0	1.0	0.0	0.0	1.0	1.0	0.0	1.0
Vessby, 1992 [84]	yes	no	no/no	Fat	PL FA	100	6.7	0.0	0.0	1.0	1.0	1.0	0.5	0.0	0.5	1.0	0.0	0.5
Parillo, 1992 [89]	yes	no	no/no	Database	N/A	100	0	1.0	0.5	0.5	1.0	1.0	0.5	0.0	1.0	1.0	0.0	1.0
Garg, 1992 [61]	yes	no	no/no	Database	N/A	100	N/A	1.0	0.0	0.0	1.0	1.0	0.5	0.0	1.0	1.0	0.0	1.0
Garg, 1992 [131]	yes	no	no/no	Database	N/A	100	0	1.0	0.0	1.0	1.0	1.0	1.0	0.0	1.0	1.0	0.5	1.0
Rasmussen, 1993 [67]	no	no	no/no	Database	3d DR	Key caloric foods	0	1.0	0.0	0.5	1.0	1.0	1.0	0.0	1.0	1.0	0.0	1.0
Garg, 1994 [88]	no	no	no/no	Database	N/A	100	0	1.0	0.0	0.5	1.0	1.0	0.0	0.0	1.0	1.0	0.0	1.0
Lerman-Garber, 1994 [102]	no	no	no/no	Database	24hR	100	25.0	1.0	0.0	1.0	1.0	0.5	0.0	0.0	0.5	1.0	0.0	0.0
Walker, 1995 [82]	no	no	no/no	Database	Counselling	Olive oil	N/A	1.0	0.0	0.0	1.0	0.5	0.0	0.0	1.0	1.0	0.0	1.0
Thomsen, 1995 [63]	no	no	no/no	Database	3d DR	Oils, rolls, meat dishes	N/A	1.0	0.0	0.5	1.0	1.0	0.5	0.0	1.0	1.0	0.0	1.0
Lerman-Garber, 1995 [83]	no	no	no/no	Database	7d DR, Counselling	100	35.0	1.0	0.0	1.0	1.0	1.0	1.0	0.0	1.0	1.0	0.0	0.0
Katsilambros, 1996 [104]	no	no	no/no	Database	Counselling	oil only	N/A	1.0	0.0	0.0	0.5	1.0	0.5	0.5	1.0	1.0	0.0	1.0
Storm, 1997 [132]	no	no	no/no	Fat	7d DR, TG FA	62	0	1.0	0.0	0.5	1.0	1.0	0.5	0.0	1.0	1.0	0.0	1.0
Christiansen, 1997 [115]	no	no	no/no	Database	Counselling	key foods	N/A	1.0	0.0	0.0	1.0	1.0	1.0	0.0	1.0	1.0	0.0	0.5
Georgopoulos, 1998 [35]	no	no	no/no	Database	N/A	100	N/A	1.0	0.0	0.0	0.5	1.0	0.0	0.0	1.0	1.0	0.5	0.5
Brynes, 2000 [60]	no	no	yes/yes	Database	TG FA	Carrot cake	N/A	0.5	1.0	0.5	1.0	1.0	0.0	0.5	1.0	1.0	0.0	1.0
Shige, 2000 [65]	no	yes	no/no	Database	3d DR	Key caloric foods, wheat bran, breakfast cereal, and low-fat frozen meals	16.7	1.0	0.0	1.0	1.0	1.0	0.5	0.0	1.0	1.0	0.0	1.0
Higashi, 2001 [103]	yes	no	no/no	Database	N/A	100	0	1.0	0.0	0.0	1.0	1.0	0.0	0.0	1.0	1.0	0.0	1.0
Lovejoy, 2002 [25]	no	no	no/no	Meal	FD	100	11.8	1.0	0.5	1.0	1.0	1.0	1.0	0.0	1.0	1.0	0.0	1.0
Rodriguez-Villar, 2003	no	no	no/no	Database	3d DR	25	15.4	1.0	0.0	1.0	1.0	1.0	1.0	1.0	0.0	1.0	0.0	1.0

S2 Table. Characteristics and scores of reporting quality of 102 trials eligible for the meta-analysis of randomized controlled feeding trials of macronutrient intakes and glycaemic outcomes.

Author of trial, year	Ward	Weight loss (caloric restriction)	Blinding intervention /outcome measures*	Sources of food composition †	Compliance assessment ‡	Food provided, % of energy or text §	Drop out %	Assessment of quality || Randomisation	Blinding	Drop out	Study objectives	Outcome definition	Eligibility criteria	Sample size	Exposure definition	Control arm(s)	Adverse effects	Statistical methods
[90]																		
Gannon, 2004 [41]	no	no	no/no	Database	Urine N, Counselling	100	27.3	1.0	0.0	1.0	1.0	1.0	0.5	0.0	1.0	1.0	0.0	1.0
Miyashita, 2004 [133]	yes	yes	no/no	Database	N/A	100	N/A	0.0	0.0	0.0	1.0	1.0	0.0	0.0	1.0	1.0	0.0	0.0
Papakonstantinou, 2010 [134]	no	yes	no/no	Database	DR	26	N/A	1.0	1.0	0.5	1.0	1.0	1.0	1.0	1.0	1.0	1.0	1.0
Taylor, 2010 [135]	no	no	no/no	Database	3d DR, 24hR, PL FA	Bakery products	N/A	1.0	0.0	0.0	1.0	1.0	1.0	1.0	1.0	1.0	0.0	1.0
Li, 2011 [93]	no	no	no/no	Database	FD	100	9.1	1.0	0.0	1.0	1.0	1.0	1.0	0.0	1.0	1.0	0.0	1.0
Jenkins, 2011 [136]	no	no	no/no	Database	7d DR	Nuts, muffins	12.1	1.0	0.0	1.0	1.0	1.0	1.0	1.0	1.0	1.0	0.5	1.0
Gannon, 2011 [45]	no	no	no/no	Database	FD	100	N/A	1.0	0.0	0.0	1.0	1.0	0.5	0.0	1.0	1.0	0.0	1.0
Helge, 2012 [99]	no	no	no/no	Database	N/A	100	N/A	1.0	0.5	0.0	1.0	1.0	0.0	0.0	1.0	1.0	0.0	1.0
Tay, 2015 [95]	no	yes	no/yes	Database	7d DR, OH-B	Key foods	33.1	1.0	0.0	1.0	1.0	1.0	1.0	1.0	1.0	1.0	1.0	1.0

The reference list is in S3 Text. Abbreviations: N/A, not available in the publication in which there was no information on those who did not complete follow-up; DR, dietary records; FA, fatty acid; FD, food diary; 24hR, 24-hour dietary recall; CE, cholesteryl ester; PL, phospholipid; RBC, red blood cell; TG, triglyceride; Urine N, urinary nitrogen.

* Trials that reported interventions to achieve blinding, for example, testing two fat spreads made of different types of vegetable oils (e.g. palm oil and rapeseed oil), in which study participants were unlikely to be aware of difference in experimental diets. Of outcome assessments, “no” was assigned for trials that did not report any information on blinding of outcome measurements.

† Database indicates amount of nutrients consumed were calculated by using nutrient database. The others indicate that authors determined macronutrient compositions (meals) or fat composition (fat), or protein contents (protein) by conducting a laboratory assay of test meals.

‡ Assessment of compliance to each dietary intervention. Blank indicates no information was available. Counselling includes assessment of compliance by interview and by use of dietary questionnaires. Each of protein and FA indicates that trials performed urine assay to relate urinary nitrogen to protein intake and FA assays to relate circulating FA levels to FA intake. "Leftover" indicates that the investigators collected leftovers or packages of meals provided and confirmed compliance to the meal intervention.

§ Values represent % of energy provided by the study group to each participant (>0 to 100 because we included only feeding trials). If not available, text description of trial intervention is provided.

|| Each of the eleven components was scored as 0 or 1 by two independent reviewers and averaged to be 0, 0.5 or 1.0. The sum of the eleven scores, specified by Jadad et al, was considered as a measure of study quality.[138] The inter-rater correlation was 0.61, consistent with the observation by Jadad et al. The presentation follows the recommendation by Cochrane Collaboration.[139]
