# Supplementary material for: Effects of Saturated Fat, Polyunsaturated Fat, Monounsaturated Fat, and Carbohydrate on Glucose-Insulin Homeostasis: A Systematic Review and Meta-analysis of Randomised Controlled Feeding Trials
Source: PLoS Med. 2016 Jul 19;13(7):e1002087. doi: 10.1371/journal.pmed.1002087 (PMC4951141; doi:10.1371/journal.pmed.1002087)
Supplement: S3 Table — (PDF) [file pmed.1002087.s005.pdf]

S3 Table. Effects of isocalorically exchanging 5% of dietary energy between carbohydrates and major types of fatty acids on glucose-insulin metrics, with stratification by country, age, sex, diabetes status, provision of meals, and blinding in randomised controlled feeding trials.*

Outcome	Strata of trials*	N trials	Effects (95% confidence interval) of isocaloric replacement of 5% dietary energy					
			CHO →SFA	CHO →MUFA	CHO →PUFA	SFA →MUFA	SFA →PUFA	MUFA →PUFA
Glucose, mnol/L								
All		99	0.02 (-0.01, 0.04)	0.00 (-0.02, 0.02)	-0.02 (-0.05, 0.01)	-0.02 (-0.04, 0.00)	-0.04 (-0.07, -0.01)	-0.02 (-0.05, 0.01)
Region	US/Canada	33	0.03 (-0.01, 0.08)	0.02 (-0.03, 0.06)	-0.03 (-0.06, 0.01)	-0.02 (-0.05, 0.02)	-0.06 (-0.11, -0.01)	-0.04 (-0.10, 0.02)
	Europe	56	0.00 (-0.04, 0.04)	0.00 (-0.03, 0.02)	-0.03 (-0.08, 0.01)	-0.01 (-0.05, 0.04)	-0.04 (-0.08, 0.00)	-0.03 (-0.09, 0.03)
	Asia	7	-0.13 (-0.32, 0.05)	-0.17 (-0.31, -0.02)	0.14 (-0.14, 0.41)	-0.03 (-0.11, 0.05)	0.27 (-0.11, 0.66)	0.30 (-0.02, 0.62)
Age	≥52 years	58	0.01 (-0.03, 0.05)	-0.04 (-0.08, -0.01)	-0.08 (-0.12, -0.04)	-0.05 (-0.10, 0.00)	-0.09 (-0.13, -0.04)	-0.04 (-0.09, 0.02)
	<52 years	41	0.04 (0.01, 0.07)	0.02 (0.00, 0.05)†	0.02 (-0.03, 0.06)†	-0.02 (-0.04, 0.01)	-0.02 (-0.07, 0.03)	-0.01 (-0.06, 0.04)
Sex	Men	49	-0.01 (-0.07, 0.04)	-0.04 (-0.06, -0.01)	-0.08 (-0.13, -0.03)	-0.02 (-0.07, 0.03)	-0.06 (-0.11, -0.01)	-0.04 (-0.09, 0.01)
	Women	50	0.03 (0.01, 0.05)	0.01 (-0.01, 0.04)†	0.00 (-0.04, 0.03)	-0.02 (-0.04, 0.01)	-0.03 (-0.07, 0.01)	-0.02 (-0.06, 0.02)
Body mass index	≥28 kg/m^2	47	0.04 (0.00, 0.07)	-0.03 (-0.06, -0.01)	-0.03 (-0.08, 0.02)	-0.07 (-0.12, -0.02)	-0.07 (-0.12, -0.02)	0.00 (-0.06, 0.06)
	<28 kg/m^2	52	0.04 (0.00, 0.07)	0.03 (0.00, 0.06)†	0.01 (-0.04, 0.05)	-0.01 (-0.04, 0.01)	-0.03 (-0.07, 0.01)	-0.02 (-0.07, 0.02)
Diabetes	Yes	31	-0.18 (-0.43, 0.08)	-0.14 (-0.22, -0.06)	-0.32 (-0.50, -0.14)	0.03 (-0.26, 0.33)	-0.15 (-0.48, 0.19)	-0.18 (-0.39, 0.03)
	No	68	0.03 (0.00, 0.05)	0.01 (-0.01, 0.03)†	-0.01 (-0.04, 0.02)†	-0.02 (-0.04, 0.00)	-0.04 (-0.07, 0.00)	-0.02 (-0.05, 0.02)
Providing meals	All	53	0.04 (0.00, 0.09)	-0.02 (-0.05, 0.01)	0.01 (-0.02, 0.05)	-0.07 (-0.10, -0.03)	-0.03 (-0.09, 0.02)	0.04 (-0.01, 0.08)
	Partial	46	0.00 (-0.02, 0.03)	0.01 (-0.01, 0.03)	-0.05 (-0.08, -0.02)	0.01 (-0.01, 0.03)	-0.05 (-0.08, -0.02)	-0.06 (-0.10, -0.03)
Blinding	Yes	60	0.01 (-0.04, 0.05)	-0.05 (-0.07, -0.02)	-0.06 (-0.13, 0.01)	-0.05 (-0.10, -0.01)	-0.07 (-0.14, 0.01)	-0.01 (-0.08, 0.05)
	No	39	0.07 (0.03, 0.10)	0.06 (0.03, 0.10)†	0.02 (-0.02, 0.06)	0.00 (-0.03, 0.02)	-0.05 (-0.07, -0.02)	-0.04 (-0.08, 0.00)
HbA1c, %								
All		23	0.03 (-0.02, 0.09)	-0.09 (-0.12, -0.05)	-0.11 (-0.17, -0.05)	-0.12 (-0.19, -0.05)	-0.15 (-0.23, -0.06)	-0.03 (-0.09, 0.03)
Region	US/Canada	10	0.59 (0.21, 0.97)	-0.10 (-0.20, -0.01)	-0.50 (-0.73, -0.28)	-0.69 (-1.08, -0.31)	-1.09 (-1.46, -0.72)	-0.40 (-0.66, -0.13)
	Europe	12	0.04 (-0.04, 0.11)	-0.05 (-0.10, 0.00)	-0.07 (-0.22, 0.08)	-0.09 (-0.15, -0.02)	-0.11 (-0.24, 0.02)†	-0.02 (-0.14, 0.10)
	Asia	0	Not estimated‡					
Age	≥52 years	18	0.01 (-0.05, 0.08)	-0.10 (-0.16, -0.04)	-0.14 (-0.29, 0.01)	-0.11 (-0.18, -0.04)	-0.15 (-0.32, 0.01)	-0.04 (-0.21, 0.12)
	<52 years	5	0.49 (-0.61, 1.59)	0.01 (-0.95, 0.98)	0.23 (-2.11, 2.58)	-0.47 (-1.26, 0.31)	-0.25 (-1.85, 1.34)	0.22 (-1.17, 1.61)
Sex	Men	12	0.29 (-0.30, 0.89)	-0.05 (-0.19, 0.10)	-0.10 (-0.25, 0.05)	-0.34 (-0.95, 0.27)	-0.39 (-0.98, 0.20)	-0.05 (-0.15, 0.05)
	Women	11	-0.01 (-0.07, 0.04)	-0.08 (-0.11, -0.04)	-0.07 (-0.14, -0.01)	-0.07 (-0.13, 0.00)	-0.06 (-0.15, 0.03)	0.01 (-0.06, 0.07)
Body mass index	≥28 kg/m^2	12	0.01 (-0.04, 0.07)	-0.10 (-0.14, -0.06)	-0.14 (-0.27, -0.02)	-0.12 (-0.17, -0.06)	-0.16 (-0.32, 0.00)	-0.04 (-0.19, 0.11)
	<28 kg/m^2	11	-0.15 (-0.53, 0.23)	-0.08 (-0.29, 0.13)	-0.11 (-0.36, 0.14)	0.08 (-0.21, 0.36)	0.04 (-0.21, 0.30)	-0.03 (-0.10, 0.04)
Diabetes	Yes	18	-0.11 (-0.29, 0.08)	-0.16 (-0.25, -0.06)	-0.18 (-0.33, -0.03)	-0.05 (-0.27, 0.17)	-0.08 (-0.26, 0.11)	-0.02 (-0.20, 0.15)
	No	5	-0.02 (-0.20, 0.17)	-0.09 (-0.19, 0.01)	-0.11 (-0.20, -0.01)	-0.07 (-0.18, 0.03)	-0.09 (-0.23, 0.05)	-0.02 (-0.08, 0.04)
Providing meals	All	16	0.12 (0.03, 0.20)	-0.02 (-0.08, 0.03)	-0.21 (-0.41, -0.02)	-0.14 (-0.20, -0.07)	-0.33 (-0.58, -0.07)	-0.19 (-0.42, 0.04)
	Partial	7	-0.06 (-0.29, 0.18)	-0.15 (-0.34, 0.05)	-0.19 (-0.42, 0.04)	-0.09 (-0.35, 0.17)	-0.13 (-0.38, 0.11)	-0.04 (-0.15, 0.06)
Blinding	Yes	16	0.00 (-0.06, 0.06)	-0.10 (-0.15, -0.05)	-0.15 (-0.28, -0.01)	-0.11 (-0.17, -0.04)	-0.15 (-0.32, 0.02)	-0.04 (-0.21, 0.12)
	No	7	0.38 (-0.14, 0.90)	-0.08 (-0.27, 0.10)	-0.07 (-0.26, 0.12)	-0.46 (-1.09, 0.16)	-0.46 (-1.06, 0.15)	0.01 (-0.06, 0.08)

S3 Table. Effects of isocalorically exchanging 5% of dietary energy between carbohydrates and major types of fatty acids on glucose-insulin metrics, with stratification by country, age, sex, diabetes status, provision of meals, and blinding in randomised controlled feeding trials.*

Outcome	Strata of trials*	N trials	Effects (95% confidence interval) of isocaloric replacement of 5% dietary energy					
			CHO →SFA	CHO →MUFA	CHO →PUFA	SFA →MUFA	SFA →PUFA	MUFA →PUFA
Insulin, pmol/L								
All		90	-1.12 (-1.72, -0.53)	0.05 (-0.26, 0.36)	-1.60 (-2.77, -0.42)	1.17 (0.57, 1.78)	-0.47 (-2.01, 1.07)	-1.65 (-2.84, -0.45)
Region	US/Canada	28	-1.04 (-2.19, 0.10)	0.90 (-0.18, 1.98)	-3.47 (-5.05, -1.90)	1.94 (1.11, 2.78)	-2.43 (-4.28, -0.58)	-4.37 (-6.40, -2.35)
	Europe	55	-0.73 (-3.07, 1.62)	0.21 (-0.29, 0.71)	-0.69 (-2.03, 0.64)	0.93 (-1.54, 3.41)	0.03 (-3.27, 3.34)	-0.90 (-2.13, 0.33)
	Asia	7	-2.94 (-6.38, 0.50)	-2.59 (-5.08, -0.11)	-6.70 (-12.1, -1.31)	0.35 (-1.52, 2.22)	-3.76 (-11.09, 3.57)	-4.11 (-10.42, 2.19)
Age	≥52 years	53	0.00 (-1.19, 1.19)	-0.50 (-1.18, 0.18)	-1.89 (-2.74, -1.04)	-0.50 (-1.95, 0.95)	-1.89 (-3.31, -0.46)	-1.39 (-2.35, -0.43)
	<52 years	37	-1.01 (-1.73, -0.30)	0.18 (-0.42, 0.78)	-0.10 (-4.24, 4.04)	1.20 (0.65, 1.74)	0.91 (-3.06, 4.89)	-0.28 (-4.18, 3.62)
Sex	Men	46	-1.57 (-2.34, -0.80)	0.02 (-0.54, 0.57)	-1.58 (-2.43, -0.73)	1.59 (0.73, 2.44)	-0.01 (-1.09, 1.07)	-1.60 (-2.53, -0.66)
	Women	44	-0.93 (-1.79, -0.07)	-0.06 (-0.61, 0.48)	-1.65 (-3.68, 0.38)	0.87 (-0.01, 1.75)	-0.72 (-3.29, 1.85)	-1.59 (-3.66, 0.48)
Body mass index	≥28 kg/m^2	41	0.19 (-1.05, 1.42)	-0.22 (-1.00, 0.57)	-1.72 (-2.64, -0.79)	-0.40 (-2.07, 1.26)	-1.90 (-3.47, -0.34)	-1.50 (-2.55, -0.45)
	<28 kg/m^2	49	-1.47 (-2.23, -0.72)	-0.05 (-0.59, 0.49)	-1.66 (-3.85, 0.52)	1.42 (0.82, 2.03)	-0.19 (-2.71, 2.33)	-1.61 (-3.89, 0.67)
Diabetes	Yes	25	-2.51 (-5.39, 0.37)	-1.96 (-3.67, -0.26)	-3.33 (-5.37, -1.29)	0.55 (-2.97, 4.08)	-0.82 (-4.47, 2.84)	-1.37 (-2.49, -0.24)
	No	65	-0.96 (-1.56, -0.36)	0.22 (-0.15, 0.59)	-1.23 (-3.03, 0.57)	1.18 (0.63, 1.73)	-0.27 (-2.39, 1.85)	-1.45 (-3.33, 0.44)
Providing meals	All	52	-1.36 (-2.25, -0.46)	0.53 (-0.21, 1.27)	-2.51 (-3.88, -1.14)	1.89 (1.07, 2.71)	-1.15 (-2.70, 0.40)	-3.04 (-4.72, -1.36)
	Partial	38	-0.91 (-1.78, -0.05)	-0.05 (-0.54, 0.43)	-1.39 (-2.93, 0.14)	0.86 (-0.09, 1.82)	-0.48 (-2.51, 1.55)	-1.34 (-2.74, 0.06)
Blinding	Yes	52	-0.34 (-1.66, 0.98)	-0.14 (-0.58, 0.30)	-1.52 (-2.80, -0.24)	0.21 (-1.12, 1.53)	-1.18 (-3.05, 0.69)	-1.39 (-2.65, -0.12)
	No	38	-1.50 (-2.36, -0.63)	-0.14 (-0.92, 0.65)	-1.96 (-3.49, -0.44)	1.36 (0.68, 2.04)	-0.47 (-2.32, 1.39)	-1.83 (-3.38, -0.28)
HOMA, %change								
All		30	0.7 (-1.6, 3.1)	-2.4 (-4.6, -0.3)	-3.4 (-5.9, -0.8)	-3.1 (-5.8, -0.4)	-4.1 (-6.4, -1.6)	-1.0 (-4.4, 2.6)
Region	US/Canada	8	0.0 (-3.6, 3.7)	1.0 (-5.6, 8.2)	-4.9 (-8.6, -1.0)	1.1 (-7.8, 10.7)	-4.8 (-7.5, -2.2)	-5.8 (-14.7, 3.9)
	Europe	18	3.1 (-3.0, 9.5)	-3.4 (-8.3, 1.8)	1.7 (-6.1, 10.0)	-6.3 (-10.5, -1.8)	-1.4 (-9.6, 7.7)	5.2 (-4.5, 15.9)
	Asia	4	Not estimated‡					
Age	≥52 years	17	1.4 (-1.2, 4.2)	-3.0 (-5.7, -0.2)	-4.1 (-6.9, -1.1)	-4.4 (-8.2, -0.4)	-5.4 (-8.1, -2.7)	-1.1 (-5.7, 3.7)
	<52 years	13	-1.5 (-11.2, 9.3)	-4.3 (-12.7, 4.8)	1.6 (-8.7, 13.1)	-2.9 (-7.8, 2.2)	3.2 (-2.7, 9.4)	6.2 (-1.8, 14.9)
Sex	Men	13	-4.0 (-9.2, 1.5)	-4.9 (-11.4, 2.1)	-6.8 (-12.2, -1.1)	-0.9 (-10.9, 10.2)	-2.9 (-6.3, 0.6)	-2.0 (-12.5, 9.8)
	Women	17	2.3 (-0.5, 5.3)	-1.9 (-4.5, 0.8)	-2.8 (-6.2, 0.7)	-4.1 (-7.3, -0.9)	-5.0 (-8.5, -1.5)	-0.9 (-5.3, 3.6)
Body mass index	≥28 kg/m^2	15	1.4 (-1.3, 4.2)	-2.7 (-5.7, 0.4)	-3.2 (-6.4, 0.0)	-4.1 (-8.3, 0.3)	-4.6 (-7.6, -1.5)	-0.5 (-5.7, 4.9)
	<28 kg/m^2	15	-3.4 (-10.7, 4.6)	-5.2 (-10.9, 0.8)	-6.3 (-13.0, 1.0)	-1.9 (-6.9, 3.3)	-3.0 (-6.9, 1.1)	-1.1 (-6.8, 4.9)
Diabetes	Yes	6	Not estimated‡					
	No	24	2.0 (-0.7, 4.8)	-1.3 (-3.9, 1.3)	-1.9 (-4.7, 1.0)	-3.3 (-6.5, 0.0)	-3.9 (-6.3, -1.4)	-0.6 (-4.6, 3.5)
Providing meals	All	15	2.6 (-0.2, 5.4)	-3.9 (-6.8, -0.8)	-1.4 (-4.5, 1.7)	-6.3 (-10.3, -2.1)	-3.9 (-6.4, -1.3)	2.5 (-2.5, 7.8)
	Partial	15	-1.8 (-8.4, 5.3)	-3.4 (-9.6, 3.1)	-6.2 (-12.9, 1.0)	-1.7 (-6.4, 3.3)	-4.5 (-13.8, 5.8)	-2.9 (-13.1, 8.5)
Blinding	Yes	17	-1.0 (-4.8, 3.0)	-5.6 (-8.7, -2.5)	-4.8 (-10.4, 1.1)	-4.7 (-8.7, -0.5)	-3.9 (-9.1, 1.7)	0.9 (-5.8, 8.1)
	No	13	1.0 (-2.3, 4.4)	0.3 (-5.2, 6.2)	-3.5 (-6.7, -0.2)	-0.7 (-7.5, 6.6)	-4.5 (-7.3, -1.7)	-3.8 (-10.7, 3.6)

S3 Table. Effects of isocalorically exchanging 5% of dietary energy between carbohydrates and major types of fatty acids on glucose-insulin metrics, with stratification by country, age, sex, diabetes status, provision of meals, and blinding in randomised controlled feeding trials.*

Outcome	Strata of trials*	N trials	Effects (95% confidence interval) of isocaloric replacement of 5% dietary energy					
			CHO →SFA	CHO →MUFA	CHO →PUFA	SFA →MUFA	SFA →PUFA	MUFA →PUFA
2h glucose, mmol/L								
All		11	-0.04 (-0.39, 0.31)	-0.15 (-0.76, 0.47)	0.21 (-0.35, 0.78)	-0.10 (-0.91, 0.70)	0.26 (-0.34, 0.85)	0.36 (-0.48, 1.20)
Region	US/Canada	3	Not estimated‡					
	Europe	7	0.08 (-0.55, 0.72)	-0.42 (-0.79, -0.04)	0.21 (-0.57, 0.99)	-0.50 (-1.22, 0.22)	0.13 (-0.73, 0.98)	0.63 (-0.07, 1.33)
	Asia	1	Not estimated‡					
Age	≥52 years	7	0.12 (-0.43, 0.67)	0.13 (-0.33, 0.59)	0.26 (-0.55, 1.07)	0.01 (-0.76, 0.79)	0.14 (-0.74, 1.03)	0.13 (-0.70, 0.95)
	<52 years	4	Not estimated‡					
Sex	Men	6	0.02 (-1.05, 1.08)	0.16 (-0.36, 0.68)	0.17 (-0.84, 1.18)	0.14 (-1.24, 1.53)	0.15 (-1.31, 1.60)	0.00 (-1.05, 1.06)
	Women	5	0.08 (-0.29, 0.45)	-0.15 (-1.46, 1.15)	0.55 (-0.97, 2.08)	-0.24 (-1.66, 1.19)	0.47 (-1.14, 2.08)	0.71 (-1.34, 2.75)
Body mass index	≥28 kg/m^2	6	-0.14 (-0.71, 0.43)	0.11 (-0.29, 0.51)	-0.38 (-0.98, 0.22)	0.25 (-0.59, 1.09)	-0.24 (-0.93, 0.46)	-0.49 (-1.35, 0.37)
	<28 kg/m^2	5	Not estimated‡					
Diabetes	Yes	6	0.23 (-0.43, 0.88)	-0.16 (-1.41, 1.10)	0.63 (-0.40, 1.67)	-0.38 (-2.04, 1.28)	0.41 (-0.73, 1.54)	0.79 (-1.04, 2.63)
	No	5	0.12 (-1.02, 1.26)	-0.11 (-2.83, 2.60)	0.03 (-1.33, 1.40)	-0.23 (-1.92, 1.45)	-0.08 (-2.45, 2.28)	0.15 (-3.79, 4.09)
Providing meals	All	4	14.1 (2.0, 26.1)	-2.03 (-4.17, 0.11)	-6.12 (-12.6, 0.38)	-16.1 (-30.2, -2.0)	-20.2 (-38.6, -1.74)	-4.09 (-8.67, 0.49)
	Partial	7	-0.01 (-0.50, 0.49)	-0.19 (-0.93, 0.56)	0.15 (-0.57, 0.87)	-0.18 (-1.27, 0.91)	0.16 (-0.60, 0.92)	0.34 (-0.71, 1.39)
Blinding	Yes	7	0.25 (-0.33, 0.83)	-0.54 (-0.83, -0.25)	0.21 (-0.42, 0.83)	-0.79 (-1.49, -0.09)	-0.04 (-0.82, 0.74)	0.75 (0.07, 1.43)
	No	4	0.79 (-0.69, 2.27)	-2.34 (-4.76, 0.08)	26.9 (2.06, 51.8)	-3.13 (-6.51, 0.25)	26.1 (1.78, 50.5)	29.3 (2.05, 56.5)
2hr insulin, pmol/L								
All		11	1.91 (-19.3, 23.1)	-20.3 (-32.2, -8.45)	-24.9 (-53.9, 4.13)	-22.2 (-49.1, 4.58)	-26.8 (-72.5, 18.9)	-4.57 (-33.3, 24.2)
Region	US/Canada	3	Not estimated‡					
	Europe	8	1.60 (-20.2, 23.4)	-21.2 (-35.6, -6.9)	-23.7 (-54.7, 7.36)	-22.8 (-52.5, 6.83)	-25.3 (-72.9, 22.3)	-2.47 (-32.0, 27.1)
	Asia	0	Not estimated‡					
Age	≥52 years	7	-7.85 (-49.3, 33.6)	-1.84 (-32.2, 28.5)	8.76 (-42.7, 60.2)	6.01 (-51.7, 63.7)	16.6 (-47.7, 80.9)	10.6 (-34.4, 55.6)
	<52 years	4	Not estimated‡					
Sex	Men	4	Not estimated‡					
	Women	7	5.04 (-30.9, 41.0)	-35.5 (-75.6, 4.63)	22.7 (-68.2, 113)	-40.5 (-113, 32.0)	17.6 (-56.9, 92.2)	58.1 (-69.1, 185)
Body mass index	≥28 kg/m^2	7	0.80 (-20.7, 22.3)	-13.6 (-33.9, 6.76)	-22.8 (-54.8, 9.19)	-14.4 (-45.7, 17.0)	-23.6 (-69.2, 22.1)	-9.23 (-44.4, 25.9)
	<28 kg/m^2	4	Not estimated‡					
Diabetes	Yes	6	-3.2 (-42, 36)	7.13 (-58.5, 72.8)	-0.97 (-59.0, 57.1)	10.3 (-77.3, 97.9)	2.19 (-67.3, 71.7)	-8.10 (-98.9, 82.7)
	No	5	1.20 (-102, 104)	-25.6 (-150, 99.3)	-21.3 (-123, 80.2)	-26.8 (-252, 198)	-22.5 (-107, 62.3)	4.24 (-211, 220)
Providing meals	All	5	116 (-67.7, 300)	19.7 (-32.2, 71.6)	-81.6 (-209, 45.6)	-96.6 (-253, 59.8)	-198 (-459, 63.6)	-101 (-238, 35.1)
	Partial	6	-6.8 (-46.6, 32.9)	-8.36 (-52.4, 35.6)	-17.5 (-63.4, 28.3)	-1.56 (-54.1, 51.0)	-10.7 (-63.2, 41.7)	-9.17 (-50.5, 32.1)
Blinding	Yes	8	-2.1 (-23.8, 19.6)	-23.2 (-37.1, -9.28)	-14.6 (-49.5, 20.4)	-21.1 (-50.8, 8.6)	-12.5 (-64.2, 39.3)	8.62 (-25.6, 42.8)
	No	3	Not estimated‡					

S3 Table. Effects of isocalorically exchanging 5% of dietary energy between carbohydrates and major types of fatty acids on glucose-insulin metrics, with stratification by country, age, sex, diabetes status, provision of meals, and blinding in randomised controlled feeding trials.*

Outcome	Strata of trials*	N trials	Effects (95% confidence interval) of isocaloric replacement of 5% dietary energy					
			CHO →SFA	CHO →MUFA	CHO →PUFA	SFA →MUFA	SFA →PUFA	MUFA →PUFA
Si, 10^{-5}/(pmol/L)/min								
All		13	-0.10 (-0.21, 0.02)	-0.01 (-0.11, 0.08)	0.14 (-0.14, 0.43)	0.08 (-0.01, 0.17)	0.24 (-0.13, 0.61)	0.16 (-0.20, 0.52)
Region	US/Canada	5	Not estimated‡					
	Europe	8	0.00 (-0.21, 0.20)	-0.14 (-0.38, 0.10)	0.75 (0.18, 1.32)	-0.13 (-0.29, 0.03)	0.75 (0.08, 1.43)	0.89 (0.19, 1.58)
	Asia	0	Not estimated‡					
Age	≥52 years	5	0.27 (-0.30, 0.84)	0.27 (-0.57, 1.11)	0.25 (-0.96, 1.45)	0.00 (-0.32, 0.33)	-0.02 (-1.66, 1.62)	-0.03 (-1.90, 1.85)
	<52 years	8	-0.19 (-0.31, -0.07)	-0.05 (-0.16, 0.05)	0.27 (-0.01, 0.56)	0.13 (0.05, 0.22)	0.46 (0.09, 0.83)	0.33 (-0.03, 0.68)
Sex	Men	7	Not estimated‡					
	Women	6	Not estimated‡					
Body mass index	≥28 kg/m^2	7	0.14 (-0.01, 0.29)	0.12 (-0.02, 0.26)	0.19 (-0.41, 0.79)	-0.02 (-0.16, 0.13)	0.05 (-0.58, 0.67)	0.06 (-0.56, 0.69)
	<28 kg/m^2	6	-0.46 (-0.83, -0.09)	-0.41 (-0.93, 0.11)	0.51 (-0.85, 1.87)	0.05 (-0.14, 0.24)	0.96 (-0.44, 2.37)	0.92 (-0.54, 2.38)
Diabetes	Yes	0	Not estimated‡					
	No	13	-0.10 (-0.21, 0.02)	-0.01 (-0.11, 0.08)	0.14 (-0.14, 0.43)	0.08 (0.00, 0.17)	0.24 (-0.13, 0.61)	0.16 (-0.20, 0.52)
Providing meals	All	4	Not estimated‡					
	Partial	9	0.02 (-0.12, 0.16)	-0.07 (-0.17, 0.03)	0.70 (0.14, 1.26)	-0.09 (-0.22, 0.04)	0.67 (0.04, 1.31)	0.77 (0.15, 1.38)
Blinding	Yes	6	0.21 (-0.05, 0.46)	0.19 (-0.17, 0.56)	0.57 (0.05, 1.08)	-0.01 (-0.22, 0.20)	0.36 (-0.18, 0.90)	0.37 (-0.22, 0.96)
	No	7	0.08 (-0.43, 0.58)	0.20 (-0.28, 0.68)	-0.18 (-1.38, 1.01)	0.12 (0.03, 0.21)	-0.26 (-1.89, 1.38)	-0.38 (-2.00, 1.25)
AIR, pmol/L/min								
All		10	-0.02 (-0.11, 0.07)	-0.03 (-0.07, 0.01)	0.49 (0.17, 0.80)	-0.01 (-0.08, 0.06)	0.51 (0.20, 0.82)	0.52 (0.21, 0.82)
Region	US/Canada	3	Not estimated‡					
	Europe	7	-0.05 (-0.41, 0.31)	0.01 (-0.26, 0.28)	0.70 (0.06, 1.33)	0.07 (-0.11, 0.24)	0.75 (-0.16, 1.67)	0.69 (-0.14, 1.51)
	Asia	0	Not estimated‡					
Age	≥52 years	4	Not estimated‡					
	<52 years	6	-0.05 (-0.17, 0.08)	-0.05 (-0.10, 0.01)	0.37 (-0.14, 0.87)	0.00 (-0.09, 0.09)	0.42 (-0.07, 0.90)	0.41 (-0.06, 0.89)
Sex	Men	7	0.07 (-0.27, 0.41)	0.16 (-0.29, 0.61)	-0.25 (-1.45, 0.94)	0.09 (-0.06, 0.25)	-0.32 (-1.72, 1.08)	-0.41 (-1.89, 1.06)
	Women	3	Not estimated‡					
Body mass index	≥28 kg/m^2	5	Not estimated‡					
	<28 kg/m^2	5	Not estimated‡					
Diabetes	Yes	0	Not estimated‡					
	No	10	-0.02 (-0.11, 0.07)	-0.03 (-0.07, 0.01)	0.49 (0.17, 0.80)	-0.01 (-0.08, 0.06)	0.51 (0.20, 0.82)	0.52 (0.21, 0.82)
Providing meals	All	3	Not estimated‡					
	Partial	7	0.02 (-0.08, 0.13)	-0.03 (-0.08, 0.01)	0.57 (0.14, 1.00)	-0.06 (-0.15, 0.03)	0.55 (0.11, 0.98)	0.60 (0.18, 1.03)
Blinding	Yes	3	Not estimated‡					
	No	7	-0.08 (-0.26, 0.10)	-0.07 (-0.21, 0.07)	0.40 (-0.27, 1.06)	0.01 (-0.08, 0.10)	0.48 (-0.25, 1.21)	0.47 (-0.26, 1.20)

* Abbreviations: CHO, carbohydrates; SFA, saturated fatty acids; MUFA, monounsaturated fatty acids; PUFA, polyunsaturated fatty acids; IS, insulin sensitivity. Results for C-peptide were not shown, because of too few trials for stratified meta-regression (n trials=6 in total). Values represent the pooled estimated mean change (95% confidence interval) according to isocaloric exchange of two macronutrients with other macronutrient intakes held constant. The models adjusted for factors varying between within-trial arms, including intakes of protein and trans-fat (% energy) and dietary fiber (g/1000 kcal). 1 mg/dL=0.056 mmol/L for glucose; 1 μIU/mL=6 pmol/L; HbA1mmol/mol=(HbA1c % - 2.15)×10.929. See reference list in the Supplementary Information (S3 Text).

† Significant heterogeneity with α=0.05 level corrected for false-discovery rate inflated by multiple tests for strata and substitution effects.

‡ Not fitted because of a limited number of trials, yielding failure of convergence or unrealistic estimates.
