# Supplementary material for: Effects of Saturated Fat, Polyunsaturated Fat, Monounsaturated Fat, and Carbohydrate on Glucose-Insulin Homeostasis: A Systematic Review and Meta-analysis of Randomised Controlled Feeding Trials
Source: PLoS Med. 2016 Jul 19;13(7):e1002087. doi: 10.1371/journal.pmed.1002087 (PMC4951141; doi:10.1371/journal.pmed.1002087)
Supplement: S4 Table — (PDF) [file pmed.1002087.s006.pdf]

S4 Table. Effects of isocalorically exchanging 5% of dietary energy between carbohydrates and major types of fatty acids on glucose-insulin metrics: fixed-effects and random-effects meta-analyses by region, diabetes status, provision of meals, and blinding in randomised controlled feeding trials.*

Outcome and strata of trials†	N trials	Model‡	Effects (95% confidence interval) of isocaloric replacement of 5% dietary energy					
			CHO →SFA	CHO →MUFA	CHO →PUFA	SFA →MUFA	SFA →PUFA	MUFA →PUFA
Glucose, mnol/L								
US, Canada, or Europe	89	Fixed	0.01 (-0.01, 0.03)	0.00 (-0.02, 0.02)	-0.03 (-0.07, -0.00)*	-0.01 (-0.03, 0.02)	-0.04 (-0.07, -0.01)**	-0.04 (-0.08, 0.00)
		Random	0.02 (-0.03, 0.06)	0.01 (-0.03, 0.04)	-0.01 (-0.08, 0.05)	-0.01 (-0.06, 0.04)	-0.03 (-0.11, 0.04)	-0.02 (-0.10, 0.06)
Diabetes, yes	31	Fixed	-0.18 (-0.43, 0.08)	-0.14 (-0.22, -0.06)**	-0.32 (-0.50, -0.14)**	0.03 (-0.26, 0.33)	-0.15 (-0.48, 0.19)	-0.18 (-0.39, 0.03)
		Random	-0.01 (-0.18, 0.16)	-0.17 (-0.25, -0.08)***	-0.02 (-0.35, 0.32)	-0.16 (-0.35, 0.03)	-0.01 (-0.39, 0.37)	0.15 (-0.20, 0.50)
Diabetes, no	68	Fixed	0.03 (0.00, 0.05)	0.01 (-0.01, 0.03)	-0.01 (-0.04, 0.02)	-0.02 (-0.04, 0.00)	-0.04 (-0.07, 0.00)	-0.02 (-0.05, 0.02)
		Random	0.05 (0.01, 0.09)	0.02 (-0.02, 0.06)	0.02 (-0.02, 0.06)	-0.03 (-0.08, 0.02)	-0.03 (-0.07, 0.01)	0.00 (-0.06, 0.06)
Providing meals fully	53	Fixed	0.04 (0.00, 0.09)	-0.02 (-0.05, 0.01)	0.01 (-0.02, 0.05)	-0.07 (-0.10, -0.03)**	-0.03 (-0.09, 0.02)	0.04 (-0.01, 0.08)
		Random	0.07 (0.00, 0.15)*	-0.03 (-0.10, 0.05)	0.06 (-0.07, 0.19)	-0.10 (-0.20, -0.00)*	-0.01 (-0.13, 0.11)	0.09 (-0.08, 0.25)
Blinding	60	Fixed	0.01 (-0.04, 0.05)	-0.05 (-0.07, -0.02)***	-0.06 (-0.13, 0.01)	-0.05 (-0.10, -0.01)*	-0.07 (-0.14, 0.01)	-0.01 (-0.08, 0.05)
		Random	-0.05 (-0.17, 0.07)	-0.06 (-0.11, -0.01)*	-0.08 (-0.22, 0.06)	-0.01 (-0.13, 0.12)	-0.03 (-0.21, 0.15)	-0.02 (-0.17, 0.13)
HbA1c, %								
US, Canada, or Europe	22	Fixed	0.04 (-0.02, 0.09)	-0.08 (-0.12, -0.04)***	-0.11 (-0.17, -0.05)***	-0.11 (-0.17, -0.05)***	-0.14 (-0.22, -0.07)***	-0.03 (-0.09, 0.03)
		Random	0.04 (-0.06, 0.14)	-0.04 (-0.09, 0.01)	-0.04 (-0.13, 0.04)	-0.08 (-0.19, 0.03)	-0.09 (-0.21, 0.03)	-0.01 (-0.08, 0.07)
Diabetes, yes	18	Fixed	-0.11 (-0.29, 0.08)	-0.16 (-0.25, -0.06)**	-0.18 (-0.33, -0.03)*	-0.05 (-0.27, 0.17)	-0.08 (-0.26, 0.11)	-0.02 (-0.20, 0.15)
		Random	-0.17 (-0.41, 0.08)	-0.13 (-0.25, -0.01)*	-0.16 (-0.57, 0.26)	0.04 (-0.26, 0.33)	0.01 (-0.44, 0.46)	-0.03 (-0.48, 0.43)
Diabetes, no	5	Fixed	-0.02 (-0.20, 0.17)	-0.09 (-0.19, 0.01)	-0.11 (-0.20, -0.01)*	-0.07 (-0.18, 0.03)	-0.09 (-0.23, 0.05)	-0.02 (-0.08, 0.04)
		Random	Not estimated§					
Providing meals fully	13	Fixed	0.12 (0.03, 0.20)**	-0.02 (-0.08, 0.03)	-0.21 (-0.41, -0.02)*	-0.14 (-0.20, -0.07)*	-0.33 (-0.58, -0.07)*	-0.19 (-0.42, 0.04)
		Random	-0.06 (-0.48, 0.37)	-0.01 (-0.12, 0.09)	0.10 (-0.42, 0.62)	0.04 (-0.43, 0.51)	0.16 (-0.53, 0.84)	0.12 (-0.42, 0.65)
Blinding	16	Fixed	0.00 (-0.06, 0.06)	-0.10 (-0.15, -0.05)***	-0.15 (-0.28, -0.01)*	-0.11 (-0.17, -0.04)**	-0.15 (-0.32, 0.02)	-0.04 (-0.21, 0.12)
		Random	-0.04 (-0.32, 0.23)	-0.12 (-0.27, 0.03)	-0.05 (-0.51, 0.40)	-0.08 (-0.46, 0.31)	-0.01 (-0.41, 0.39)	0.07 (-0.45, 0.58)
Insulin, pmol/L								
US, Canada, or Europe	83	Fixed	-1.05 (-2.13, 0.02)	0.15 (-0.20, 0.50)	-1.29 (-2.31, -0.26)*	1.20 (0.14, 2.26)*	-0.23 (-2.05, 1.58)	-1.44 (-2.48, -0.39)*
		Random	-1.17 (-2.73, 0.40)	-0.08 (-0.57, 0.41)	-0.84 (-2.49, 0.81)	1.08 (-0.51, 2.68)	0.33 (-1.92, 2.57)	-0.76 (-2.48, 0.96)
Diabetes, yes	25	Fixed	-2.51 (-5.39, 0.37)	-1.96 (-3.67, -0.26)*	-3.33 (-5.37, -1.29)**	0.55 (-2.97, 4.08)	-0.82 (-4.47, 2.84)	-1.37 (-2.49, -0.24)
		Random	-2.84 (-6.58, 0.89)	-2.74 (-5.39, -0.09)*	-3.49 (-6.75, -0.23)*	0.11 (-4.84, 5.05)	-0.64 (-5.85, 4.56)	-0.75 (-3.76, 2.26)
Diabetes, no	65	Fixed	-0.96 (-1.56, -0.36)**	0.22 (-0.15, 0.59)	-1.23 (-3.03, 0.57)	1.18 (0.63, 1.73)***	-0.27 (-2.39, 1.85)	-1.45 (-3.33, 0.44)
		Random	-1.10 (-1.97, -0.22)*	0.06 (-0.72, 0.84)	0.86 (-2.19, 3.90)	1.16 (0.51, 1.80)***	1.95 (-1.16, 5.07)	0.80 (-2.35, 3.94)
Providing meals fully	46	Fixed	-1.36 (-2.25, -0.46)**	0.53 (-0.21, 1.27)	-2.51 (-3.88, -1.14)***	1.89 (1.07, 2.71)***	-1.15 (-2.70, 0.40)	-3.04 (-4.72, -1.36)***
		Random	-1.92 (-3.79, -0.04)*	-0.21 (-1.69, 1.27)	-0.95 (-4.11, 2.21)*	1.70 (-0.02, 3.43)	0.96 (-2.68, 4.60)	-0.74 (-4.50, 3.02)
Blinding	52	Fixed	-0.34 (-1.66, 0.98)	-0.14 (-0.58, 0.30)	-1.52 (-2.80, -0.24)*	0.21 (-1.12, 1.53)	-1.18 (-3.05, 0.69)	-1.39 (-2.65, -0.12)*
		Random	-1.24 (-3.40, 0.93)	-1.28 (-2.53, -0.03)*	-1.69 (-4.34, 0.96)	-0.04 (-2.47, 2.38)	-0.46 (-3.87, 2.96)	-0.41 (-3.44, 2.62)

S4 Table. Effects of isocalorically exchanging 5% of dietary energy between carbohydrates and major types of fatty acids on glucose-insulin metrics: fixed-effects and random-effects meta-analyses by region, diabetes status, provision of meals, and blinding in randomised controlled feeding trials.*

Outcome and strata of trials†	N trials	Model‡	Effects (95% confidence interval) of isocaloric replacement of 5% dietary energy					
			CHO →SFA	CHO →MUFA	CHO →PUFA	SFA →MUFA	SFA →PUFA	MUFA →PUFA
HOMA, %change								
US, Canada, or Europe	26	Fixed	1.4 (-1.0, 3.9)	-2.2 (-4.7, 0.5)	-3.0 (-5.7, -0.3)*	-3.5 (-7.1, 0.2)	-4.4 (-6.6, -2.0)***	-0.9 (-5.1, 3.5)
		Random	1.0 (-2.2, 4.2)	-2.4 (-6.5, 1.9)	-3.3 (-6.6, 0.0)	-3.3 (-8.4, 2.0)	-4.3 (-6.7, -1.8)***	-1.0 (-6.6, 5.0)
Diabetes, yes	6	Fixed	Not estimated§					
		Random						
Diabetes, no	24	Fixed	2.0 (-0.7, 4.8)	-1.3 (-3.9, 1.3)	-1.9 (-4.7, 1.0)	-3.3 (-6.5, 0.0)**	-3.9 (-6.3, -1.4)**	-0.6 (-4.6, 3.5)
		Random	2.9 (-0.7, 6.6)	-1.6 (-5.4, 2.4)	-1.5 (-5.0, 2.2)	-4.4 (-8.5, 0.0)**	-4.2 (-6.7, -1.7)**	0.1 (-4.9, 5.4)
Providing meals fully	15	Fixed	2.6 (-0.2, 5.4)	-3.9 (-6.8, -0.8)*	-1.4 (-4.5, 1.7)	-6.3 (-10.3, -2.1)*	-3.9 (-6.4, -1.3)*	2.5 (-2.5, 7.8)
		Random	2.5 (-0.5, 5.7)	-3.6 (-7.4, 0.4)	-1.6 (-4.9, 1.9)	-6.0 (-10.8, -0.9)*	-4.0 (-6.6, -1.3)**	2.1 (-3.9, 8.5)
Blinding	17	Fixed	-1.0 (-4.8, 3.0)	-5.6 (-8.7, -2.5)*	-4.8 (-10.4, 1.1)	-4.7 (-8.7, -0.5)*	-3.9 (-9.1, 1.7)	0.9 (-5.8, 8.1)
		Random	-2.0 (-9.4, 6.1)	-5.9 (-9.6, -2.0)**	-6.4 (-17.0, 5.4)	-4.0 (-10.9, 3.4)	-4.6 (-15.2, 7.4)	-0.6 (-12.3, 12.7)
2h glucose, mmol/L								
US, Canada, or Europe	10	Fixed	0.29 (-0.23, 0.81)	-0.37 (-0.61, -0.13)**	0.18 (-0.34, 0.71)	-0.66 (-1.29, -0.03)*	-0.11 (-0.84, 0.61)	0.55 (-0.06, 1.16)
		Random	0.38 (-0.64, 1.39)	-0.13 (-0.58, 0.32)	0.16 (-0.52, 0.83)	-0.50 (-1.71, 0.70)	-0.22 (-1.51, 1.06)	0.28 (-0.49, 1.06)
Diabetes, yes	6	Fixed	0.23 (-0.43, 0.88)	-0.16 (-1.41, 1.10)	0.63 (-0.40, 1.67)	-0.38 (-2.04, 1.28)	0.41 (-0.73, 1.54)	0.79 (-1.04, 2.63)
		Random	0.34 (-2.77, 3.46)	-0.53 (-4.05, 3.00)	1.10 (-1.81, 4.02)	-0.87 (-6.75, 5.01)	0.76 (-3.56, 5.08)	1.63 (-4.26, 7.52)
Diabetes, no	5	Fixed	Not estimated§					
		Random						
Providing meals fully	4	Fixed	Not estimated§					
		Random						
Blinding	7	Fixed	0.25 (-0.33, 0.83)	-0.54 (-0.83, -0.25)***	0.21 (-0.42, 0.83)	-0.79 (-1.49, -0.09)*	-0.04 (-0.82, 0.74)	0.75 (0.07, 1.43)*
		Random	0.29 (-0.41, 0.98)	-0.52 (-0.85, -0.20)*	0.21 (-0.46, 0.87)	-0.81 (-1.62, 0.00)	-0.08 (-0.97, 0.81)	0.73 (-0.01, 1.47)
2hr insulin, pmol/L								
US, Canada, or Europe	11	Fixed	1.8 (-19.4, 22.9)	-20.7 (-32.7, -8.7)***	-24.2 (-54.3, 5.9)	-22.5 (-49.5, 4.6)	-25.9 (-72.8, 20.9)	-3.5 (-32.5, 25.6)
		Random	-2.0 (-26.6, 22.6)	-19.1 (-33.0, -5.1)**	-17.8 (-50.6, 14.9)	-17.1 (-48.7, 14.6)	-15.8 (-65.6, 33.9)	1.2 (-29.5, 31.9)
Diabetes, yes	6	Fixed	-3.2 (-42.3, 35.9)	7.1 (-58.5, 72.8)	-1.0 (-59.0, 57.1)	10.3 (-77.3, 97.9)	2.2 (-67.3, 71.7)	-8.1 (-98.9, 82.7)
		Random	Not estimated§					
Diabetes, no	5	Fixed	Not estimated§					
		Random						
Providing meals fully	5	Fixed	Not estimated§					
		Random						
Blinding	8	Fixed	-2.1 (-23.8, 19.6)	-23.2 (-37.1, -9.3)**	-14.6 (-49.5, 20.4)	-21.1 (-50.8, 8.6)	-12.5 (-64.2, 39.3)	8.6 (-25.6, 42.8)
		Random	-3.4 (-26.0, 19.3)	-21.2 (-36.8, -5.5)**	-8.8 (-45.9, 28.3)	-17.8 (-49.1, 13.5)	-5.5 (-59.0, 48.0)	12.3 (-23.5, 48.2)

S4 Table. Effects of isocalorically exchanging 5% of dietary energy between carbohydrates and major types of fatty acids on glucose-insulin metrics: fixed-effects and random-effects meta-analyses by region, diabetes status, provision of meals, and blinding in randomised controlled feeding trials.*

Outcome and strata of trials†	N trials	Model‡	Effects (95% confidence interval) of isocaloric replacement of 5% dietary energy					
			CHO →SFA	CHO →MUFA	CHO →PUFA	SFA →MUFA	SFA →PUFA	MUFA →PUFA
Si, 10^{-5}/(pmol/L)/min								
US, Canada, or Europe	13	Fixed	-0.10 (-0.21, 0.02)	-0.01 (-0.11, 0.08)	0.14 (-0.14, 0.43)	0.08 (-0.01, 0.17)	0.24 (-0.13, 0.61)	0.16 (-0.20, 0.52)
		Random	-0.05 (-0.24, 0.13)	0.01 (-0.12, 0.13)	0.20 (-0.21, 0.61)	0.06 (-0.10, 0.22)	0.25 (-0.29, 0.78)	0.19 (-0.30, 0.69)
Diabetes, yes	0	Fixed	Not estimated§					
		Random						
Diabetes, no	13	Fixed	-0.10 (-0.21, 0.02)	-0.01 (-0.11, 0.08)	0.14 (-0.14, 0.43)	0.08 (-0.01, 0.17)	0.24 (-0.13, 0.61)	0.16 (-0.20, 0.52)
		Random	-0.05 (-0.24, 0.13)	0.01 (-0.12, 0.13)	0.20 (-0.21, 0.61)	0.06 (-0.10, 0.22)	0.25 (-0.29, 0.78)	0.19 (-0.30, 0.69)
Providing meals fully	4	Fixed	Not estimated§					
		Random						
Blinding	6	Fixed	0.21 (-0.05, 0.46)	0.19 (-0.17, 0.56)	0.57 (0.05, 1.08)*	-0.01 (-0.22, 0.20)	0.36 (-0.18, 0.90)	0.37 (-0.22, 0.96)
		Random	0.24 (-0.04, 0.53)	0.29 (-0.12, 0.69)	0.44 (-0.21, 1.09)	0.04 (-0.18, 0.27)	0.20 (-0.46, 0.85)	0.15 (-0.55, 0.86)
AIR, pmol/L/min								
US, Canada, or Europe	10	Fixed	-0.02 (-0.11, 0.07)	-0.03 (-0.07, 0.01)	0.48 (0.17, 0.80)**	-0.01 (-0.08, 0.07)	0.51 (0.20, 0.82)**	0.52 (0.21, 0.82)**
		Random	-0.04 (-0.14, 0.07)	-0.03 (-0.09, 0.04)	0.51 (0.13, 0.89)**	0.01 (-0.07, 0.09)	0.55 (0.16, 0.93)**	0.53 (0.15, 0.92)**
Diabetes, yes	0	Fixed	Not estimated§					
		Random						
Diabetes, no	10	Fixed	-0.02 (-0.11, 0.07)	-0.03 (-0.07, 0.01)	0.48 (0.17, 0.80)**	-0.01 (-0.08, 0.07)	0.51 (0.20, 0.82)**	0.52 (0.21, 0.82)**
		Random	-0.04 (-0.14, 0.07)	-0.03 (-0.09, 0.04)	0.51 (0.13, 0.89)**	0.01 (-0.07, 0.09)	0.55 (0.16, 0.93)**	0.53 (0.15, 0.92)**
Providing meals fully	2	Fixed	Not estimated§					
		Random						
Blinding	3	Fixed	Not estimated§					
		Random						

* Abbreviations: CHO, carbohydrates; SFA, saturated fatty acids; MUFA, monounsaturated fatty acids; PUFA, polyunsaturated fatty acids; IS, insulin sensitivity. Results for C-peptide were not shown, because of too few trials for stratified meta-regression (n trials=6 in total). Values represent the pooled estimated mean change (95% confidence interval) according to isocaloric exchange of two macronutrients with other macronutrient intakes held constant. The models adjusted for factors varying between within-trial arms, including intakes of protein and trans-fat (% energy) and dietary fiber (g/1000 kcal). 1 mg/dL=0.056 mmol/L for glucose; 1 μIU/mL=6 pmol/L; HbA1mmol/mol=(HbA1c % - 2.15)×10.929. * $p<0.05$, ** $p<0.01$ and *** $p<0.001$ for the effect estimate.

† Strata were selected from significant sources of heterogeneity (Table S3).

‡ Fixed and random represent fixed-effects and random-effects meta-analysis. Results from fixed-effects models are available in S3 Table and presented as a comparison.

§ Not fitted because of a limited number of trials, yielding failure of convergence or unrealistic estimates.
