# Supplementary material for: Effects of Saturated Fat, Polyunsaturated Fat, Monounsaturated Fat, and Carbohydrate on Glucose-Insulin Homeostasis: A Systematic Review and Meta-analysis of Randomised Controlled Feeding Trials
Source: PLoS Med. 2016 Jul 19;13(7):e1002087. doi: 10.1371/journal.pmed.1002087 (PMC4951141; doi:10.1371/journal.pmed.1002087)
Supplement: S5 Table — (PDF) [file pmed.1002087.s007.pdf]

S5 Table. Effects of isocalorically exchanging 5% of dietary energy between carbohydrate and major dietary fats on fasting glucose, haemoglobin A1c, and fasting insulin: sensitivity meta-analysis concerning model covariates and study characteristics.*

Outcome and consideration	CHO →SFA	CHO →MUFA	CHO →PUFA	SFA →MUFA	SFA →PUFA	MUFA →PUFA
Fasting glucose, mmol/L						
Main estimates†	0.02 (-0.01, 0.04)	0.00 (-0.02, 0.02)	-0.02 (-0.05, 0.01)	-0.02 (-0.04, 0.00)	-0.04 (-0.07, -0.01)	-0.02 (-0.05, 0.01)
Covariates for adjustment ‡						
*t*FA, fibre	0.02 (0.00, 0.04)	0.00 (-0.02, 0.02)	-0.02 (-0.04, 0.01)	-0.02 (-0.04, 0.00)	-0.04 (-0.07, 0.00)	-0.02 (-0.05, 0.02)
Protein, fibre	0.02 (0.00, 0.05)	0.00 (-0.02, 0.02)	-0.01 (-0.04, 0.01)	-0.02 (-0.04, 0.00)	-0.04 (-0.07, 0.00)	-0.02 (-0.05, 0.02)
Protein, *t*FA	0.02 (0.00, 0.04)	0.00 (-0.01, 0.02)	-0.02 (-0.05, 0.01)	-0.02 (-0.04, 0.00)	-0.04 (-0.07, -0.01)	-0.02 (-0.05, 0.01)
*t*FA, fibre, protein, total energy‡	0.02 (-0.01, 0.04)	0.00 (-0.02, 0.02)	-0.02 (-0.05, 0.01)	-0.02 (-0.04, 0.00)	-0.04 (-0.07, -0.01)	-0.02 (-0.05, 0.01)
*t*FA, fibre, protein, Δweight ‡	0.02 (0.00, 0.04)	0.00 (-0.02, 0.02)	-0.02 (-0.05, 0.00)	-0.02 (-0.04, 0.00)	-0.04 (-0.07, -0.01)	-0.02 (-0.06, 0.01)
Imputation of *t*FA §						
Ratio of *t*FA to MUFA	0.02 (0.00, 0.04)	0.01 (-0.02, 0.03)	-0.02 (-0.04, 0.01)	-0.01 (-0.04, 0.01)	-0.04 (-0.07, 0.00)	-0.02 (-0.06, 0.01)
Ratio of *t*FA to PUFA+MUFA	0.02 (-0.01, 0.04)	0.00 (-0.02, 0.03)	-0.02 (-0.04, 0.01)	-0.01 (-0.04, 0.01)	-0.03 (-0.07, 0.00)	-0.02 (-0.05, 0.01)
Ratio of *t*FA to total fat	0.02 (0.00, 0.04)	0.00 (-0.02, 0.02)	-0.02 (-0.04, 0.01)	-0.02 (-0.04, 0.00)	-0.04 (-0.07, -0.01)	-0.02 (-0.05, 0.01)
Trial subtypes (N of 99 trials) ‖						
Duration, ≥28 days (39)	0.00 (-0.03, 0.02)	-0.01 (-0.03, 0.02)	-0.02 (-0.07, 0.03)	-0.01 (-0.03, 0.01)	-0.02 (-0.07, 0.04)	-0.01 (-0.07, 0.05)
Caloric restriction trials (18)	0.00 (-0.13, 0.13)	0.00 (-0.05, 0.05)	-0.10 (-0.21, 0.02)	0.00 (-0.16, 0.16)	-0.09 (-0.31, 0.12)	-0.09 (-0.23, 0.04)
Aimed at varying SFA (30)	0.04 (-0.01, 0.09)	0.02 (-0.02, 0.07)	0.00 (-0.06, 0.06)	-0.02 (-0.07, 0.03)	-0.04 (-0.08, 0.00)	-0.02 (-0.09, 0.05)
Aimed at varying MUFA (48)	-0.02 (-0.06, 0.01)	-0.02 (-0.05, 0.00)	-0.04 (-0.09, 0.00)	0.00 (-0.03, 0.04)	-0.02 (-0.06, 0.03)	-0.02 (-0.06, 0.02)
Aimed at varying PUFA (29)	0.05 (-0.02, 0.12)	-0.04 (-0.08, 0.00)	-0.01 (-0.07, 0.04)	-0.09 (-0.15, -0.04)	-0.07 (-0.11, -0.02)	0.02 (-0.02, 0.07)
Haemoglobin A1c, %						
Main estimates†	0.03 (-0.02, 0.09)	-0.09 (-0.12, -0.05)	-0.11 (-0.17, -0.05)	-0.12 (-0.19, -0.05)	-0.15 (-0.23, -0.06)	-0.03 (-0.09, 0.03)
Covariates for adjustment ‡						
*t*FA, fibre	0.07 (0.01, 0.12)	-0.09 (-0.13, -0.05)	-0.15 (-0.22, -0.09)	-0.16 (-0.22, -0.09)	-0.22 (-0.31, -0.13)	-0.06 (-0.12, 0.00)
Protein, fibre	0.04 (-0.02, 0.09)	-0.07 (-0.11, -0.04)	-0.07 (-0.12, -0.03)	-0.11 (-0.18, -0.04)	-0.11 (-0.18, -0.03)	0.00 (-0.05, 0.05)
Protein, *t*FA	0.09 (0.04, 0.13)	-0.06 (-0.10, -0.02)	-0.10 (-0.16, -0.03)	-0.14 (-0.21, -0.08)	-0.18 (-0.26, -0.10)	-0.04 (-0.10, 0.02)
*t*FA, fibre, protein, total energy‡	0.03 (-0.02, 0.09)	-0.08 (-0.12, -0.05)	-0.11 (-0.19, -0.03)	-0.12 (-0.19, -0.05)	-0.14 (-0.24, -0.05)	-0.03 (-0.10, 0.05)
*t*FA, fibre, protein, Δweight ‡	0.07 (0.00, 0.13)	-0.06 (-0.10, -0.01)	-0.08 (-0.14, -0.02)	-0.12 (-0.20, -0.05)	-0.15 (-0.23, -0.06)	-0.02 (-0.08, 0.04)
Imputation of *t*FA §						
Ratio of *t*FA to MUFA	0.04 (-0.02, 0.09)	-0.08 (-0.12, -0.03)	-0.09 (-0.14, -0.03)	-0.12 (-0.18, -0.05)	-0.12 (-0.21, -0.04)	-0.01 (-0.07, 0.05)
Ratio of *t*FA to PUFA+MUFA	0.04 (-0.02, 0.09)	-0.08 (-0.12, -0.03)	-0.09 (-0.14, -0.04)	-0.11 (-0.18, -0.04)	-0.12 (-0.20, -0.04)	-0.01 (-0.07, 0.05)
Ratio of *t*FA to total fat	0.05 (-0.02, 0.11)	-0.08 (-0.12, -0.04)	-0.09 (-0.14, -0.03)	-0.12 (-0.20, -0.05)	-0.13 (-0.22, -0.04)	-0.01 (-0.06, 0.05)
Trial subtypes (N of 23 trials) ‖						
Duration, ≥28 days (8)	0.10 (-0.06, 0.25)	-0.26 (-0.39, -0.12)	0.14 (-0.13, 0.41)	-0.35 (-0.59, -0.11)	0.05 (-0.27, 0.36)	0.40 (0.04, 0.76)
Caloric restriction trials (3)	Not estimable					
Aimed at varying SFA (4)	Not estimable					
Aimed at varying MUFA (14)	0.09 (0.02, 0.17)	-0.04 (-0.09, 0.02)	-0.07 (-0.19, 0.05)	-0.13 (-0.19, -0.07)	-0.16 (-0.28, -0.04)	-0.03 (-0.13, 0.07)
Aimed at varying PUFA (8)	0.01 (-0.35, 0.38)	0.01 (-0.29, 0.31)	0.00 (-0.32, 0.33)	-0.01 (-0.26, 0.24)	-0.01 (-0.26, 0.23)	-0.01 (-0.07, 0.06)

(Continued)

S5 Table. Effects of isocalorically exchanging 5% of dietary energy between carbohydrate and major dietary fats on fasting glucose, haemoglobin A1c, and fasting insulin: sensitivity meta-analysis concerning model covariates and study characteristics.*

Outcome and consideration	CHO →SFA	CHO →MUFA	CHO →PUFA	SFA →MUFA	SFA →PUFA	MUFA →PUFA
Fasting insulin, pmol/L						
Main estimates†	-1.12 (-1.72, -0.53)	0.05 (-0.26, 0.36)	-1.60 (-2.77, -0.42)	1.17 (0.57, 1.78)	-0.47 (-2.01, 1.07)	-1.65 (-2.84, -0.45)
Covariates for adjustment ‡						
*t*FA, fibre	-1.14 (-1.73, -0.54)	0.00 (-0.31, 0.30)	-1.53 (-2.70, -0.36)	1.14 (0.54, 1.73)	-0.39 (-1.92, 1.14)	-1.53 (-2.69, -0.37)
Protein, fibre	-1.11 (-1.69, -0.52)	0.06 (-0.23, 0.36)	-1.56 (-2.66, -0.46)	1.17 (0.57, 1.77)	-0.45 (-1.97, 1.07)	-1.62 (-2.79, -0.46)
Protein, *t*FA	-1.14 (-1.65, -0.62)	0.04 (-0.22, 0.31)	-1.61 (-2.78, -0.44)	1.18 (0.66, 1.69)	-0.47 (-1.95, 1.01)	-1.65 (-2.87, -0.43)
*t*FA, fibre, protein, total energy‡	-1.12 (-1.73, -0.50)	0.06 (-0.37, 0.48)	-1.57 (-2.71, -0.43)	1.17 (0.57, 1.78)	-0.45 (-1.95, 1.05)	-1.62 (-2.85, -0.40)
*t*FA, fibre, protein, Δweight ‡	-0.99 (-1.62, -0.36)	0.11 (-0.34, 0.56)	-1.18 (-2.56, 0.20)	1.09 (0.49, 1.69)	-0.19 (-1.89, 1.50)	-1.29 (-2.67, 0.10)
Imputation of *t*FA §						
Ratio of *t*FA to MUFA	-1.12 (-1.72, -0.52)	0.10 (-0.24, 0.44)	-1.61 (-2.73, -0.50)	1.22 (0.58, 1.86)	-0.49 (-2.02, 1.03)	-1.72 (-2.92, -0.51)
Ratio of *t*FA to PUFA+MUFA	-1.12 (-1.71, -0.54)	0.10 (-0.23, 0.43)	-1.69 (-2.87, -0.50)	1.22 (0.62, 1.83)	-0.56 (-2.15, 1.02)	-1.79 (-3.07, -0.51)
Ratio of *t*FA to total fat	-1.14 (-1.73, -0.56)	0.08 (-0.24, 0.40)	-1.64 (-2.79, -0.49)	1.22 (0.61, 1.84)	-0.50 (-2.05, 1.05)	-1.72 (-2.94, -0.51)
Trial subtypes (N of 90 trials) ||						
Duration, ≥28 days (35)	-1.66 (-2.56, -0.76)	-1.05 (-2.20, 0.10)	-1.57 (-3.31, 0.17)	0.61 (-0.27, 1.49)	0.09 (-1.98, 2.15)	-0.52 (-2.81, 1.77)
Caloric restriction trials (18)	0.23 (-3.83, 4.29)	-0.22 (-2.52, 2.07)	-1.65 (-5.98, 2.68)	-0.45 (-6.09, 5.19)	-1.88 (-8.93, 5.18)	-1.43 (-7.00, 4.15)
Aimed at varying SFA (28)	-1.81 (-3.71, 0.09)	0.05 (-1.54, 1.65)	-2.73 (-4.54, -0.92)	1.86 (0.77, 2.96)	-0.92 (-3.48, 1.64)	-2.79 (-5.12, -0.45)
Aimed at varying MUFA (40)	-1.72 (-2.91, -0.52)	0.07 (-0.78, 0.93)	-0.86 (-2.11, 0.39)	1.79 (0.73, 2.85)	0.86 (-0.59, 2.30)	-0.93 (-2.00, 0.13)
Aimed at varying PUFA (25)	-1.80 (-4.11, 0.51)	-1.19 (-2.73, 0.35)	-2.97 (-4.51, -1.42)	0.61 (-1.22, 2.44)	-1.16 (-3.04, 0.71)	-1.77 (-2.80, -0.75)

* Abbreviations: CHO, carbohydrates; FA, fatty acids; SFA, saturated fatty acids; MUFA, monounsaturated fatty acids; tFA, trans fatty acisd; PUFA, polyunsaturated fatty acids. Values represent the pooled estimated mean change (95% confidence interval) according to isocaloric exchange of two macronutrients with other macronutrient intakes held constant. Effects of replacing CHO with SFA, MUFA, and PUFA by 5% energy amount were estimated by fixed-effects meta-regression, and resulting coefficients were then used for replacement (MUFA-SFA and PUFA-SFA). 1 mg/dL=0.056 mmol/L for glucose, 1 μIU/mL=6 pmol/L, HbA1mmol/mol=(HbA1c % - 2.15)×10.929.

† Adjusted for intakes of protein, trans-fat, and fibre that varied within trials. Fibre was used to adjust for CHO quality. Trans-fat and protein were adjusted for to allow estimation independent of these macronutrients. For trials without information on trans-fat, intake was imputed by using SFA, MUFA, PUFA, and trial aims (test for trans-fat, yes/no) (see supplementary materials).

‡ Models used two of protein, trans-fat, and fibre for statistical adjustment, rather than three of these. Additional adjustment was made for total energy intake and arm-specific weight change during an intervention (Δweight).

§ Imputation was applied to 213 individual arms of 70 trials from which information on trans-fat was not reported. In the main analysis, regression-based approach was undertaken. In the sensitivity analyses, a ratio of trans-fat to MUFA or MUFA+PUFA or total fat was tested.

|| Meta-analysis was repeated by limiting trials by duration of intervention, implementation of caloric restriction, and specific aims. Analyses based on trials specifically varying certain exposure are generally recommended in multiple-treatment meta-analysis (Salanti et al., Stat Methods Med Res, 2008, Ref. 21 of the main manuscript). Not estimable, when the number of trials were too few.
