# Supplementary material for: Effects of Saturated Fat, Polyunsaturated Fat, Monounsaturated Fat, and Carbohydrate on Glucose-Insulin Homeostasis: A Systematic Review and Meta-analysis of Randomised Controlled Feeding Trials
Source: PLoS Med. 2016 Jul 19;13(7):e1002087. doi: 10.1371/journal.pmed.1002087 (PMC4951141; doi:10.1371/journal.pmed.1002087)
Supplement: S1 Text — (PDF) [file pmed.1002087.s008.pdf]

# S1 Text. Protocol of A Systematic Review and Meta-Analysis of Effects of Macronutrient Replacement on Glucose-Insulin Homeostasis

**Title:** Effects of isocaloric replacement of major macronutrients on glucose homeostasis: systematic reviews and meta-analysis of randomized controlled feeding trials

**Authors:** **Fumiaki Imamura, Renata E. Micha, Jason H. Wu, Marcia C. de Oliveira Otto, Fadar O. Otite, Ajibola I. Abioye, Dariush Mozaffarian**

**Corresponding Author:**
Fumiaki Imamura, fimamura@hsph.harvard.edu
Harvard School of Public Health, Department of Epidemiology
677 Huntington Ave., Kresge 913C
Tel: (617)432-7518, Fax: (617)566-7805

**Start date:** Dec., 2010
**Funding sources/sponsors:** None
**Conflicts of interest:** None

## Aims and Methods

**Objectives**: to evaluate effects of isocaloric replacement of major macronutrients intake, focusing on different types of fatty acids, on fasting glucose, fasting insulin and insulin resistance.

**Eligibility Criteria:**

1) Randomized controlled feeding trials of isocaloric exchange of different types of dietary fat, total carbohydrate and total protein
2) Reporting different types of dietary fat intake and examining post-intervention values or changes in the values of fasting glucose, fasting insulin or measures of insulin resistance as effects of dietary modification on glucose homeostasis.

**Exclusion Criteria:**

1) Studies of concomitant intervention limiting comparability of isocaloric exchange of major macronutrients
   i) Interventions with other major dietary difference beside macronutrient, such as vegan diet, dietary fiber and others
   ii) Trials with uneven intervention of weight-loss or other dietary or lifestyle factors
2) Intervention of dietary advice only, whereas studies partially providing meals or foods will be included
3) Examining acute effects of foods, such as postprandial effects of the tested meals
4) Examining effects of meals, such as breakfast and evening snacks.
5) Observational or non-randomized studies
6) Pregnant women or children (aged <18 years)
7) Commentaries or reviews or case-reports
8) Duplicate publications from the same study, queried to consider results from additional analyses (e.g. stratification)

**Participants/population:** Adults age 18 years or older, non-pregnant. No specific criteria for disease conditions

**Interventions/exposures:** Isocaloric exchange of macronutrients of:
- saturated fatty acids
- monounsaturated fatty acids
- polyunsaturated fatty acids (PUFA)
- carbohydrates

**Primary outcomes:** Absolute changes in fasting glucose, fasting insulin and measures of insulin resistance (IR). We will consider the following measures, listed in the order of priority for aggregation:
- Any indices from hyperinsulinemic euglycemic glucose clamp
- Any indices from hyperglycemic clamp

* Difference in duration and rates of glucose/insulin infusion, accounting for body weight, fat free mass or energy expenditure, difference in time points of glucose/insulin assessments will be assessed in secondary analyses.
* Correlations in changes between different measures were described in previous studies[1-5]

**Secondary outcomes:** Measure of glycated hemoglobin (hemoglobin A1c, HbA1c)
Measures of β-cell dysfunction. Similar to IR, variety of indices are expected and aggregation will be attempted after standardization. In the order of priority, we will consider the following measures:

- Any indices from hyperglycemic clamp
- Any indices from Indices from Continuous infusion of glucose with model assessment (CIGMA)
- Any indices from Acute Insulin Response (AIR) from IVGTT
- Corrected insulin response, insulinogenic index from OGTT

Arginine infusion test and replacing insulin to pro-insulin, pro-insulin/insulin ratio or C-peptide for each index will be analyzed if feasible in regards to the number of trials.

**Potential sources of heterogeneity:**

- Study design
    - Parallel or Cross-over[6]
    - Duration of intervention
    - Difference in measures of outcomes for IR and β-cell function
- Carbohydrate intake, gram/day, when isocaloric exchange of macronutrients other than carbohydrate
- Carbohydrate quality (dietary fiber, glycemic index)
- Weight-loss or weight-stable trial
- Obesity status
- Prevalent diseases
    - Healthy
    - Prediabetics (impaired glucose tolerance or impaired fasting glucose)
    - Type 2 diabetes (duration of the condition, medication or levels of glycated proteins)
- Demographic status
    - Age
    - Sex
    - Country/Location
- Overall quality score
- Imputation of index using reported measures of fasting glucose and insulin
- Imputation of group level change (SE), using reported group means

*For continuous variables, whether treated as categorical or continuous will be judged, depending on data availability

**Quality assessment:** Jadad scale will be used to assess a quality of randomized controlled trials (RCT)[7] and meta-regression analysis will be performed to assess the influence of the study quality.[8] Inter-rater reliability by duplicate reviews will be assessed. In addition, influences of bias will be assessed for each score component.[9, 10]

The following 11 items will be considered[7], where two of them (#1 and #9) are specified as inclusion criteria and will not be considered to calculate overall score:

1. Was the study described as randomized?
2. Was the study described as double-blind?
3. Was there a description of withdrawals and drop outs?
4. Were the objectives of the study defined?
5. Were the outcome measures defined clearly?
6. Was there a clear description of the inclusion and exclusion criteria?
7. Was the sample size justified (e.g., power calculation)?
8. Was there a clear description of the interventions?
9. Was there at least one control (comparison) group?
10. Was the method used to assess adverse effects described?
11. Were the methods of statistical analysis described?

**Data extraction:** Ten databases will be used (see Search section). Three researchers (FI, RM and JW) will be involved in screening and duplicate reviews for data extraction.

Missing covariates of each study will be collected by direct contacts to a corresponding author. If missing covariates are unavailable, information will be coded as missing and used as an indicator variable in analyses.

**Strategy for data synthesis:**

1) Hierarchical meta-regression analysis will be performed as previous reviews of different topics.[11-13]
2) Studies examining changes in multiple indices of insulin resistance or β-cell dysfunction are anticipated.[14-18] We will obtain a summary measure of effect by aggregating outcome measures extracted from each study.

**Analysis of heterogeneity and bias:**

1) Heterogeneity across prespecified factors will be assessed by multivariate meta-regression analyses and by stratified analyses adjusting for available covariates.
2) Publication bias will be assessed by visual inspection of a funnel plot of an estimated effect as a function of standard error and by statistical tests of Begg's test and Egger's test.[19, 20] Capture-recapture method will also be considered to assess potential publication bias.[20] Sensitivity analysis will be performed by trim-and-fill method.[19-21]
3) Publication bias will be considered statistically significant if $p<0.1$.[22] Heterogeneity will be considered statistically significant if $p<0.05$, for which we regarded potential risk of under-power and also false-positive due to multiple testing for the prespecified factors.

**Reporting**

PRISMA statement will be followed.[23]

<u>**Information to be queried**</u>

**• Publication Information**
Study ID
Author
Author's email
Year of publication
Funding source

**• Study Design**
Study objectives
Years (dates) study was performed
Study location
Study design (parallel or cross-over)
Single center or multi-centers
Caloric restriction for weight loss
Physical activity for weight loss
Number of comparators
Wash-out period, if cross-over
Ward or not
Feeding duration
Population description (including inclusion, exclusion criteria)
Criteria of BMI or weight
Criteria of disease status
Criteria of age range
Criteria of early censoring
Sample-size justification

**• Recruitment results**
Total N of participants
Baseline age, mean
Baseline age, range or SD
Men, %
Race/ethnicity
White, %
African American, %
Hispanic, %
Asia, %
Baseline BMI mean
Baseline BMI range or SD
Glycated protein, mean
Glycated protein, range or SD
Glycated protein, description
% of type 1 diabetes
Duration of diabetes
% of type 2 diabetes
Duration of diabetes
% of antidiabetic medication
% of drop-out

**• Intervention**
Isocaloric comparison of supplement or not
Total energy intake, kcal/day
total carbohydrate, % of energy
total fat, % of energy
protein, % of energy
saturated fatty acids, % of energy
monounsaturated fatty acids, % of energy
polyunsaturated fatty acids, % of energy
n-6 polyunsaturated fatty acids, % of energy
n-3 polyunsaturated fatty acids, % of energy
n-3 polyunsaturated fatty acids, gram/day
Trans fatty acids, % of energy

**• Outcome assessments**

Fasting glucose, baseline mean
Fasting glucose, baseline SD
Fasting glucose, change
Fasting glucose, change SE
Fasting glucose, change 95% CI
Fasting glucose, change p-value
Fasting insulin, baseline mean
Fasting insulin, baseline SD
Fasting insulin, change
Fasting insulin, change SE
Fasting insulin, change 95% CI
Fasting insulin, change p-value
HOMA-IR, baseline mean
HOMA-IR, baseline SD
HOMA-IR, change
HOMA-IR, change SE
HOMA-IR, change 95% CI
HOMA-IR, change p-value
HOMA-IR, imputed or not
Clamp test, index or marker, description
Clamp test, index or marker, baseline
Clamp test, index or marker, baseline SD
Clamp test, index or marker, change
Clamp test, index or marker, change SE
Clamp test, index or marker, change 95% CI
Clamp test, index or marker, change p-value
Glycated hemoglobin, baseline
Glycated hemoglobin, baseline SD
Glycated hemoglobin, change
Glycated hemoglobin, change SE
Glycated hemoglobin, change 95% CI
Glycated hemoglobin, change p-value

**• Other covariates**
Carbohydrate intake
Glycemic index
Glycemic load
Dietary fiber intake
Diabetes med change
BMI or body weight change

**• Study Quality**
Blinding for outcome assessment
Blinding for exposure
Jadad Score

1. Was the study described as randomized?
2. Was the study described as double-blind?
3. Was there a description of withdrawals and drop outs?
4. Were the objectives of the study defined?
5. Were the outcome measures defined clearly?
6. Was there a clear description of the inclusion and exclusion criteria?
7. Was the sample size justified (e.g., power calculation)?
8. Was there a clear description of the interventions?
9. Was there at least one control (comparison) group?
10. Was the method used to assess adverse effects described?
11. Were the methods of statistical analysis described?

Sum

**Notes**
Author contacted to provide additional data (yes, no)
Additional data provided (yes, no)
Written permission to acknowledge obtained (yes, no)
*Followed by note and log information about contacts with authors.

**Search strategy**

**Searches:** 10 electronic databases

1. PUBMED
2. EMBASE
3. Agris, Amed, HMIC, PsycINFO (2, 3 & 4 can be searched simultaneously through the OVID interface – remove duplicates)
4. WEB OF KNOWLEDGE
   - BIOSIS
   - WEB OF SCIENCE
   - CAB abstracts
5. CINAHL
6. The Cochrane library

7. Grey literature sources (SIGLE; system for information on grey literature in Europe, British library inside database, and dissertation abstracts online)
8. Faculty of 1000

**Exposure terms**

| | | |
|---|---|---|
| unsaturated | trans fatty | high-carbohydrate |
| monounsaturated | trans-fat | low-carbohydrate |
| polyunsaturated | trans fat | high-protein |
| omega-6 | trans unsaturated | low-protein |
| omega-3 | conjugated linoleic | Isocaloric |
| n-6 | high-fat | oil |
| n-3 | low-fat | oils |

***edible oils (seed oil, safflower oil, sunflower oil, corn oil, sesame oil, soybean oil, soyabean oil, rapeseed oil, canola oil, olive oil, nut oil, linseed oil, grapeseed oil, peanut oil, avocado oil, palm oil, vegetable oil, margarine, fish oil).

**Outcome terms**

| | | |
|---|---|---|
| Insulin | Hyperglycemic | Hemoglobin A1C |
| Glucose | Hyperinsulinemic | C-peptide |
| Euglycemic | Minimal model | Proinsulin |

**Study design terms**

| | | |
|---|---|---|
| randomized | intervention | ward |
| trial | interventions | |
| trials | feeding | |

**Limits if available**

| | |
|---|---|
| Humans | Rnadomized controlled trials |
| Adults (≥18 years) | Neither editorials, commentaries nor reviews |

**References**


**1.** Alvarez JA, Bush NC, Hunter GR, Brock DW, Gower BA. Ethnicity and weight status affect the accuracy of proxy indices of insulin sensitivity. *Obesity (Silver Spring).* Dec 2008;16(12):2739-2744.

**2.** Vazquez LA, Pazos F, Berrazueta JR, et al. Effects of changes in body weight and insulin resistance on inflammation and endothelial function in morbid obesity after bariatric surgery. *J Clin Endocrinol Metab.* Jan 2005;90(1):316-322.

**3.** Kim HJ, Kim SK, Shim WS, et al. Rosiglitazone improves insulin sensitivity with increased serum leptin levels in patients with type 2 diabetes mellitus. *Diabetes Res Clin Pract.* Jul 2008;81(1):42-49.

**4.** Haffner SM, Greenberg AS, Weston WM, Chen H, Williams K, Freed MI. Effect of rosiglitazone treatment on nontraditional markers of cardiovascular disease in patients with type 2 diabetes mellitus. *Circulation.* Aug 6 2002;106(6):679-684.

**5.** Riserus U, Basu S, Jovinge S, Fredrikson GN, Arnlov J, Vessby B. Supplementation with conjugated linoleic acid causes isomer-dependent oxidative stress and elevated C-reactive protein: a potential link to fatty acid-induced insulin resistance. *Circulation.* Oct 8 2002;106(15):1925-1929.

**6.** Curtin F, Elbourne D, Altman DG. Meta-analysis combining parallel and cross-over clinical trials. III: The issue of carry-over. *Stat Med.* Aug 15 2002;21(15):2161-2173.

**7.** Jadad AR, Moore RA, Carroll D, et al. Assessing the quality of reports of randomized clinical trials: Is blinding necessary? *Controlled Clinical Trials.* 1996;17(1):1-12.

**8.** Greenland S. Quality Scores Are Useless and Potentially Misleading: Reply to "Re: A Critical Look at Some Popular Analytic Methods". *American Journal of Epidemiology.* August 1, 1994 1994;140(3):300-301.

**9.** Juni P, Witschi A, Bloch R, Egger M. The Hazards of Scoring the Quality of Clinical Trials for Meta-analysis. *JAMA.* September 15, 1999 1999;282(11):1054-1060.

**10.** Olivo SA, Macedo LG, Gadotti IC, Fuentes J, Stanton T, Magee DJ. Scales to Assess the Quality of Randomized Controlled Trials: A Systematic Review. *Physical Therapy.* February 2008 2008;88(2):156-175.

**11.** Frost C, Clarke R, Beacon H. Use of hierarchical models for meta-analysis: experience in the metabolic ward studies of diet and blood cholesterol. *Stat Med.* Jul 15 1999;18(13):1657-1676.

**12.** Clarke R, Frost C, Collins R, Appleby P, Peto R. Dietary lipids and blood cholesterol: quantitative meta-analysis of metabolic ward studies. *BMJ.* January 11, 1997 1997;314(7074):112.

**13.** Mozaffarian D, Clarke R. Quantitative effects on cardiovascular risk factors and coronary heart disease risk of replacing partially hydrogenated vegetable oils with other fats and oils. *Eur J Clin Nutr.* 2009;63(S2):S22-S33.

**14.** McClenaghan NH. Determining the relationship between dietary carbohydrate intake and insulin resistance. *Nutr Res Rev.* Dec 2005;18(2):222-240.

**15.** Wallace TM, Levy JC, Matthews DR. Use and Abuse of HOMA Modeling. *Diabetes Care.* June 2004 2004;27(6):1487-1495.

**16.** Wallace TM, Matthews DR. The assessment of insulin resistance in man. *Diabet Med.* Jul 2002;19(7):527-534.
**17.** Ferrannini E, Mari A. How to measure insulin sensitivity. *J Hypertens.* Jul 1998;16(7):895-906.
**18.** Scheen AJ, Paquot N, Castillo MJ, Lefebvre PJ. How to measure insulin action in vivo. *Diabetes Metab Rev.* Jul 1994;10(2):151-188.
**19.** Macaskill P, Walter SD, Irwig L. A comparison of methods to detect publication bias in meta-analysis. *Stat Med.* Feb 28 2001;20(4):641-654.
**20.** Bennett DA, Latham NK, Stretton C, Anderson CS. Capture-recapture is a potentially useful method for assessing publication bias. *J Clin Epidemiol.* Apr 2004;57(4):349-357.
**21.** Duval S, Tweedie R. Trim and fill: A simple funnel-plot-based method of testing and adjusting for publication bias in meta-analysis. *Biometrics.* Jun 2000;56(2):455-463.
**22.** Petitti DB. Approaches to heterogeneity in meta-analysis. *Stat Med.* Dec 15 2001;20(23):3625-3633.
**23.** Liberati A, Altman DG, Tetzlaff J, et al. The PRISMA statement for reporting systematic reviews and meta-analyses of studies that evaluate health care interventions: explanation and elaboration. *Ann Intern Med.* Aug 18 2009;151(4):W65-94.
