# Supplementary material for: Effects of Saturated Fat, Polyunsaturated Fat, Monounsaturated Fat, and Carbohydrate on Glucose-Insulin Homeostasis: A Systematic Review and Meta-analysis of Randomised Controlled Feeding Trials
Source: PLoS Med. 2016 Jul 19;13(7):e1002087. doi: 10.1371/journal.pmed.1002087 (PMC4951141; doi:10.1371/journal.pmed.1002087)
Supplement: S2 Text — (PDF) [file pmed.1002087.s009.pdf]

S2 Text. PRISMA 2009 Checklist

PRISMA 2009 Checklist

Section/topic	#	Checklist item	Reported on page #
**TITLE**			
Title	1	Identify the report as a systematic review, meta-analysis, or both.	On the first page, written as “Effects of Saturated, Polyunsaturated, and Monounsaturated Fat on Glucose-Insulin Homeostasis: a Systematic Review and Meta-Analysis of 102 Randomized Controlled Feeding Trials"
**ABSTRACT**			
Structured summary	2	Provide a structured summary including, as applicable: background; objectives; data sources; study eligibility criteria, participants, and interventions; study appraisal and synthesis methods; results; limitations; conclusions and implications of key findings; systematic review registration number.	Structured in compliance with the instruction of PLOS Medicine, including three subheadings: Background, Methods and Findings, and Conclusions. Key elements are included, as instructed. This review was not registered to a public space.
**INTRODUCTION**			
Rationale	3	Describe the rationale for the review in the context of what is already known.	In the Introduction section, availability of previous studies, but their limitations and importance to address them are described.
Objectives	4	Provide an explicit statement of questions being addressed with reference to participants, interventions, comparisons, outcomes, and study design (PICOS).	In the Introduction section, participants (adults), interventions (dietary macronutrients), comparisons (mutual, between macronutrients), and outcome (glucose-insulin homeostasis)
**METHODS**			
Protocol and registration	5	Indicate if a review protocol exists, if and where it can be accessed (e.g., Web address), and, if available, provide registration information including registration number.	S1 Text, included as one of supplementary materials
Eligibility criteria	6	Specify study characteristics (e.g., PICOS, length of follow-up) and report characteristics (e.g., years considered, language, publication status) used as criteria for eligibility, giving rationale.	The first paragraph of the Methods section. Fig 1 and S3 Text. Eligibility is written in details including the items of PICOS. Fig 1 includes the reasons for exclusion of trials along with the number of trials excluded after full-text review.

Information sources	7	Describe all information sources (e.g., databases with dates of coverage, contact with study authors to identify additional studies) in the search and date last searched.	The second sub-section of the Methods section, S3 Text<br>S3 Text include the full details of the databases and access dates.
Search	8	Present full electronic search strategy for at least one database, including any limits used, such that it could be repeated.	S3 Text includes the suggested information. Systematic searches were done three times to repeat update. Text S2 includes the first one, sufficing needs for replication.
Study selection	9	State the process for selecting studies (i.e., screening, eligibility, included in systematic review, and, if applicable, included in the meta-analysis).	The first paragraph of the Methods section. Fig 1, S3 Text<br>Fig 1 has the fow diagram of study selections.
Data collection process	10	Describe method of data extraction from reports (e.g., piloted forms, independently, in duplicate) and any processes for obtaining and confirming data from investigators.	The second subsection of the Methods section. S3 Text<br>Independent duplicate reviews and data extraction are also noted in the Methods section (“Data extraction”). S3 Text includes extra information related to author correspondence
Data items	11	List and define all variables for which data were sought (e.g., PICOS, funding sources) and any assumptions and simplifications made.	”Meta-analysis” section in the Methods section, S3 Text.<br>Main effects terms and covariates for statistical adjustment and for assessment of heterogeneity are documented in the main text and in the supplementary information (including protocol, S2 Text). Data preparation needed for covariates are also documented in the S3 Text.
Risk of bias in individual studies	12	Describe methods used for assessing risk of bias of individual studies (including specification of whether this was done at the study or outcome level), and how this information is to be used in any data synthesis.	The last two subsections of the Methods section, S2 Table.<br>Use of the scale developed by Jadad et al (ref #14) is stated on ”Data extraction” section in the Methods section. The footnote of the S2 Table also includes the description of the assessment of trials evaluated. S2 Table includes the all results of the assessment.<br>Assessment of heterogeneity by study design was also considered as the assessment of bias, because of the important to examine influence of potential bias, rather than qualitative assessment (Ref #21).
Summary measures	13	State the principal summary measures (e.g., risk ratio, difference in means).	”Meta-analysis” section of the Methods section, S3 Text

			The method section includes the estimation of the effects of iso-caloric exchange between 5% of energy from different types of macronutrients on one of measures of glucose-insulin homeostasis. S3 Text includes more details including the formulation of a meta-regression model.
Synthesis of results	14	Describe the methods of handling data and combining results of studies, if done, including measures of consistency (e.g., I^2) for each meta-analysis.	”Meta-analysis” section of the Methods section, S3 Text<br>Methods for use of fixed-effects meta-analysis are described with the rationale of using the approach. Measures of consistency were not obtained in this study, while random-effects were performed as one of sensitivity analyses.
Risk of bias across studies	15	Specify any assessment of risk of bias that may affect the cumulative evidence (e.g., publication bias, selective reporting within studies).	The last section in the Methods section<br>Assessment of small-study bias is described as an approach extended from a standard method. Assessment of heterogeneity by pre-specified factors potentially related to bias is described as well as a part of sensitivity analysis.
Additional analyses	16	Describe methods of additional analyses (e.g., sensitivity or subgroup analyses, meta-regression), if done, indicating which were pre-specified.	The last section of the methods section<br>Assessment of heterogeneity, a number of sensitivity analyses, and multiple imputation were described. When not pre-specified, post hoc decision is noted. The appended protocol can also be read to indicate which analyses were pre-specified.
**RESULTS**			
Study selection	17	Give numbers of studies screened, assessed for eligibility, and included in the review, with reasons for exclusions at each stage, ideally with a flow diagram.	The first paragraph of the Results section, Fig 1<br>The items are all covered. The flow diagram is presented (Fig 1).
Study characteristics	18	For each study, present characteristics for which data were extracted (e.g., study size, PICOS, follow-up period) and provide the citations.	Fig 1, S1 Table, S2 Table<br>There items are fully available in S1 Table and S2 Table, presented for each trial. Citation numbers are included in the table as well, and the full list is available in S3 Text (noted in the Table footnote).
Risk of bias within studies	19	Present data on risk of bias of each study and, if available, any outcome level assessment (see item 12).	S2 Table<br>Each of 11 components for the Jadad scale is presented in the S2 Table.
Results of individual	20	For all outcomes considered (benefits or harms), present, for	Table 2, Fig 2

studies		each study: (a) simple summary data for each intervention group (b) effect estimates and confidence intervals, ideally with a forest plot.	Summary estimates are available in Table 2 and also described in text in the Results section. A standard forest plot is not available. Forest plots were not applicable to our multiple-treatment meta-analysis, because single studies did not provide estimates under modelled assumption that all other intervention effects were fixed.
Synthesis of results	21	Present results of each meta-analysis done, including confidence intervals and measures of consistency.	Individual sub-sections in the Results section, Fig 2, S1 Fig, S2 Fig, S3 Table, S4 Table<br>Presenting all results derived from assessment for heterogeneity, sensitivity analyses accounting for different assumptions or senarios, and assessment for publication bias were presented.
Risk of bias across studies	22	Present results of any assessment of risk of bias across studies (see Item 15).	”Sensitivity analyses and small study bias” in the Methods section, Fig S2<br>are including text and results from assessment of publication bias and assessment of stability of the results against potential sources of bias.
Additional analysis	23	Give results of additional analyses, if done (e.g., sensitivity or subgroup analyses, meta-regression [see Item 16]).	“Exploration of Heterogeneity” and ”Sensitivity analyses and small study bias” in the Methods section,, Fig 2, S3 Table, S4 Table, S5 Table, S1 Fig, and Fig S2<br>presenting results from sensitivity analysis and assessment for heterogeneity.
**DISCUSSION**			
Summary of evidence	24	Summarize the main findings including the strength of evidence for each main outcome; consider their relevance to key groups (e.g., healthcare providers, users, and policy makers).	The first paragraph of the Discussion section summarises the key findings of this study. The second to forth paragraphs include relevance of the findings to existing dietary guidelines and implications of the findings.
Limitations	25	Discuss limitations at study and outcome level (e.g., risk of bias), and at review-level (e.g., incomplete retrieval of identified research, reporting bias).	The second last paragraph of the Discussion section<br>Includes the text describing limitations of the study, discussing availability of published information, a statistical power, generalisability of the findings to diverse populations and to long-term effects of habitual diets, and possible publication bias.
Conclusions	26	Provide a general interpretation of the results in the context of other evidence, and implications for future research.	The paragraphs of the Discussion section except the first one, the last one and the second last one

			Biological evidence, clinical evidence, and epidemiological evidence are documented for different types of fatty acids to discuss consistency with available evidence and with existing public health policy. Accounting for totality of available evidence, needs for future research are also discussed.
**FUNDING**			
Funding	27	Describe sources of funding for the systematic review and other support (e.g., supply of data); role of funders for the systematic review.	Meta-data in the submission includes funding information, role of funders, and conflict of interest. The Acknowledgement section includes support from authors of publications.
