# Supplementary material for: Effects of Saturated Fat, Polyunsaturated Fat, Monounsaturated Fat, and Carbohydrate on Glucose-Insulin Homeostasis: A Systematic Review and Meta-analysis of Randomised Controlled Feeding Trials
Source: PLoS Med. 2016 Jul 19;13(7):e1002087. doi: 10.1371/journal.pmed.1002087 (PMC4951141; doi:10.1371/journal.pmed.1002087)
Supplement: S3 Text — (PDF) [file pmed.1002087.s010.pdf]

S3 Text. Eligibility criteria, literature search, data preparation, imputation, and reference list

[Eligibility criteria](#)........1
[Literature strategy](#)........2
[Data preparation](#)........4
[Imputation](#)........5
[References of Supplementary Information](#)........6

Eligibility criteria

The protocol is appended in S1 Text. The objective of this review was to quantify effects of isocaloric replacement of major macronutrients intake, focusing on different types of fatty acids, on fasting glucose, fasting insulin and insulin resistance. Eligibility criteria were 1) randomised controlled feeding trials of isocaloric exchange of different types of dietary fat, total carbohydrates and total protein; 2) trials reporting different types of dietary fat intake and examining post-intervention values or changes in the values of fasting glucose, fasting insulin or measures of insulin resistance as effects of dietary modification on glucose homeostasis; and 3) trials recruiting adults aged 18 years or older.

Studies were excluded if trials tested effects of interventions that limited comparability of isocaloric exchange of major macronutrients: trials testing interventions concomitant with macronutrient composition, such as dietary fibre intake, vegan diet, diet with high glycaemic index (GI), and others, and an uneven intervention of weight-loss or other dietary or lifestyle factors; trials testing interventions of dietary advice only, whereas trials partially providing meals or foods were included; interventions of a single meal to test acute effects of foods, such as postprandial effects of the tested meals or breakfast or evening snacks, rather than a habitual diet. We also excluded observational or non-randomized studies, studies recruiting pregnant women or children (aged <18 years), publications of commentaries or reviews or case-reports, and duplicate publications from the same. Duplicate publications identified were compiled as well to extract additional information if available.

Literature strategy

We identified eligible publications systematically using the following electronic databases: PUBMED; EMBASE; OVID (EMBASE, Agris, Amed, HMIC, PsycINFO); WEB OF KNOWLEDGE (BIOSIS, WEB OF SCIENCE, CAB abstracts); CINAHL; The Cochrane Library; Grey literature sources (SIGLE; system for information on grey literature in Europe, British library inside database, and dissertation abstracts online); and Faculty of 1000.

Search was conducted by using three sets of search terms: exposure terms, outcome terms, and study-design terms (see Text S2). At least a term of each of the three sets was specified to be present in search. If search restrictions for a study design and for a search field of a title and abstract were available, such restrictions were applied. Language and years of publications were not restricted. Search by subject headings or key words was included if available.

Electronic search was carried out on 17 December 2010. The search was updated on 18 February 2012 and on 26 November 2015. After confirming that five databases were enough to identify eligible trials, the latter two searches were carried out in five databases (n of additional articles, not included in the subsequent paragraphs): PubMed (n=346), OVID (n=363), EMBASE (n=99), Web of Knowledge (n=1548) and Cochrane Library (n=37). In each search engine, removal of duplicates was applied if available. Search results from the multiple databases were merged and the number of duplicates (Figure 1 of the main manuscript) was calculated.

In addition to the electronic search, we hand-searched eligible publications. For the hand search, we reviewed references of each publication identified and also published review articles [1–20] that were identified in electronic searches.

In PubMed, 605 articles were identified by the following search query: ("Fatty Acids, Omega-6"[MeSH] OR "Fatty Acids, Omega-3"[MeSH] OR "Fatty Acids, Unsaturated"[MeSH] OR "Fatty Acids, Monounsaturated"[MeSH] OR "Trans Fatty Acids"[MeSH] OR "monounsaturated"[tiab] OR "mono-unsaturated"[tiab] OR "unsaturated"[tiab] OR "polyunsaturated"[tiab] OR "saturated"[tiab] OR "trans-unsaturated"[tiab] OR "trans-fatty"[tiab] OR "trans fatty"[tiab] OR "trans unsaturated"[tiab] OR "trans fat"[tiab] OR "omega-6"[tiab] OR "omega-3"[tiab] OR "n-6"[tiab] OR "n-3"[tiab] OR "low-fat"[tiab] OR "high-fat"[tiab] OR "high-carbohydrate"[tiab] OR "low-

carbohydrate"[tiab] OR "high-protein"[tiab] OR "low-protein"[tiab] OR "isocaloric"[tiab] OR "palmitic"[tiab] OR "palmitate"[tiab] OR "stearic"[tiab] OR "stearate"[tiab] OR "myristic"[tiab] OR "myristate"[tiab] OR "lauric"[tiab] OR "laurate"[tiab] OR "SFA"[tiab] OR "oleic"[tiab] OR "oleate"[tiab] OR "palmitoleic"[tiab] OR "palmitoleate"[tiab] OR "MUFA"[tiab] OR "linoleic"[tiab] OR "linoleate"[tiab] OR "octadecadienoic acid"[tiab] OR "PUFA"[tiab] OR "vaccenic acid"[tiab] OR "vaccenate"[tiab] OR "conjugated linoleic"[tiab] OR "CLA"[tiab] OR "TFA"[tiab] OR "coconut oil"[tiab] OR "butter"[tiab] OR "lard"[tiab] OR "seed oil"[tiab] OR "safflower oil"[tiab] OR "sunflower oil"[tiab] OR "corn oil"[tiab] OR "sesame oil"[tiab] OR "soybean oil"[tiab] OR "soyabean oil"[tiab] OR "rapeseed oil"[tiab] OR "canola oil"[tiab] OR "olive oil"[tiab] OR "nut oil"[tiab] OR "linseed oil"[tiab] OR "grapeseed oil"[tiab] OR "peanut oil"[tiab] OR "avocado oil"[tiab] OR "palm oil"[tiab] OR "vegetable oil"[tiab] OR "margarine"[tiab] OR "hydrogenated oil"[tiab] OR "fish oil"[tiab]) AND ("Insulin resistance"[MeSH] OR "Glucose clamp technique"[MeSH] OR "glucose tolerance test"[MeSH] OR "Hemoglobin A, Glycosylated"[MeSH] OR "insulin"[tiab] OR "fasting serum glucose"[tiab] OR "fasting plasma glucose"[tiab] OR "fasting glucose"[tiab] OR "glucose tolerance"[tiab] OR "glucose clamp"[tiab] OR "euglycemic"[tiab] OR "hyperglycemic"[tiab] OR "hyperinsulinemic"[tiab] OR "minimal model"[tiab] OR "hemoglobin A1c"[tiab] OR "glycated hemoglobin"[tiab] OR "proinsulin"[tiab] OR "C-peptide"[tiab]) AND ("randomized"[tiab] OR "intervention"[tiab] OR "ward"[tiab] OR "feeding"[tiab] OR "trials"[tiab] OR "trial"[tiab] OR "supplements"[tiab] OR "supplement"[tiab] OR "supplementation"[tiab]) NOT ("Case-Control Studies"[MeSH] OR "Cohort Studies"[MeSH] OR "case-control"[tiab] OR "cohort"[tiab] OR "case-report"[tiab] OR "ad libitum"[tiab] OR "adolescents" OR "children" OR "gestational"[tiab] OR "pregnant"[tiab] OR "pregnancy"[tiab]) NOT ("rats"[tiab] OR "monkeys"[tiab] OR "primates"[tiab] OR "rabbits"[tiab] OR "cats"[tiab] OR "dogs"[tiab] OR "mice"[tiab] OR "pigs"[tiab] OR "cows"[tiab]) Limits:Humans, Clinical Trial, Randomized Controlled Trial, All Adult: 19+ years

In EMBASE, 369 articles were identified by the following search query: ((("monounsaturated" or "mono-unsaturated" or "unsaturated" or "polyunsaturated" or "saturated" or "trans fatty" or "trans unsaturated" or "trans fat" or "trans-unsaturated" or "trans-fatty" or "omega-6" or "omega-3" or "n-3" or "n-6" or "low-fat" or "high-fat" or "low-carbohydrate" or "high-carbohydrate" or "low-protein" or "high-protein" or "isocaloric" or "palmitic" or "palmitate" or "stearic" or "stearate" or "myristic" or "myristate" or "lauric" or "laurate" or "SFA" or "oleic" or "oleate" or "palmitoleic" or "palmitoleate" or "MUFA" or "linoleic" or "linoleate" or "octadecadienoic acid" or "PUFA" or "vaccenic acid" or " accinate" or "CLA" or "TFA" or "hydrogenated oil" or "coconut oil" or "butter" or "lard" or "seed oil" or "safflower oil" or "sunflower oil" or "corn oil" or "sesame oil" or "soybean oil" or "soyabean oil" or "rapeseed oil" or "canola oil" or "olive oil" or "nut oil" or "linseed oil" or "grapeseed oil" or "peanut oil" or "avocado oil" or "palm oil" or "vegetable oil" or "margarine" or "fish oil") and ("fasting glucose" or "fasting plasma glucose" or "fasting serum glucose" or "glucose tolerance" or "glucose clamp" or "insulin" or "euglycemic" or "hyperglycemic" or "hyperinsulinemic" or "minimal model" or "hemoglobin A1c" or "glycated hemoglobin" or "proinsulin" or "C-peptide") and ("trials" or "trial" or "randomized" or "feeding" or "ward" or "intervention" or "supplements" or "supplement" or "supplementation")) not ("rats" or "monkeys" or "primates" or "rabbits" or "cats" or "dogs" or "mice" or "pigs" or "cows") not ("case-control" or "cohort" or "case-report" or "ad libitum" or "adolescents" or "children" or "gestational" or "pregnant" or "pregnancy")):ti,ab; Limited to: controlled clinical trial from Evidence Based Medicine; randomized controlled trial from Evidence Based Medicine; article from Publication Types; article in press from Publication Types; conference abstract from Publication Types; conference paper from Publication Types;'adult from Age Groups; aged from Age Groups; humans

In OVID (combined search at OVID, AGRICOLA, AMED, EMBASE, and PsycINFO), 1380 articles were identified after removing duplicates within OVID. ((("monounsaturated" or "mono-unsaturated" or "unsaturated" or "polyunsaturated" or "saturated" or "trans fatty" or "trans unsaturated" or "trans fat" or "trans-unsaturated" or "trans-fatty" or "omega-6" or "omega-3" or "n-3" or "n-6" or "low-fat" or "high-fat" or "low-carbohydrate" or "high-carbohydrate" or "low-protein" or "high-protein" or "palmitic" or "palmitate" or "stearic" or "stearate" or "myristic" or "myristate" or "lauric" or "laurate" or "SFA" or "oleic" or "oleate" or "palmitoleic" or "palmitoleate" or "MUFA" or "linoleic" or "linoleate" or "octadecadienoic acid" or "PUFA" or "vaccenic acid" or "vaccinate" or "CLA" or "TFA" or "hydrogenated oil" or "coconut oil" or "butter" or "lard" or "seed oil" or "safflower oil" or "sunflower oil" or "corn oil" or "sesame oil" or "soybean oil" or "soyabean oil" or "rapeseed oil" or "canola oil" or "olive oil" or "nut oil" or "linseed oil" or "grapeseed oil" or "peanut oil" or "avocado oil" or "palm oil" or "vegetable oil" or "margarine" or "fish oil") and ("fasting glucose" or "fasting plasma glucose" or "fasting serum glucose" or "glucose tolerance" or "glucose clamp" or "insulin" or "euglycemic" or "hyperglycemic" or "hyperinsulinemic" or "minimal model" or "hemoglobin A1c" or "glycated hemoglobin" or "proinsulin" or "C-peptide") and ("trials" or "trial" or "randomized" or "cross-over") and ("diet" or "dietary" or "feeding" or "ward" or "supplements" or "supplement" or

"supplementation")) not ("cross-sectional" or "case-control" or "cohort" or "ecological" or "case-series" or "case-report" or "ad libitum" or "dietary advice" or "dietary monitoring" or "dietary recommendation" or "review" or "meta-analysis" or "statement" or "guideline" or "adolescents" or "children" or "gestational" or "pregnant" or "pregnancy" or "rats" or "monkeys" or "primates" or "rabbits" or "cats" or "dogs" or "mice" or "pigs" or "cows"));
Limits: keywords search in abstracts, human, age (18 or older)

In Web Of Science, the following queries were searched for three different databases: Web of Knowledge, BIOSIS, and CABI abstracts. We compiled 328 articles from Web-of-Knowledge, 240 from BIOSIS, and 183 from CABI.

Search queries and results in Web of Science database

# Query	Terms applied in electronic search	Web-of-Knowledge	BIOSIS	CABI
# 1	TS=("hydrogenated oil" OR "*nut oil" OR "butter" OR "lard" OR "*lower oil" OR "corn oil" OR "*bean oil" OR "*seed oil" OR "canola oil" OR "avocado oil" OR "palm oil" OR "sesame oil" OR "olive oil" OR "margarine" OR "fish oil" OR "n-3" OR "omega-3" OR "n-6" OR "omega-6" OR "*turated fat*" OR "trans fat*" OR "n-6" OR "omega-6" OR "low-fat" OR "high-fat" OR "isocaloric" OR "palmit*" OR "stear*" OR "myrist*" OR "laur*" OR "oleic" OR "SFA" OR "MUFA" OR "linole*" OR "octadecadienoic" OR "PUFA" OR "vaccen*" OR "CLA" OR "TFA")	>100,000	>100,000	>100,000
# 2	TS=("fasting glucose" OR "fasting serum glucose" OR "fasting plasma glucose" OR "glucose tolerance" OR "insulin" OR "glucose clamp" OR "euglycemic " OR "minimal model" OR "hyperglycemic" OR "hyperinsulinemic" OR "proinsulin" OR "C-peptide")	>100,000	>100,000	52741
# 3	TS=("trials" or "trial" or "randomized" or "feeding" or "ward" or "intervention" or "supplements" or "supplement" or "supplementation")	>100,000	>100,000	>100,000
# 4	#1 AND #2 AND #3	603	443	298
# 5	TI=("meta-analysis" or "review" or "case-control" or "cohort" or "case-report" or "ad libitum" or "adolescents" or "children" or "gestational" or "pregnant" or "pregnancy")	>100,000	>100,000	>100,000
# 6	TI=("rat*" or "monkey*" or "rabbit*" or "cat*" or "dog*" or "primate*" or "mouse" or "mice" or "pig*")	>100,000	>100,000	>100,000
# 7	#6 NOT #7 NOT #8<br>Refined by: [excluding] Document Type=(REVIEW)	330	241	185
# 8	Removing duplicates.	328	240	183

In CINAHL (EBSCOhost), 165 articles were identified by including the following search in title or abstract: ”((("monounsaturated" or "mono-unsaturated" or "unsaturated" or "polyunsaturated" or "saturated" or "trans fatty" or "trans unsaturated" or "trans fat" or "trans-unsaturated" or "trans-fatty" or "omega-6" or "omega-3" or "n-3" or "n-6" or "low-fat" or "high-fat" or "low-carbohydrate" or "high-carbohydrate" or "low-protein" or "high-protein" or "isocaloric" or "palmitic" or "palmitate" or "stearic" or "stearate" or "myristic" or "myristate" or "lauric" or "laurate" or "SFA" or "oleic" or "oleate" or "palmitoleic" or "palmitoleate" or "MUFA" or "linoleic" or "linoleate" or "octadecadienoic acid" or "PUFA" or "vaccenic acid" or " accinate" or "CLA" or "TFA" or "hydrogenated oil" or "coconut oil" or "butter" or "lard" or "seed oil" or "safflower oil" or "sunflower oil" or "corn oil" or "sesame oil" or "soybean oil" or "soyabean oil" or "rapeseed oil" or "canola oil" or "olive oil" or "nut oil" or "linseed oil" or "grapeseed oil" or "peanut oil" or "avocado oil" or "palm oil" or "vegetable oil" or "margarine" or "fish oil") and ("fasting glucose" or "fasting plasma glucose" or "fasting serum glucose" or "glucose tolerance" or "glucose clamp" or "insulin" or "euglycemic" or "hyperglycemic" or "hyperinsulinemic" or "minimal model" or "hemoglobin A1c" or "glycated hemoglobin" or "proinsulin" or "C-peptide") and ("trials" or "trial" or "randomized" or "feeding" or "ward" or "intervention" or "supplements" or "supplement" or "supplementation")) not ("rats" or "monkeys" or "primates" or "rabbits" or "cats" or "dogs" or "mice" or "pigs" or "cows") not ("case-control" or "cohort" or "case-report" or "ad libitum" or "adolescents" or "children" or "gestational" or "pregnant" or "pregnancy")); narrow by SubjectAge: - Aged, 80 and over, Aged: 65+ years, Adult: 19-44 years, Middle Aged: 45-64 years

In Cochrane Library, 760 articles were identified, after removing 10 within the databse for: "monounsaturated" or "mono-unsaturated" or "unsaturated" or "polyunsaturated" or "saturated" or "trans fatty" or "trans unsaturated" or "trans fat" or "trans-unsaturated" or "trans-fatty" or "omega-6" or "omega-3" or "n-3" or "n-6" or "low-fat" or "high-fat" or "low-carbohydrate" or "high-carbohydrate" or "low-protein" or "high-protein" or "isocaloric" or "palmitic" or "palmitate" or "stearic" or "stearate" or "myristic" or "myristate" or "lauric" or "laurate" or "SFA" or "oleic" or "oleate" or "palmitoleic" or "palmitoleate" or "MUFA" or "linoleic" or "linoleate" or "octadecadienoic acid" or "PUFA" or "vaccenic acid" or " accinate" or "CLA" or "TFA" or "hydrogenated oil" or "coconut oil" or "butter" or "lard" or "seed oil" or "safflower oil" or "sunflower oil" or "corn oil" or "sesame oil" or "soybean oil" or "soyabean oil" or "rapeseed oil" or "canola oil" or "olive oil" or "nut oil" or "linseed oil" or "grapeseed oil" or "peanut oil" or "avocado oil" or "palm oil" or "vegetable oil" or "margarine" or "fish oil" in Title, Abstract or Keywords and "fasting glucose" or "fasting plasma glucose" or "fasting serum glucose" or "glucose tolerance" or "glucose clamp" or "insulin" or "euglycemic" or "hyperglycemic" or "hyperinsulinemic" or "minimal model" or "hemoglobin A1c" or "glycated hemoglobin" or "proinsulin" or "C-peptide" in Title, Abstract or Keywords and "trials" or "trial" or "randomized" or "feeding" or "ward" or "intervention" or "supplements" or "supplement" or "supplementation" in Title, Abstract or Keywords not "Case-Control Studies" or "Cohort Studies" or "case-control" or "cohort" or "case-report" or "ad libitum" or "adolescents" or "children" or "gestational" or "pregnant" or "pregnancy" in Title, Abstract or Keywords not "rats" or "monkeys" or "primates" or "rabbits" or "cats" or "dogs" or "mice" or "pigs" or "cows" in Title, Abstract or Keywords in Cochrane Central Register of Controlled Trials".

In SIGLE, one article was identified in the search of ("fasting plasma glucose" OR "fasting serum glucose" OR "fasting glucose" OR "insulin" OR "glucose clamp" OR "euglycemic " OR "minimal model" OR "hyperglycemic" OR "hyperinsulinemic" OR "proinsulin" OR "C-peptide" OR "hemoglobin A1c" OR "glycated hemoglobin") AND ("n-3" OR "omega-3" OR "*turated fat*" OR "trans *fat*" OR "n-6" OR "omega-6" OR "low-fat" OR "high-fat" OR "isocaloric" OR "palmit*" OR "stear*" OR "myrist*" OR "laur*" OR "oleic" OR "SFA" OR "MUFA" OR "linole*" OR "octadecadienoic" OR "PUFA" OR "vaccen*" OR "CLA" OR "TFA" OR "hydrogenated oil" OR "*nut oil" OR "butter" OR "lard" OR "*lower oil" OR "corn oil" OR "*bean oil" OR "*seed oil" OR "canola oil" OR "avocado oil" OR "palm oil" OR "margarine" OR "fish oil" OR "*e oil"). In Faculty of 1000, no article was identified.

Data preparation

Demographics and key characteristics of trials are presented in S1 Table and S2 Table. Some trials presented results after stratification by demographics [21–24]. While the number of trials was unchanged, each stratum was analyzed as an independent trial. Some trials with more than 2 arms included non-comparable arms [25–27], for example, comparing four arms of high-fat high-GI diet, low-fat high-GI diet, high-fat low-GI diet, and low-fat low-GI diet. Each of these trials was considered having multiple independent trials: for example, one trial comparing between high-fat high-GI diet and low-fat high-GI; and one comparing between high-fat low-GI and low-fat low-GI.

Information for meta-analysis was not always reported in a publication. Missing information was replaced with information available in other publications from the same trial; obtained from authors after direct contact; imputed from available information (e.g. midpoints of a range if only a range was presented) [28]. Simple imputation included average ages not presented in three papers [29–31]. For Tardy *et al.* and Vidon et al, a midpoint of an age range was used. For Poppitt *et al.*, the information was taken from another publication from the same authors [32], of which the identifical sampling frame was confirmed. A prevalence of diabetes was not presented in Sundram et al. [33] and in Noakes *et al.* [34] Mean plus two standard deviations (SD) of fasting glucose was less than 7 mmol/L (126 mg/dL) and thus all adults in the two trials were considered as adulst without diabetes (prevalence=0). Only one trial recruited type 1 diabetes patients [35], considerd to be 100% prevalence of diabetes, as well as many other trials not clarifying patieitns with type 1 or type 2 diabetes. The other imputation with uncertainty is described in the next section in the subsequent subsection.

We obtained addition information by contacting authors [36–52]. In the current meta-regression model, percent energy from major macronutrients, percent energy from trans fat, and fibre were considered as required. For the assessment of heterogeneity, other covariate information was needed. If information on covariates was missing as well as the main exposures (major macronutrients) and outcomes (glycaemic outcomes), we contacted a corresponding author of the publication. If a publication indicated measurements of the selected outcomes but did not present the post-intervention values, we requested them (means and SD) of each trial arm. Other information we

requested included confirmation of a study design related to eligibility criteria (e.g. randomisation, provision of meals) or of key demographics of paricipants (mean age, body-mass index). If the eligibility criteria were not confirmed through contact, trials were excluded.

According to eligibility criteria, we excluded trials in which composition of macronutrients was not reported or not informative enough to compute the composition after we reviewed publications and contacted authors. In several publications, a ratio of PUFA to SFA or a similar metrics was available [53–58]. Using such information on dietary fat composition and % energy from total fat, we calculated % energy from SFA, MUFA, and PUFA. For Tardy *et al.*, imputation was performed with a minor assumption. The authors reported that 64.5% of energy from fat came from the test fat presented in the publication in details including compositions of each individual fatty acid [30]. The rest, a non-test fat, came from *trans*-free vegetable oil including rapeseed oil. For fat composition of the rapeseed oil was informed from the French food composition table (http://www.afssa.fr/TableCIQUAL/index.htm). Gannon *et al.* did not present macronutrient composition [45], but the information was available in the other publication from the same group [59]. Of the two publications by Bjermo *et al.* and Iggman *et al.*, the authors provided % energy of major fatty acids after we contacted the authors [48,51].

The outcome measures were often presented in a figure [22,26,31,33,39,43,44,60–68] that displayed blood concentrations of glucose, insulin, or other molecules, for example, over 120 or 180 minutes after intravenous infusion of glucose or an oral glucose tolerance test. For the graphical information, point estimates and error bars were digitalized to numeric information by two authors independently (FI and MCOO) with a publically available software (Dagra®, Blue Leaf Software Ltd., Hamilton, New Zealand), and two values for a single estimate were averaged.

Acute insulin response (AIR), a gold-standard measure of β-cell function, was used when area-under the curve of AIR to an intravenous glucose dose constant across trials (300 mg/kg) [27,39,66,69–72]. Insulin measurements at 6, 10, or 19 min of AIR after infusion were divided by the minute for standardisation of the outcome per min across trials: this treatment alter estimates numerically, but did not alter interpretation of results. Insulin sensitivity index was assessed with a comparable manner across trials [22,39,53,66,68–72] with glucose infusion rate of 300 mg/kg and a slight difference in frequency in blood sampling up to 3 hours (-5 or -15 min to 180 min).

Imputation

Missing information was imputed systematically. Uncertainty attributable to use of random values was accounted for by undertaking a multiple imputation as described in the last paragraph in this sub-section. We imputed post-intervention values if only p-values were reported so that p-values could be reproduced. For example, post-inetrvention values with p-values >0.05 were imputed to produce p>0.05; otherwise, we considered, publication bias would have been expected by excluding non-significant results, but including signfiicant effects.

If standard deviations (SDs) of post-intervention values were missing, but ones at baseline were available, the balisene SDs were used under assumption that the variability between individuals did not change. If SD still remained missing, SD were imputed by using available SD in different trials and demographic information. The imputation of SD was assumed not to influence our results as demonstrated elsewhere [73,74].

In cross-over trials, within-trial covariance should be accounted for because outcome values are correlated to the degree of within-individual correlations [75]. The information was not reported in any pubilcations, but calculable by using reported information [76]. Thus, we calculated within-individual correlations for outcomes we evaluated [22,34,36,43,46,49–51,54,61,64,69,70,77–96]. For the cross-over trials without the information, *r* were imputed by modelling a multiple-outcome hierarchical regression with covariates of age, sex (% men), diabetes status, sites (US or Canada, Europe, or other areas), and durations of interventions.

Intakes of fibre and trans fat were included as covariates in our analysis. Caloric restriction, body-mass index (BMI), and weight change over time were included in assessment of heterogeneity. The influences of caloric restiction and of weight change were evaluated in sensitivity analysis. Information on these variables was not always available. Trans fat was important as a covariate (see the next paragraph), but available only in nineteen trials. Thus, for trials without information on trans fat, we imputed the values by considering biological plausibility. To impute the information on the other variables, a regression-based approach was taken for fibre intake [24,30,33,43,48,50,97–

101], total caloric intake [24,43,102–104], BMI [31], and weight change [21,25,26,31,43,55,57,61,65,69,72,85,88,92,96,103–109]. These variables to impute were modelled simultaneously in sequential equations that included predictors of age, sex, caloric restriction (yes or no), diabetes status, and sites. The process was undertaken in two steps: first to obtain trial means of the continuous variables; and then to obtain within-trial variability around the means.

Trans fat is one of the energy-contributing nutrient, providing typically <2% of total energy intake in a population. To estimate effects of isocaloric replacement between major macronutrients, meta-analysis of feeding trials need to adjust for trans fat that can vary within a trial, although this was not done in macronutrient meta-analysis [110,111]. Of 100 trials, 16 trials (46 arms) provided the estimates of trans-fat intakes [21,30,31,33,37,40,50,70,71,85,91,96,97,107,109,112–115]. Using the available information, we performed regression-based imputation by using relationships between trans-fat, SFA, MUFA, PUFA, and whether or not a trial aimed to test a trans fat. Before imputation, trans fat was log-transformed, so that regression met the assumption of normal distribution of the imputed variable and that imputed values could not be zero or negative. Additionally, as three alternative imputations, three ratios of trans-fat to MUFA, to MUFA+PUFA, to SFA+MUFA+PUFA in the 19 trials were used to impute trans-fat intakes, for which different considerations for conversions of unsaturated fatty acids to trans fat and to saturated fat. As presented in S4 Table, sensitivity analyses were performed to assess if results differed by these approaches. Results did not vary substantially by the approach, and regression-based imputation tended to give conservative results overall.

We recognised the uncertainty of imputation by using a pseudo-random number function. To account for the uncertainty, we conducted multiple imputation by generating ten datasets based on ten different imputation seeds. Ten independent estimates were pooled to obtain the main estimate which precision was based on both within-imputation and between-imputation variance estimates [116]. This decision was made post hoc as we observed the between-imputation variability appeared to be not always trivial, ranging from 0.1% to 40% of the total variability (between-imputation variability + within-imputation variability). Thus, the estimation procedure was undertaken in the all meta-analyses in this study, including the main analyses, stratified analysis, and sensitivity analysis.

The uncertainty in estimating within-trial correlation r was additionally examined by fixing r to be a single value across all non-parallel trials. We repeated meta-analyses nineteen times: in each repeat, a between-arm correlation of each non-parallel trial was fixed to have a single value from 0.05 to 0.95 with an increment of 0.05. The results are presented in S1 Fig. We found little evidence for the influence of r if we consider the reasonable values around r=0.6. The figure also indicates that, if r=0 had been modelled, powers of crossover trials would have been lost, indicating importance of taking the approach modelling r in analysis.

References of Supplementary Information

1. Daly ME, Vale C, Walker M, Alberti KG, Mathers JC. Dietary carbohydrates and insulin sensitivity: a review of the evidence and clinical implications. Am J Clin Nutr. 1997;66(5):1072–85.
2. Lichtenstein AH, Schwab US. Relationship of dietary fat to glucose metabolism. Atherosclerosis. 2000;150(2):227–43.
3. Vessby B. Dietary fat and insulin action in humans. Br J Nutr. 2000;83 Suppl 1:S91–6.
4. Montori VM, Farmer A, Wollan PC, Dinneen SF. Fish oil supplementation in type 2 diabetes: a quantitative systematic review. Diabetes Care. 2000;23(9):1407–15.
5. Shekelle PG, Morton SC, MacLean CH, Garland RH, Tu W, Jungvig LK, et al. Health Effects of Omega-3 Fatty Acids on Lipids and Glycemic Control in Type II Diabetes and the Metabolic Syndrome and on Inflammatory Bowel Disease, Rheumatoid Arthritis, Renal Disease, Systemic Lupus Erythematosus and Osteoporosis. 2004;2010(Dec. 2).
6. McClenaghan NH. Determining the relationship between dietary carbohydrate intake and insulin resistance. Nutr Res Rev. 2005;18(2):222–40.
7. Odegaard AO, Pereira MA. Trans fatty acids, insulin resistance, and type 2 diabetes. Nutr Rev. 2006;64(8):364–72.
8. Riserus U. Trans fatty acids and insulin resistance. Atheroscler Suppl. 2006;7(2):37–9.
9. McAuley K, Mann J. Thematic review series: patient-oriented research. Nutritional determinants of insulin resistance. J Lipid Res. 2006;47(8):1668–76.
10. Nordmann AJ, Nordmann A, Briel M, Keller U, Yancy Jr. WS, Brehm BJ, et al. Effects of low-

carbohydrate vs low-fat diets on weight loss and cardiovascular risk factors: a meta-analysis of randomized controlled trials. Arch Intern Med. 2006;166(3):285–93.
11. Galgani J, Uauy R, Aguirre CA, Díaz EO. Effect of the dietary fat quality on insulin sensitivity. Br J Nutr. 2008;100(3):471–9. doi:doi:10.1017/S0007114508894408
12. Kirk JK, Graves DE, Craven TE, Lipkin EW, Austin M, Margolis KL. Restricted-carbohydrate diets in patients with type 2 diabetes: a meta-analysis. J Am Diet Assoc. 2008;108(1):91–100.
13. Hartweg J, Perera R, Montori V, Dinneen S, Neil HA, Farmer A. Omega-3 polyunsaturated fatty acids (PUFA) for type 2 diabetes mellitus. Cochrane Database Syst Rev. 2008;(1):CD003205.
14. Risérus U, Willett WC, Hu FB, Riserus U, Willett WC, Hu FB. Dietary fats and prevention of type 2 diabetes. Prog Lipid Res. 2009;48(1):44–51. doi:10.1016/j.plipres.2008.10.002
15. Kodama S, Saito K, Tanaka S, Maki M, Yachi Y, Sato M, et al. Influence of Fat and Carbohydrate Proportions on the Metabolic Profile in Patients With Type 2 Diabetes: A Meta-Analysis. Diabetes Care. 2009;32(5):959–65. doi:10.2337/dc08-1716
16. Wallace SK, Mozaffarian D. Trans-fatty acids and nonlipid risk factors. Curr Atheroscler Rep. 2009;11(6):423–33.
17. Micha R, Mozaffarian D. Trans fatty acids: effects on metabolic syndrome, heart disease and diabetes. Nature reviews. Endocrinology. 2009;5(6):335–44. doi:10.1038/nrendo.2009.79
18. Micha R, Mozaffarian D. Saturated Fat and Cardiometabolic Risk Factors, Coronary Heart Disease, Stroke, and Diabetes: a Fresh Look at the Evidence. Lipids. 2010;45(10):893–905. doi:10.1007/s11745-010-3393-4
19. Thompson AK, Minihane AM, Williams CM. Trans fatty acids, insulin resistance and diabetes. Eur J Clin Nutr. 2010;
20. Akinkuolie AO, Ngwa JS, Meigs JB, Djoussé L. Omega-3 polyunsaturated fatty acid and insulin sensitivity: a meta-analysis of randomized controlled trials. Clin Nutr. 2011;30(6):702–7. doi:10.1016/j.clnu.2011.08.013
21. Matthan NR, Cianflone K, Lichtenstein AH, Ausman LM, Jauhiainen M, Jones PJ. Hydrogenated fat consumption affects acylation-stimulating protein levels and cholesterol esterification rates in moderately hypercholesterolemic women. J Lipid Res. 2001;42(11):1841–8.
22. Lovejoy JC, Windhauser MM, Rood JC, de la Bretonne JA. Effect of a controlled high-fat versus low-fat diet on insulin sensitivity and leptin levels in African-American and Caucasian women. Metabolism. 1998;47(12):1520–4.
23. Kratz M, Von Eckardstein A, Fobker M, Buyken A, Posny N, Schulte H, et al. The impact of dietary fat composition on serum leptin concentrations in healthy nonobese men and women. J Clin Endocrinol Metab. 2002;87(11):5008–14.
24. Kien CL, Bunn JY, Poynter ME, Stevens R, Bain J, Ikayeva O, et al. A lipidomics analysis of the relationship between dietary fatty acid composition and insulin sensitivity in young adults. Diabetes. 2013;62(4):1054–63. doi:10.2337/db12-0363
25. Lovejoy JC, Most MM, Lefevre M, Greenway FL, Rood JC. Effect of diets enriched in almonds on insulin action and serum lipids in adults with normal glucose tolerance or type 2 diabetes. Am J Clin Nutr. 2002;76(5):1000–6.
26. Sacks F, Carey V, Anderson C, PhD MPH, Miller E, Iii PhD MD, et al. Effects of High vs Low Glycemic Index of Dietary Carbohydrate on Cardiovascular Disease Risk Factors and Insulin Sensitivity: The OmniCarb Randomized Clinical Trial. JAMA. 2014;312(7501160):2531–41.
27. Jebb SA, Lovegrove JA, Griffin BA, Frost GS, Moore CS, Chatfield MD, et al. Effect of changing the amount and type of fat and carbohydrate on insulin sensitivity and cardiovascular risk: the RISCK (Reading, Imperial, Surrey, Cambridge, and Kings) trial. Am J Clin Nutr. 2010;92(4):748–58.
28. Higgins JPT, Green S. Cochrane Handbook for Systematic Reviews of Interventions. 1st ed. Higgins J, Green S, editors. West Sussex, England: The Cochrane Collaboration; 2008.
29. Poppitt SD, Keogh GF, Mulvey TB, McArdle BH, MacGibbon AK, Cooper GJ. Lipid-lowering effects of a modified butter-fat: a controlled intervention trial in healthy men. Eur J Clin Nutr. 2002;5(1):64–71.
30. Tardy AL, Lambert-Porcheron S, Malpuech-Brugère C, Giraudet C, Rigaudière JP, Laillet B, et al. Dairy and industrial sources of trans fat do not impair peripheral insulin sensitivity in overweight women. Am J Clin Nutr. 2009;90(1):88–94. doi:10.3945/ajcn.2009.27515
31. Vidon C, Boucher P, Cachefo A, Peroni O, Diraison F, Beylot M. Effects of isoenergetic high-carbohydrate compared with high-fat diets on human cholesterol synthesis and expression of key regulatory genes of cholesterol metabolism. Am J Clin Nutr. 2001;73(5):878–84.
32. Poppitt SD, Keogh GF, Mulvey TB, Phillips A, McArdle BH, MacGibbon AKH, et al. Effect of moderate

changes in dietary fatty acid profile on postprandial lipaemia, haemostatic and related CVD risk factors in healthy men. Eur J Clin Nutr. 2004;58(5):819–27. doi:10.1038/sj.ejcn.1601882
33. Sundram K, Karupaiah T, Hayes KC. Stearic acid-rich interesterified fat and trans-rich fat raise the LDL/HDL ratio and plasma glucose relative to palm olein in humans. Nutrition & metabolism. 2007;4:3. doi:10.1186/1743-7075-4-3
34. Noakes M, Foster PR, Keogh JB, James AP, Mamo JC, Clifton PM. Comparison of isocaloric very low carbohydrate/high saturated fat and high carbohydrate/low saturated fat diets on body composition and cardiovascular risk. Nutr Metab. 2006;3(7):7. doi:10.1186/1743-7075-3-7
35. Georgopoulos A, Bantle JP, Noutsou M, Swaim WR, Parker SJ. Differences in the metabolism of postprandial lipoproteins after a high-monounsaturated-fat versus a high-carbohydrate diet in patients with type 1 diabetes mellitus. Arterioscler Thromb Vasc Biol. 1998;18(5):773–82.
36. Nydahl MC, Gustafsson IB, Vessby B. Lipid-lowering diets enriched with monounsaturated or polyunsaturated fatty acids but low in saturated fatty acids have similar effects on serum lipid concentrations in hyperlipidemic patients. Am J Clin Nutr. 1994;59(1):115–22.
37. Tricon S, Burdge GC, Jones EL, Russell JJ, El-Khazen S, Moretti E, et al. Effects of dairy products naturally enriched with cis-9,trans-11 conjugated linoleic acid on the blood lipid profile in healthy middle-aged men. Am J Clin Nutr. 2006;83(4):744–53.
38. St-Onge MP, Newcomer BR, Buchthal S, Aban I, Allison DB, Bosarge A, et al. Intramyocellular lipid content is lower with a low-fat diet than with high-fat diets, but that may not be relevant for health. Am J Clin Nutr. 2007;86(5):1316–22.
39. Niskanen L, Schwab US, Sarkkinen ES, Krusius T, Vahtera E, Uusitupa MIJ. Effects of dietary fat modification on fibrinogen, factor VII, and plasminogen activator inhibitor-1 activity in subjects with impaired glucose tolerance. Metabolism. 1997;46(6):666–72. doi:10.1016/S0026-0495(97)90011-1
40. Iggman D, Gustafsson I-BB, Berglund L, Vessby B, Marckmann P, Risérus U, et al. Replacing dairy fat with rapeseed oil causes rapid improvement of hyperlipidaemia: a randomized controlled study. J Int Med. 2011;270(4):356–64. doi:10.1111/j.1365-2796.2011.02383.x
41. Gannon MC, Nuttall FQ. Effect of a high-protein, low-carbohydrate diet on blood glucose control in people with type 2 diabetes. Diabetes. 2004;53(9):2375–82.
42. Jeppesen J, Schaaf P, Jones C, Zhou MY, Chen YD, Reaven GM. Effects of low-fat, high-carbohydrate diets on risk factors for ischemic heart disease in postmenopausal women. Am J Clin Nutr. 1997;65(4):1027–33.
43. Schwab US, Karhapaa P, Sarkkinen ES, Salminen I, Laakso M, Uusitupa MIJ. Metabolic effects of diets rich in saturated and omega-6 polyunsatured fatty acids in healthy young females. Diab Nutr Metab. 1997;10(1):35–8.
44. Uusitupa M, Schwab U, Makimattila S, Karhapaa P, Sarkkinen E, Maliranta H, et al. Effects of two high-fat diets with different fatty acid compositions on glucose and lipid metabolism in healthy young women. Am J Clin Nutr. 1994;59:1310–6.
45. Gannon MC, Nuttall FQ. Effect of a high-protein diet on ghrelin, growth hormone, and insulin-like growth factor-I and binding proteins 1 and 3 in subjects with type 2 diabetes mellitus. Metabolism. 2011;60(9):1300–11. doi:S0026-0495(11)00030-8 [pii]10.1016/j.metabol.2011.01.016 [doi]
46. Goree LL, Chandler-Laney P, Ellis AC, Casazza K, Granger WM, Gower BA. Dietary macronutrient composition affects β cell responsiveness but not insulin sensitivity. Am J Clin Nutr. 2011;94(1):120–7. doi:10.3945/ajcn.110.002162
47. Yeung EH, Appel LJ, Miller ER, Kao WHL. The effects of macronutrient intake on total and high-molecular weight adiponectin: results from the OMNI-Heart trial. Obesity. 2010;18(8):1632–7. doi:10.1038/oby.2009.402
48. Bjermo H, Iggman D, Kullberg J, Dahlman I, Johansson L, Persson L, et al. Effects of n-6 PUFAs compared with SFAs on liver fat, lipoproteins, and inflammation in abdominal obesity: a randomized controlled trial. Am J Clin Nutr. 2012;95(5):1003–12. doi:10.3945/ajcn.111.030114
49. Haghighatdoost F, Hosseinzadeh-Attar MJ, Kabiri A, Eshraghian M, Esmaillzadeh A. Effect of substituting saturated with monounsaturated fatty acids on serum visfatin levels and insulin resistance in overweight women: A randomized cross-over clinical trial. Int J Food Sci Nutr. 2012;63(7):772–81. doi:10.3109/09637486.2012.665044
50. Chiu S, Williams PT, Dawson T, Bergman RN, Stefanovski D, Watkins SM, et al. Diets High in Protein or Saturated Fat Do Not Affect Insulin Sensitivity or Plasma Concentrations of Lipids and Lipoproteins in Overweight and Obese Adults. J Nutr. 2014;144(11):1753–9. doi:10.3945/jn.114.197624
51. Iggman D, Rosqvist F, Larsson A, Arnlov J, Beckman L, Rudling M, et al. Role of Dietary Fats in

Modulating Cardiometabolic Risk During Moderate Weight Gain: A Randomized Double-Blind Overfeeding Trial (LIPOGAIN Study). J Am Heart Assoc. 2014;3(5). doi:10.1161/jaha.114.001095
52. Baril-Gravel L, Labonte ME, Couture P, Vohl M-CC, Charest A, Guay V, et al. Docosahexaenoic acid-enriched canola oil increases adiponectin concentrations: a randomized crossover controlled intervention trial. Nutr Metab Cardiovasc Dis. 2015;25(1):52–9. doi:10.1016/j.numecd.2014.08.003
53. Kriketos a D, Robertson RM, Sharp TA, Drougas H, Reed GW, Storlien LH, et al. Role of weight loss and polyunsaturated fatty acids in improving metabolic fitness in moderately obese, moderately hypertensive subjects. J Hypertens. 2001;19(10):1745–54. doi:10.1097/00004872-200110000-00007
54. Eckel RH, Hernandez TL, Bell ML, Weil KM, Shepard TY, Grunwald GK, et al. Carbohydrate balance predicts weight and fat gain in adults. Am J Clin Nutr. 2006;83(4):803–8.
55. Coulston AM, Hollenbeck CB, Swislocki AL, Reaven GM. Persistence of hypertriglyceridemic effect of low-fat high-carbohydrate diets in NIDDM patients. Diabetes Care. 1989;12(2):94–101.
56. Coulston AM, Liu GC, Reaven GM. Plasma glucose, insulin and lipid responses to high-carbohydrate low-fat diets in normal humans. Metabolism. 1983;32:52–6.
57. Fuh MM, Lee MM, Jeng CY, Ma F, Chen YD, Reaven GM. Effect of low fat-high carbohydrate diets in hypertensive patients with non-insulin-dependent diabetes mellitus. Am J Hypertens. 1990;3(7):527–32.
58. Johnston CS, Tjonn SL, Swan PD. High-protein, low-fat diets are effective for weight loss and favorably alter biomarkers in healthy adults. J Nutr. 2004;134(3):586–91.
59. Nuttall FQ, Schweim K, Hoover H, Gannon MC. Effect of the LoBAG30 diet on blood glucose control in people with type 2 diabetes. Br J Nutr. 2008;99(3):511–9. doi:10.1017/S0007114507819155
60. Brynes AE, Edwards CM, Jadhav A, Ghatei MA, Bloom SR, Frost GS. Diet-induced change in fatty acid composition of plasma triacylglycerols is not associated with change in glucagon-like peptide 1 or insulin sensitivity in people with type 2 diabetes. Am J Clin Nutr. 2000;72(5):1111–8.
61. Garg A, Grundy SM, Unger RH. Comparison of effects of high and low carbohydrate diets on plasma lipoproteins and insulin sensitivity in patients with mild NIDDM. Diabetes. 1992;41(10):1278–85.
62. Bradley U, Spence M, Courtney CH, McKinley MC, Ennis CN, McCance DR, et al. Low-fat versus low-carbohydrate weight reduction diets: effects on weight loss, insulin resistance, and cardiovascular risk: a randomized control trial. Diabetes. 2009;58(12):2741–8.
63. Thomsen C, Rasmussen OW, Ingerslev J, Hermansen K. Plasma-Levels of Von-Willebrand-Factor in Non-Insulin-Dependent Diabetes-Mellitus Are Influenced by Dietary Monounsaturated Fatty-Acids. Thrombosis Res. 1995;77(4):347–56.
64. Papakonstantinou E, Triantafillidou D, Panagiotakos DB, Koutsovasilis A, Saliaris M, Manolis a, et al. A high-protein low-fat diet is more effective in improving blood pressure and triglycerides in calorie-restricted obese individuals with newly diagnosed type 2 diabetes. Eur J Clin Nutr. 2010;64(6):595–602. doi:10.1038/ejcn.2010.29
65. Shige H, Nestel P, Sviridov D, Noakes M, Clifton P. Effect of weight reduction on the distribution of apolipoprotein A-I in high-density lipoprotein subfractions in obese non-insulin-dependent diabetic subjects. Metabolism. 2000;49(11):1453–9.
66. Louheranta AM, Turpeinen AK, Schwab US, Vidgren HM, Parviainen MT, Uusitupa MIJ. A high-stearic acid diet does not impair glucose tolerance and insulin sensitivity in healthy women. Metabolism. 1998;47(5):529–34. doi:10.1016/S0026-0495(98)90235-9
67. Rasmussen OW, Thomsen C, Hansen KW, Vesterlund M, Winther E, Hermansen K. Effects on blood pressure, glucose, and lipid levels of a high-monounsaturated fat diet compared with a high-carbohydrate diet in NIDDM subjects. Diabetes Care. 1993;16(12):1565–71.
68. Schwab US, Niskanen LEOK, Maliranta HM, Savolainen MJ, Kesaniemi YA, Uusitupa MI, et al. Lauric and palmitic acid-enriched diets have minimal impact on serum lipid and lipoprotein concentrations and glucose metabolism in healthy young women. J Nutr. 1995;125(3):466–73.
69. Louheranta AM, Schwab US, Sarkkinen ES, Voutilainen ET, Ebeling TM, Erkkil・At, et al. Insulin sensitivity after a reduced-fat diet and a monoene-enriched diet in subjects with elevated serum cholesterol and triglyceride concentrations. Nutr Metab Cardiovasc Dis. 2000;10(4):177–87.
70. Louheranta AM, Turpeinen AK, Vidgren HM, Schwab US, Uusitupa MIJ, Design S. A high-trans fatty acid diet and insulin sensitivity in young healthy women. Metabolism. 1999;48(7):870–5. doi:10.1016/S0026-0495(99)90221-4
71. Lovejoy JC, Smith SR, Champagne CM, Most MM, Lefevre M, DeLany JP, et al. Effects of diets enriched in saturated (palmitic), monounsaturated (oleic), or trans (elaidic) fatty acids on insulin sensitivity and substrate oxidation in healthy adults. Diabetes care. 2002;25(8):1283–8.

72. Thomsen C, Rasmussen O, Christiansen C, Pedersen E, Vesterlund M, Storm H, et al. Comparison of the effects of a monounsaturated fat diet and a high carbohydrate diet on cardiovascular risk factors in first degree relatives to type-2 diabetic subjects. Eur J Clin Nutr. 1999;53(10):818–23. doi:10.1038/sj.ejcn.1600855
73. Furukawa TA, Barbui C, Cipriani A, Brambilla P, Watanabe N. Imputing missing standard deviations in meta-analyses can provide accurate results. J Clin Epidemiol. 2006;59(1):7–10. doi:10.1016/j.jclinepi.2005.06.006
74. Thiessen Philbrook H, Barrowman N, Garg AX. Imputing variance estimates do not alter the conclusions of a meta-analysis with continuous outcomes: a case study of changes in renal function after living kidney donation. J Clin Epidemiol. 2007;60(3):228–40. doi:10.1016/j.jclinepi.2006.06.018
75. Elbourne DR. Meta-analyses involving cross-over trials: methodological issues. International Journal of Epidemiology. 2002;31(1):140–9. doi:10.1093/ije/31.1.140
76. Higgins J. Chapter 9 : Analysing data and undertaking. In: Higgins J, Green S, editors. Cochrane Handbook for Systematic Reviews of Interventions. West Sussex, England: The Cochrane Collaboration; 2008. p. 1–43.
77. Luscombe-Marsh ND, Noakes M, Wittert GA, Keogh JB, Foster P, Clifton PM. Carbohydrate-restricted diets high in either monounsaturated fat or protein are equally effective at promoting fat loss and improving blood lipids. Am J Clin Nutr. 2005;81(4):762–72.
78. Noakes M, Keogh JB, Foster PR, Clifton PM. Effect of an energy-restricted, high-protein, low-fat diet relative to a conventional high-carbohydrate, low-fat diet on weight loss, body composition, nutritional status, and markers of cardiovascular health in obese women. Am J Clin Nutr. 2005;81(6):1298–306.
79. van Dijk SJ, Feskens EJM, Bos MB, Hoelen DWM, Heijligenberg R, Bromhaar MG, et al. A saturated fatty acid – rich diet induces an obesity-linked proinflammatory gene expression profile in adipose tissue of subjects at risk of metabolic syndrome 1 – 3. Am J Clin Nutr. 2009;90:1656–64. doi:10.3945/ajcn.2009.27792.INTRODUCTION
80. Gustafsson IB, Vessby B, Nydahl M. Effects of lipid-lowering diets enriched with monounsaturated and polyunsaturated fatty acids on serum lipoprotein composition in patients with hyperlipoproteinaemia. Atherosclerosis. 1992;96(2-3):109–18.
81. Bradley U, Spence M, Courtney CH, Mckinley MC, Ennis CN, Mccance DR, et al. Effects on Weight Loss, Insulin Resistance, and Cardiovascular Risk: A Randomized Control Trial. Diabetes. 2009;58(December):2741–8. doi:10.2337/db09-0098.
82. Walker KZ, O'Dea K, Nicholson GC, Muir JG. Dietary composition, body weight, and NIDDM: Comparison of high-fiber, high-carbohydrate, and modified-fat diets. Diabetes Care. 1995;18(3):401–3.
83. Lerman-Garber I, Gulias-Herrero A, Palma ME, Valles VE, Guerrero LA, Garcia EG, et al. Response to high carbohydrate and high monounsaturated fat diets in hypertriglyceridemic non-insulin dependent diabetic patients with poor glycemic control. Diab Nutr Metab. 1995;8(6):339–45.
84. Vessby B, Karlstrom B, Boberg M, Lithell H, Berne C. Polyunsaturated fatty acids may impair blood glucose control in Type 2 diabetic patients. Diabetic medicine. 1992;9(2):126–33.
85. Mensink RP. Effects of products made from a high-palmitic acid, trans-free semiliquid fat or a high-oleic acid, low-trans semiliquid fat on the serum lipoprotein profile and on C-reactive protein concentrations in humans. Eur J Clin Nutr. 2008;62(5):617–24. doi:10.1038/sj.ejcn.1602756
86. Hwalla N, Torbay N, Andari N, Adra N, Azar ST, Habbal Z. Restoration of normal insulinemia and insulin sensitivity in hyperinsulinemic normoglycemic men by a hypoenergetic high monounsaturated fat diet. J Nutr Environ Med. 2004;14(1):29–38.
87. Segal-Isaacson CJ, Johnson S, Tomuta V, Cowell B, Stein DT. A Randomized Trial Comparing Low-Fat and Low-Carbohydrate Diets Matched for Energy and Protein. Obes Res. 2004;12 Suppl 2:130S – 40S.
88. Garg A, Bantle JP, Henry RR, Coulston AM, Griver KA, Raatz SK, et al. Effects of varying carbohydrate content of diet in patients with non-insulin-dependent diabetes mellitus. JAMA. 1994;271(18):1421–8.
89. Parillo M, Rivellese AA, Ciardullo A V, Capaldo B, Giacco A, Genovese S, et al. A high-monounsaturated-fat/low-carbohydrate diet improves peripheral insulin sensitivity in non-insulin-dependent diabetic patients. Metabolism. 1992;41(12):1373–8.
90. Rodriguez-Villar C, Perez-Heras A, Mercade I, Casals E, Ros E. Comparison of a high-carbohydrate and a high-monounsaturated fat, olive oil-rich diet on the susceptibility of LDL to oxidative modification in subjects with Type 2 diabetes mellitus. Diabet Med. 2004;21(2):142–9.
91. Bendsen NT, Haugaard SB, Larsen TM, Chabanova E, Stender S, Astrup A. Effect of trans-fatty acid intake on insulin sensitivity and intramuscular lipids - A randomized trial in overweight postmenopausal women.

Metabolism. 2011;60(7):906–13. doi:10.1016/j.metabol.2011.01.009
92. Johnstone AM, Lobley GE, Horgan GW, Bremner DM, Fyfe CL, Morrice PC, et al. Effects of a high-protein, low-carbohydrate v. high-protein, moderate-carbohydrate weight-loss diet on antioxidant status, endothelial markers and plasma indices of the cardiometabolic profile. Br J Nutr. 2011;106(2):282–91. doi:10.1017/S0007114511000092
93. Li S-C, Liu Y-H, Liu J-F, Chang W-H, Chen C-M, Chen C-YO. Almond consumption improved glycemic control and lipid profiles in patients with type 2 diabetes mellitus. Metabolism. 2011;60(4):474–9. doi:10.1016/j.metabol.2010.04.009
94. Young LR, Kurzer MS, Thomas W, Redmon JB, Raatz SK. Low-fat diet with omega-3 fatty acids increases plasma insulin-like growth factor concentration in healthy postmenopausal women. Nutr Res. 2013;33(7):565–71. doi:10.1016/j.nutres.2013.04.011
95. Tay J, Luscombe-Marsh ND, Thompson CH, Noakes M, Buckley JD, Wittert GA, et al. Comparison of low- and high-carbohydrate diets for type 2 diabetes management: a randomized trial. Am J Clin Nutr. 2015;102(4):780–90. doi:10.3945/ajcn.115.112581
96. Vafeiadou K, Weech M, Altowaijri H, Todd S, Yaqoob P, Jackson KG, et al. Replacement of saturated with unsaturated fats had no impact on vascular function but beneficial effects on lipid biomarkers, E-selectin, and blood pressure: results from the randomized, controlled Dietary Intervention and VAScular function (DIVAS) study. Am J Clin Nutr. 2015;102(1):40–8. doi:10.3945/ajcn.114.097089
97. Tholstrup T, Raff M, Basu S, Nonboe P, Sejrsen K, Straarup EM. Effects of butter high in ruminant trans and monounsaturated fatty acids on lipoproteins, incorporation of fatty acids into lipid classes, plasma C-reactive protein, oxidative stress, hemostatic variables, and insulin in healthy young men. Am J Clin Nutr. 2006;83(2):237–43. doi:83/2/237 [pii]
98. Hjerpsted J, Leedo E, Tholstrup T. Cheese intake in large amounts lowers LDL-cholesterol concentrations compared with butter intake of equal fat content. Am J Clin Nutr. 2011;94(6):1479–84.
99. Helge JW, Tobin L, Drachmann T, Hellgren LI, Dela F, Galbo H. Muscle ceramide content is similar after 3 weeks' consumption of fat or carbohydrate diet in a crossover design in patients with type 2 diabetes. Eur J Appl Physiol. 2012;112(3):911–8. doi:10.1007/s00421-011-2041-x
100. Filippou A, Teng KT, Berry S, Sanders T. Palmitic acid in the sn-2 position of dietary triacylglycerols does not affect insulin secretion or glucose homeostasis in healthy men and women. Eur J Clin Nutr. 2014;68(ejc, 8804070):1036–41.
101. Rozati M, Barnett J, Wu DY, Handelman G, Saltzman E, Wilson T, et al. Cardio-metabolic and immunological impacts of extra virgin olive oil consumption in overweight and obese older adults: a randomized controlled trial. Nutr Metabolism. 2015;12. doi:10.1186/s12986-015-0022-5
102. Lerman-Garber I, Ichazo-Cerro S, Zamora-Gonzalez J, Cardoso-Saldana G, Posadas-Romero C. Effect of a high-monounsaturated fat diet enriched with avocado in NIDDM patients. Diabetes Care. 1994;17(4):311–5.
103. Higashi K, Shige H, Ito T, Nakajima K, Ishikawa T, Nakamura H, et al. Effect of a low-fat diet enriched with oleic acid on postprandial lipemia in patients with type 2 diabetes mellitus. Lipids. 2001;36(1):1–6.
104. Katsilambros N, Kostalas G, Michalakis N, Kapantais E, Mangalara E, Kouzeli C, et al. Metabolic effects of long-term diets enriched in olive oil or sunflower oil in non-insulin-dependent diabetes. Nutr Metab Cardiovasc Dis. 1996;6(3):164–7.
105. Berglund L, Lefevre M, Ginsberg HN, Kris-Etherton PM, Elmer PJ, Stewart PW, et al. Comparison of monounsaturated fat with carbohydrates as a replacement for saturated fat in subjects with a high metabolic risk profile: studies in the fasting and postprandial states. Am J Clin Nutr. 2007;86(6):1611–20.
106. Egert S, Fobker M, Andersen G, Somoza V, Erbersdobler HF, Wahrburg U. Effects of dietary alpha-linolenic acid, eicosapentaenoic acid or docosahexaenoic acid on parameters of glucose metabolism in healthy volunteers. Ann Nutr Metab. 2008;53(3-4):182–7.
107. Vega-Lopez S, Ausman LM, Jalbert SM, Erkkila AT, Lichtenstein AH, Erkkil?? AT, et al. Palm and partially hydrogenated soybean oils adversely alter lipoprotein profiles compared with soybean and canola oils in moderately hyperlipidemic subjects. Am J Clin Nutr. 2006;84(1):54–62.
108. Paniagua J a, de la Sacristana AG, Sanchez E, Romero I, Vidal-Puig A, Berral FJ, et al. A MUFA-rich diet improves posprandial glucose, lipid and GLP-1 responses in insulin-resistant subjects. J Am Coll Nutr. 2007;26(5):434–44. doi:10.1080/07315724.2007.10719633
109. Guay V, Lamarche B, Charest A, Tremblay AJ, Couture P. Effect of short-term low- and high-fat diets on low-density lipoprotein particle size in normolipidemic subjects. Metabolism. 2012;61(1):76–83. doi:10.1016/j.metabol.2011.06.002
110. Mensink RP, Zock PL, Kester ADM, Katan MB. Effects of dietary fatty acids and carbohydrates on the ratio

of serum total to HDL cholesterol and on serum lipids and apolipoproteins: a meta-analysis of 60 controlled trials. Am J Clin Nutr. 2003;77(5):1146–55.
111. Clarke R, Frost C, Collins R, Appleby P, Peto R. Dietary lipids and blood cholesterol: quantitative meta-analysis of metabolic ward studies. BMJ. 1997;314(7074):112. doi:10.1136/bmj.314.7074.112
112. Nydahl M, Gustafsson IB, Ohrvall M, Vessby B. Similar effects of rapeseed oil (canola oil) and olive oil in a lipid- lowering diet for patients with hyperlipoproteinemia. J Am Coll Nutr. 1995;14(6):643–51.
113. Vega-Lopez S, Matthan NR, Ausman LM, Ai M, Otokozawa S, Schaefer EJ, et al. Substitution of vegetable oil for a partially-hydrogenated fat favorably alters cardiovascular disease risk factors in moderately hypercholesterolemic postmenopausal women. Atherosclerosis. 2009;207(1):208–12. doi:10.1016/j.atherosclerosis.2009.03.039
114. Forsythe CE, Phinney SD, Feinman RD, Volk BM, Freidenreich D, Quann E, et al. Limited effect of dietary saturated fat on plasma saturated fat in the context of a low carbohydrate diet. Lipids. 2010;45(10):947–62. doi:10.1007/s11745-010-3467-3
115. Christiansen E, Schnider S, Palmvig B, Tauber-Lassen E, Pedersen O. Intake of a diet high in trans monounsaturated fatty acids or saturated fatty acids. Effects on postprandial insulinemia and glycemia in obese patients with NIDDM. Diabetes Care. 1997;20(5):881–7.
116. White IR, Royston P, Wood AM. Multiple imputation using chained equations: Issues and guidance for practice. Stat Med. 2011;30(4):377–99. doi:10.1002/sim.4067
117. Espino-Montoro A, Lopez-Miranda J, Castro P, Rodriguez M, LopezSegura F, Blanco A, et al. Monounsaturated fatty acid enriched diets lower plasma insulin levels and blood pressure in healthy young men. Nutr Metab Cardiovasc Dis. 1996;6(3):147–54.
118. Pérez-Jiménez F, López-Miranda J, Pinillos MD, Gómez P, Paz-Rojas E, Montilla P, et al. A Mediterranean and a high-carbohydrate diet improve glucose metabolism in healthy young persons. Diabetologia. 2001;44(11):2038–43.
119. Clifton PM, Noakes M, Keogh JB. Very low-fat (12%) and high monounsaturated fat (35%) diets do not differentially affect abdominal fat loss in overweight, nondiabetic women. J Nutr. 2004;134(7):1741–5.
120. Keogh JB, Grieger JA, Noakes M, Clifton PM. Flow-mediated dilatation is impaired by a high-saturated fat diet but not by a high-carbohydrate diet. Arterioscler Thromb Vasc Biol. 2005;25(6):1274–9.
121. Schutte AE, Van Rooyen JM, Huisman HW, Mukuddem-Petersen J, Oosthuizen W, Hanekom SM, et al. Modulation of baroreflex sensitivity by walnuts versus cashew nuts in subjects with metabolic syndrome. Am J Hypertens. 2006;19(6):629–36.
122. Tay J, Brinkworth GD, Noakes M, Keogh J, Clifton PM. Metabolic Effects of Weight Loss on a Very-Low-Carbohydrate Diet Compared With an Isocaloric High-Carbohydrate Diet in Abdominally Obese Subjects. J Am Coll Cardiol. 2008;51(1):59–67.
123. Bos MB, de Vries JHM, Feskens EJM, van Dijk SJ, Hoelen DWM, Siebelink E, et al. Effect of a high monounsaturated fatty acids diet and a Mediterranean diet on serum lipids and insulin sensitivity in adults with mild abdominal obesity. Nutr Metab Cardiovasc Dis. 2010;20(8):591–8. doi:10.1016/j.numecd.2009.05.008
124. Tholstrup T, Hjerpsted J, Raff M. Palm olein increases plasma cholesterol moderately compared with olive oil in healthy individuals. Am J Clin Nutr. 2011;94(6):1426–32.
125. Tierney a C, McMonagle J, Shaw DI, Gulseth HL, Helal O, Saris WHM, et al. Effects of dietary fat modification on insulin sensitivity and on other risk factors of the metabolic syndrome—LIPGENE: a European randomized dietary intervention study. Int J Obes. 2011;35(6):800–9. doi:10.1038/ijo.2010.209
126. Wycherley TP, Brinkworth GD, Clifton PM, Noakes M. Comparison of the effects of 52 weeks weight loss with either a high-protein or high-carbohydrate diet on body composition and cardiometabolic risk factors in overweight and obese males. Nutr Diabetes. 2012;2:e40. doi:10.1038/nutd.2012.11
127. Jordy AB, Serup AK, Karstoft K, Pilegaard H, Kiens B, Jeppesen J. Insulin sensitivity is independent of lipid binding protein trafficking at the plasma membrane in human skeletal muscle: effect of a 3-day, high-fat diet. Am J Physiol Regul Integr Comp Physiol. 2014;307(9):R1136–45. doi:10.1152/ajpregu.00124.2014
128. Marina A, von Frankenberg AD, Suvag S, Callahan HS, Kratz M, Richards TL, et al. Effects of Dietary Fat and Saturated Fat Content on Liver Fat and Markers of Oxidative Stress in Overweight/Obese Men and Women under Weight-Stable Conditions. Nutrients. 2014;6(11):4678–90. doi:10.3390/nu6114678
129. Rietman A, Schwarz J, Blokker BA, Siebelink E, Kok FJ, Afman LA, et al. Increasing Protein Intake Modulates Lipid Metabolism in Healthy Young Men and Women Consuming a High-Fat Hypercaloric Diet. J Nutr. 2014;144(8):1174–80. doi:10.3945/jn.114.191072
130. Garg A, Bonanome A, Grundy SM, Zhang ZJ, Unger RH. Comparison of a high-carbohydrate diet with a

high-monounsaturated-fat diet in patients with non-insulin-dependent diabetes mellitus. New Engl J Med. 1988. doi:10.1056/NEJM198809293191304
131. Garg A, Grundy SM, Koffler M. Effect of high carbohydrate intake on hyperglycemia, islet function, and plasma lipoproteins in NIDDM. Diabetes Care. 1992;15(11):1572–80.
132. Storm H, Thomsen C, Pedersen E, Rasmussen O, Christiansen C, Hermansen K. Comparison of a carbohydrate-rich diet and diets rich in stearic or palmitic acid in NIDDM patients. Effects on lipids, glycemic control, and diurnal blood pressure. Diabetes Care. 1997;20(12):1807–13.
133. Miyashita Y, Koide N, Ohtsuka M, Ozaki H, Itoh Y, Oyama T, et al. Beneficial effect of low carbohydrate in low calorie diets on visceral fat reduction in type 2 diabetic patients with obesity. Diabetes Res Clin Pract. 2004;65(3):235–41. doi:10.1016/j.diabres.2004.01.008
134. Papakonstantinou E, Triantafillidou D, Panagiotakos DB, Iraklianou S, Berdanier CD, Zampelas A. A high protein low fat meal does not influence glucose and insulin responses in obese individuals with or without type 2 diabetes. J Hum Nutr Diet. 2010;23(2):183–9.
135. Taylor CG, Noto AD, Stringer DM, Froese S, Malcolmson L. Dietary milled flaxseed and flaxseed oil improve N-3 fatty acid status and do not affect glycemic control in individuals with well-controlled type 2 diabetes. J Am Coll Nutr. 2010;29(1):72–80.
136. Jenkins DJ a, Kendall CWC, Banach MS, Srichaikul K, Vidgen E, Mitchell S, et al. Nuts as a replacement for carbohydrates in the diabetic diet. Diabetes Care. 2011;34(8):1706–11. doi:dc11-0338 [pii]10.2337/dc11-0338 [doi]
137. Moher D, Jadad AR, Nichol G, Penman M, Tugwell P, Walsh S. Assessing the quality of randomized controlled trials: An annotated bibliography of scales and checklists. Controlled Clinical Trials. 1995;16(1):62–73.
138. Jadad AR, Moore RA, Carroll D, Jenkinson C, Reynolds DJM, Gavaghan DJ, et al. Assessing the quality of reports of randomized clinical trials: Is blinding necessary? Control Clin Trials. 1996;17(1):1–12. doi:10.1016/0197-2456(95)00134-4
139. Higgins JP, Altman DG, Gotzsche PC, Juni P, Moher D, Oxman AD, et al. The Cochrane Collaboration's tool for assessing risk of bias in randomised trials. BMJ. 2011;343:d5928. doi:10.1136/bmj.d5928
